# Preoperative MRI features and clinical factors for predicting early recurrence of hepatocellular carcinoma after hepatectomy

**DOI:** 10.3389/fonc.2026.1939820

**Published:** 2026-09-17

**Authors:** Quanyou Miao, Chaofeng Yang, Ling Yang, Anqi Chen, Jun Xiao, Gaowu Yan, Jinhong Yu, Yang Li

**Affiliations:** 1 Department of Radiology, Affiliated Hospital of North Sichuan Medical College, Nanchong, Sichuan, China; 2 Department of Pathology, Suining Central Hospital, Suining, Sichuan, China; 3 School of Medical Imaging, North Sichuan Medical College, Nanchong, Sichuan, China; 4 Department of Radiology, Suining Central Hospital, Suining, Sichuan, China; 5 Department of Ultrasound, Affiliated Hospital of North Sichuan Medical College, Nanchong, Sichuan, China

**Keywords:** early recurrence, hepatectomy, hepatocellular carcinoma, magnetic resonance imaging, prediction model

## Abstract

**Introduction:**

Early recurrence after hepatectomy remains a major obstacle to durable disease control in hepatocellular carcinoma (HCC). This study developed and externally validated a prediction model based on preoperative MRI features and clinical laboratory factors for early recurrence after hepatectomy for HCC, defined as recurrence within 24 months after surgery.

**Methods:**

This two-center retrospective cohort study included adult patients with pathologically confirmed HCC who underwent curative hepatectomy and contrast-enhanced liver MRI within 2 months before surgery between January 2018 and December 2025. The Affiliated Hospital of North Sichuan Medical College served as the development cohort, and Suining Central Hospital served as the external validation cohort. Candidate clinical and MRI predictors were evaluated in the development cohort. A Cox regression model was developed, internally validated with 1000 bootstrap resamples, and directly applied to the external validation cohort without refitting.

**Results:**

The final analysis cohort included 320 patients, including 258 in the development cohort and 62 in the external validation cohort. Overall, 183 patients (57.2%) experienced early recurrence. The final combined Cox model retained log(PIVKA-II), tumor size >5 cm, satellite nodule, peritumoral hypointensity on portal venous phase images, intrahepatic vessel invasion, and incomplete capsule. In the development cohort, the apparent C-index was 0.756, and the bootstrap-corrected 24-month AUC was 0.829. In the external validation cohort, the C-index was 0.892, the 24-month AUC was 0.978, and the Brier score was 0.087. Calibration analysis indicated imperfect absolute risk calibration, with a calibration slope of 2.705 and an intercept of -1.288; grouped calibration suggested risk overestimation in the lower predicted-risk range. The development cohort-derived cutoff of 0.584 separated patients with different early recurrence risks in both cohorts. In the external validation cohort, only one early recurrence event occurred in the low-risk reference group, so the risk-stratification HR should be interpreted as directional separation rather than as a stable clinical effect-size estimate.

**Conclusion:**

This preoperative MRI-clinical Cox model showed good predictive performance for early recurrence after hepatectomy for HCC and high discrimination in external validation. Its calibration and clinical utility require further evaluation in larger prospective multicenter cohorts.

## 1 Introduction

Hepatocellular carcinoma is one of the leading causes of cancer-related death worldwide (1). Global cancer statistics for 2022 showed that liver cancer continues to account for a substantial number of incident cases and deaths, and many patients remain candidates for potentially curative treatment only within a limited disease window (1–3). Hepatectomy is an important curative treatment option for selected patients with HCC (2, 3). However, postoperative recurrence remains common and is a major cause of treatment failure. Recurrence within 2 years after surgery is generally regarded as early recurrence and is often associated with aggressive tumor biology, occult intrahepatic dissemination, and poorer outcomes (4–6).

Preoperative risk assessment may help identify patients at high risk of recurrence before treatment, thereby informing follow-up intensity, multidisciplinary discussion, and perioperative treatment research (3, 5). Clinical laboratory indicators can capture aspects of tumor biology, liver functional reserve, and host inflammatory status beyond imaging. Serum biomarkers such as PIVKA-II and AFP are associated with tumor burden, aggressive phenotypes, and early recurrence risk after HCC resection (5–7). Liver function and inflammation-related indicators may also provide complementary information for postoperative recurrence risk assessment (5, 8). However, these indicators cannot directly depict spatial tumor heterogeneity, local invasion patterns, or peritumoral microenvironmental changes. They therefore need to be complemented by preoperative imaging information.

MRI can noninvasively depict morphologic tumor burden and aggressive imaging phenotypes of HCC before surgery. Previous studies have shown that preoperative MRI features, including tumor size, margin- or capsule-related findings, satellite nodules, imaging signs related to vascular invasion, and peritumoral abnormalities, are associated with early recurrence after HCC resection (9–13). These imaging findings provide local tumor and peritumoral information that is not directly captured by clinical laboratory indicators. Thus, integrating preoperative clinical laboratory factors with routinely assessable MRI features may better characterize early recurrence risk in HCC.

On this basis, this study aimed to develop and externally validate a combined prediction model based on preoperative clinical laboratory factors and routinely assessable MRI features for predicting recurrence within 24 months after hepatectomy for HCC. A two-center development and external validation design was used to compare clinical, MRI, and combined models and to evaluate discrimination, calibration, and risk stratification, in accordance with principles for external validation and transportability assessment of prediction models (14, 15).

## 2 Materials and methods

### 2.1 Study design

This two-center retrospective cohort study was designed to develop and externally validate a preoperative prediction model for early recurrence after curative hepatectomy for HCC (14, 15). The Affiliated Hospital of North Sichuan Medical College was used as the development cohort, and Suining Central Hospital was used as the external validation cohort. The study followed the TRIPOD reporting framework for multivariable prediction models (14). This retrospective study used de-identified clinical, imaging, pathological, and follow-up data collected during routine care, and no prospective intervention was performed.

### 2.2 Study patients

Patients from the development and external validation cohorts were consecutively screened between January 2018 and December 2025. The inclusion criteria were based on the general framework for curative resection eligibility in HCC management guidelines (2, 3) and were as follows: 1) adult patients; 2) curative hepatectomy; 3) pathologically confirmed HCC after surgery; 4) contrast-enhanced liver MRI performed within 2 months before surgery; and 5) available preoperative clinical laboratory data, MRI data, and postoperative follow-up data. In this study, curative hepatectomy required all of the following: R0 resection with pathologically negative surgical margins; no unresectable distant metastasis or definite residual tumor found intraoperatively; and no definite residual active tumor on early postoperative baseline imaging. Early postoperative baseline imaging was defined as dynamic contrast-enhanced CT or MRI combined with AFP assessment approximately 1 month after surgery. If definite residual active disease was present at this time point, the patient was considered to have residual disease or noncurative resection and was not classified as having postoperative recurrence. A total of 445 patients were initially screened, including 334 from the development cohort source center and 111 from the external validation source center.

The exclusion criteria were as follows: 1) previous HCC-related treatment, including surgery, interventional therapy, or systemic therapy; 2) current or previous malignancy other than HCC; 3) ruptured HCC; 4) concomitant interventional therapy during hepatectomy; 5) insufficient MRI image quality for feature assessment; and 6) unavailable postoperative follow-up information. According to these criteria, 94 patients were excluded: 42 for previous HCC treatment, 13 for other malignancies, three for ruptured HCC, 21 for concomitant interventional therapy during surgery, and 15 for insufficient MRI image quality. After these exclusions, 351 patients were eligible for postoperative follow-up assessment, including 272 from the primary center and 79 from the external validation center. A further 31 patients were excluded because follow-up information was unavailable, including 14 from the primary center and 17 from the external validation center. The final analysis included 320 patients, consisting of 258 in the development cohort and 62 in the external validation cohort. The patient selection process is shown in **Figure 1**.

**Figure 1:**
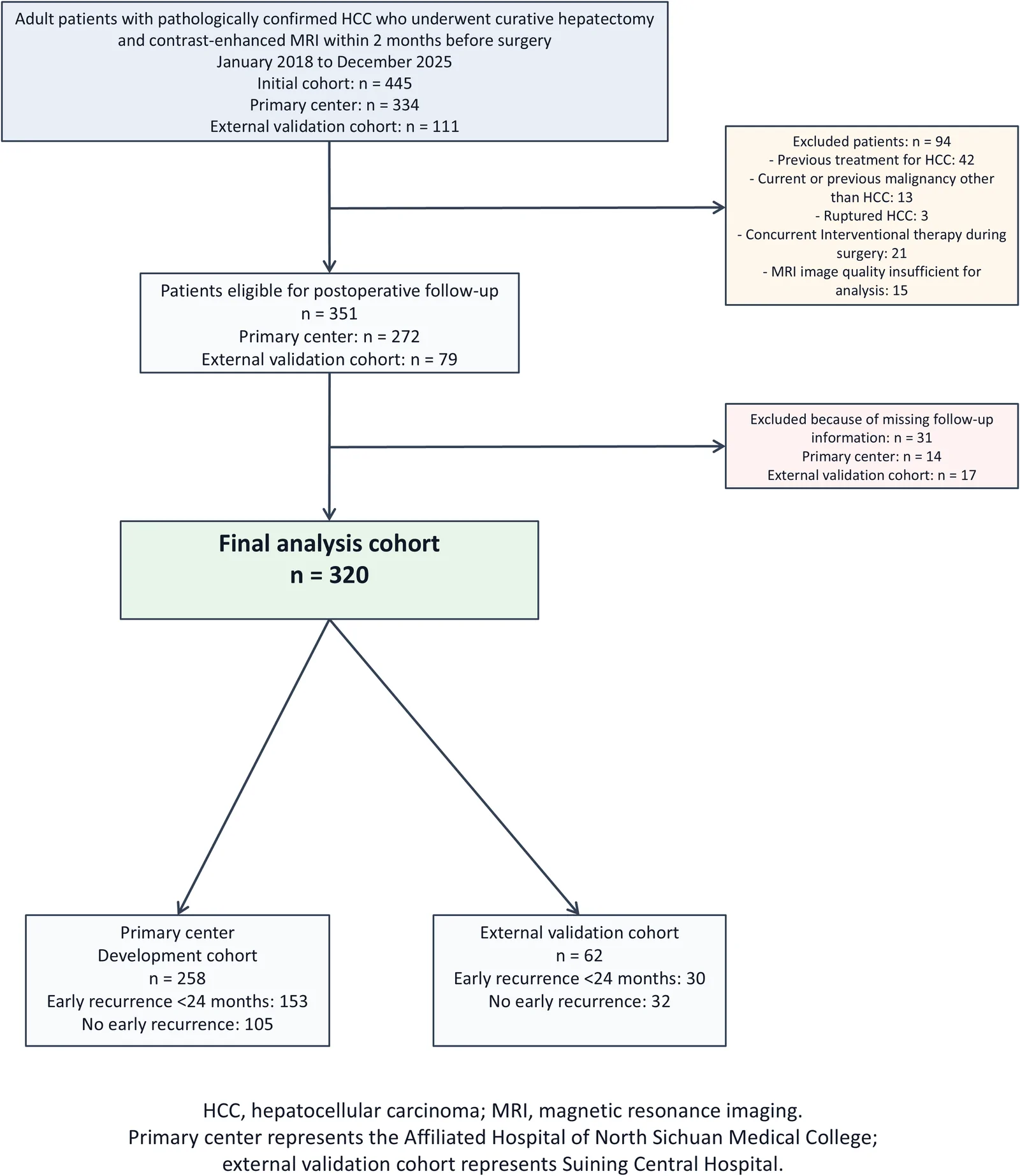
Study inclusion and exclusion flowchart. The flowchart shows patient screening, exclusion, follow-up eligibility assessment, and final cohort allocation at the two centers from January 2018 to December 2025. The primary center served as the development cohort, and the external validation center served as the independent validation cohort.

### 2.3 MRI acquisition and image assessment

Preoperative liver MRI was performed with identical acquisition parameters at both centers using a Siemens Skyra 3.0-T MRI scanner. Patients fasted for 4–6 hours before examination and were scanned in the supine, head-first position. Regular breathing was practiced before scanning, and end-expiratory breath-holding was used when required. The protocol included T1-weighted imaging (T1WI), fat-suppressed T2-weighted imaging (FS-T2WI), diffusion-weighted imaging (DWI), and dynamic contrast-enhanced imaging. Dynamic contrast-enhanced MRI was performed after antecubital venous injection of the extracellular gadolinium-based contrast agent gadopentetate dimeglumine (Gd-DTPA; Magnevist, Bayer) at a dose of 0.1 mmol/kg and an injection rate of 2–3 mL/s, followed by a 20-mL saline flush. Arterial phase (AP) and portal venous phase (PVP) images were acquired approximately 20–35 s and 60–70 s after the start of contrast injection, respectively. Key acquisition parameters were as follows: for T1WI, TR/TE was 3.89/1.23 ms, section thickness/gap was 3/0.60 mm, FOV was 400 x 325 mm, matrix was 176 x 288, and flip angle was 10 degrees; for FS-T2WI, TR/TE was 1720/87 ms, section thickness/gap was 5/0.50 mm, FOV was 380 x 380 mm, matrix was 320 x 320, and flip angle was 99 degrees; for DWI, TR/TE was 4300/64 ms, section thickness/gap was 5/1.00 mm, FOV was 380 x 304 mm, matrix was 128 x 128, flip angle was 90 degrees, and b values were 0, 50, and 800 s/mm2; for AP, TR/TE was 3.89/1.23 ms, section thickness/gap was 3/0.60 mm, FOV was 400 x 325 mm, matrix was 176 x 288, and flip angle was 10 degrees; and for PVP, TR/TE was 3.31/1.30 ms, section thickness/gap was 3/0.60 mm, FOV was 380 x 308 mm, matrix was 195 x 320, and flip angle was 9 degrees. Detailed MRI acquisition parameters are provided in **Supplementary Table S10**, and MRI feature definitions and coding rules are provided in **Supplementary Table S11**.

MRI features were independently assessed by three abdominal radiologists, each with more than 3 years of experience in abdominal imaging. The readers were blinded to postoperative recurrence outcomes, pathologic results, and laboratory data to reduce information bias in predictor assessment. Disagreements were resolved by consensus discussion, and the consensus results were used for modeling. To evaluate reading reliability, 60 randomly selected patients were reassessed by the three radiologists under independent blinded conditions, and interreader agreement was calculated (16). Detailed interreader agreement results are provided in **Supplementary Table S2**.

### 2.4 Follow-up and outcome

The primary outcome was early recurrence, defined as recurrence within 24 months after curative hepatectomy (4–6); recurrence after 24 months was considered late recurrence. Postoperative recurrence was defined as a new intrahepatic HCC lesion and/or extrahepatic metastasis detected during follow-up by dynamic contrast-enhanced CT, MRI, or pathologic examination after curative hepatectomy. Intrahepatic recurrence was mainly diagnosed according to typical HCC imaging features, including arterial phase hyperenhancement, washout on portal venous or delayed phase images, capsule-like enhancement, or progressive lesion enlargement. Serum AFP elevation was used only as an auxiliary indicator of possible recurrence and was not used alone to diagnose recurrence (2, 3). Recurrence time was defined as the interval from surgery to the first imaging or pathologic confirmation of recurrence. Follow-up imaging was independently and blindly assessed by three abdominal radiologists, and disagreements were resolved by discussion. Follow-up was scheduled monthly during the first postoperative year, every 3 months during the second postoperative year, and every 3–6 months thereafter. Each follow-up assessment integrated contrast-enhanced CT or MRI, serum AFP, and other laboratory tests as clinically indicated. The last follow-up date was April 1, 2026. Disease-free survival (DFS) started on the date of curative hepatectomy and ended on the date of first confirmed recurrence. For 24-month Cox modeling, follow-up time was truncated at 24 months. Patients without confirmed recurrence within 24 months were censored at the last recurrence-free follow-up date or at 24 months, whichever occurred first. For patients operated in 2024-2025, confirmed recurrence before the data cutoff was counted as early recurrence only when it occurred within 24 months after surgery; patients who had not yet reached 24 months and had no confirmed recurrence at the data cutoff were right-censored at the last recurrence-free follow-up and were not classified as having completed 24-month recurrence-free observation. Censoring scenarios included follow-up shorter than 24 months without recurrence, death within 24 months without confirmed recurrence before death, loss to follow-up within 24 months without confirmed recurrence, and remaining recurrence-free at 24 months. If definite residual active disease was detected on baseline imaging within 1 month after surgery, the patient was considered to have residual disease or noncurative resection and was not counted as an early recurrence event.

### 2.5 Predictors and data processing

Candidate predictors included preoperative laboratory indices and MRI features selected according to previous HCC recurrence prediction studies and clinical interpretability (5, 7–10). Laboratory indices were collected within 1 week before surgery. MRI variables were uniformly coded as binary variables, with 1 indicating presence and 0 indicating absence. Tumor size was analyzed as a binary variable using a cutoff of >5 cm. For multifocal tumors, the largest tumor diameter was used. Tumor number was recorded in the source imaging forms but was not entered into the candidate predictor pool by design. This decision was made before modeling to avoid mixing tumor-count information with the MRI feature of satellite nodule and to reduce ambiguity between separate small tumors and satellite lesions on preoperative MRI. Multifocality was therefore not considered clinically unimportant; rather, its related imaging information was partly represented through largest tumor diameter and satellite nodule status in this preoperative model. Postoperative adjuvant therapy was coded as received or not received but was not included in the main preoperative prediction model. It was used only in sensitivity analyses. Skewed biomarkers, including PIVKA-II and NLR, were natural log-transformed when appropriate. After data recollection, no missing values remained in the final modeling variables.

### 2.6 Statistical analysis

Baseline characteristics were described for all patients, the development cohort, and the external validation cohort. The Shapiro-Wilk test was used to assess normality of continuous variables (17), and distribution assessment results are provided in **Supplementary Table S1**. Continuous variables are reported as means with standard deviations or medians with interquartile ranges, as appropriate. Categorical variables are reported as numbers and percentages. Between-cohort comparisons used the Student t test (19) or Mann-Whitney U test (18) for continuous variables and the chi-square test (20) or Fisher exact test (21) for categorical variables, as appropriate. Differences between patients with and without early recurrence in the development cohort were assessed using the same approach. **Table 1** reports a condensed set of these development-cohort comparisons, whereas the complete early-versus-non-early recurrence comparison is provided in **Supplementary Table S16**.

**Table 1:** Main characteristics of patients with and without early recurrence in the development cohort.

Characteristic	Non-early recurrence (n = 105)	Early recurrence (n = 153)	P value
Clinical and Laboratory Characteristics			
log(CEA)*	1.02 (0.68-1.39)	1.26 (0.85-2.92)	<.001
log(CA19-9)*	2.79 (2.33-3.08)	3.09 (2.40-5.92)	<.001
log(AFP)*	2.97 (1.70-5.65)	4.82 (1.82-7.91)	.011
AFP >400, yes	24 (22.9)	59 (38.6)	.008
log(PIVKA-II)*	4.40 (2.06-5.83)	6.67 (5.04-9.20)	<.001
AST >40, yes	42 (40.0)	79 (51.6)	.066
log(ALP)*	4.52 (4.32-4.80)	4.70 (4.36-4.93)	.039
log(GGT)*	3.74 (3.37-4.47)	4.25 (3.66-4.96)	<.001
Platelets*	139 (93-187)	156 (114-203)	.024
log(NLR)*	1.29 (1.01-1.57)	1.31 (1.06-1.50)	.790
Total bilirubin*	17.0 (13.1-20.7)	15.0 (11.6-20.4)	.075
AST/ALT ratio*	1.05 (0.79-1.33)	1.27 (0.93-1.66)	.001
ALBI grade 2 or 3, yes	28 (26.7)	61 (39.9)	.028
Preoperative MRI Characteristics			
Tumor size >5 cm, yes	26 (24.8)	92 (60.1)	<.001
Satellite nodule, yes	6 (5.7)	38 (24.8)	<.001
Infiltrative appearance, yes	18 (17.1)	69 (45.1)	<.001
Intrahepatic vessel invasion, yes	6 (5.7)	33 (21.6)	<.001
Peritumoral hypointensity on portal venous phase images, yes	5 (4.8)	51 (33.3)	<.001
Incomplete capsule, yes	54 (51.4)	117 (76.5)	<.001
Nodule-in-nodule architecture, yes	11 (10.5)	54 (35.3)	<.001
Multiseptated or multinodular architecture, yes	29 (27.6)	87 (56.9)	<.001
Necrosis or severe ischemia, yes	38 (36.2)	81 (52.9)	.008
Intratumoral artery, yes	17 (16.2)	53 (34.6)	.001
Capsule present, yes	89 (84.8)	107 (69.9)	.006
Capsule enhancement, yes	87 (82.9)	106 (69.3)	.014
Ring enhancement, yes	21 (20.0)	47 (30.7)	.055
Intratumoral hemorrhage, yes	34 (32.4)	72 (47.1)	.019
Peritumoral T2 hyperintensity, yes	14 (13.3)	61 (39.9)	<.001
APHE proportion <50%, yes	25 (23.8)	61 (39.9)	.007

This condensed main-text table retains variables entered into the candidate pool, final model variables, clinically important variables, and variables with relatively apparent differences in univariable comparisons. The complete variable list is provided in **Supplementary Table S16**. Unless otherwise indicated, categorical variables are reported as numbers with percentages, and continuous variables are reported as medians with interquartile ranges. P values compare patients with and without early recurrence in the development cohort. Categorical variables were compared using the chi-square test or Fisher exact test, as appropriate, and continuous variables were compared using the Mann-Whitney U test. This table describes group differences and univariable comparisons and does not identify independent predictors.

* indicates continuous variables reported as medians with interquartile ranges.

In the development cohort, each candidate variable was first evaluated using univariable Cox regression. Univariable logistic regression, with binary early recurrence status as the dependent variable, was used as a screening analysis. Variables with univariable P < 0.10 in either analysis were considered for candidate selection. Variables considered clinically important within the predefined candidate-predictor framework were also reviewed for inclusion even when they did not meet this screening threshold. Candidate selection, premodel variable pool construction, and collinearity assessment are provided in **Supplementary Tables S3**-**S7**; the variables shown in **Table 1** should therefore be read as descriptive and univariable comparison variables, not as the complete independent-predictor set.

Because recurrence time and censoring information were available, Cox proportional hazards regression was selected as the primary modeling method (22). Clinical, MRI, and combined models were developed separately in the development cohort. The combined model included recommended clinical and MRI variables and used backward selection based on the Akaike information criterion to obtain a more parsimonious final model. The proportional hazards assumption was assessed using Schoenfeld residuals (23). Agreement for categorical MRI features was evaluated using Fleiss kappa (16) strictly in the 60-patient reassessment subset, rather than in the full cohort, with approximate 95% confidence intervals estimated from 1000 bootstrap resamples. Detailed results for the clinical and MRI models are provided in **Supplementary Table S8**.

Internal validation was performed using bootstrap resampling (24). A 200-resample bootstrap run was first used to test the workflow, followed by formal internal validation with 1000 bootstrap resamples. The final model was applied to the external validation cohort by using the fixed regression coefficients and baseline survival estimated in the development cohort, without refitting. Discrimination was evaluated using the C-index and 24-month time-dependent AUC (25). Overall prediction error was evaluated using the 24-month Brier score. Calibration was evaluated using calibration curves, calibration slope, and calibration intercept (26). Confidence intervals for internal validation metrics were derived from bootstrap distributions. Confidence intervals for external validation metrics were estimated by patient-level bootstrap resampling with the development model held fixed. Decision curve analysis (DCA) was used to evaluate clinical net benefit across threshold probabilities (27). The primary performance metrics with 95% confidence intervals are provided in **Supplementary Table S14**, and the TRIPOD checklist is provided in **Supplementary Table S13**.

Postoperative adjuvant therapy was evaluated in sensitivity analyses and was not included in the main preoperative model. Sensitivity analyses included adding adjuvant therapy as an additional covariate to the final model, stratified analyses according to adjuvant therapy status, and descriptive comparison of model performance across adjuvant therapy subgroups. Detailed sensitivity analysis results are provided in **Supplementary Table S9**. Risk stratification was based on the final combined model and used a cutoff of 0.584, determined by the Youden index in the development cohort. This cutoff was considered exploratory and was used for risk communication and research stratification rather than as a standalone treatment-decision threshold. All statistical analyses were performed using R software version 4.5.2 with the survival, rms, timeROC, pROC, rmda, boot, irr, psych, readxl, writexl, flextable, officer, dplyr, ggplot2, survminer, and splines packages.

## 3 Results

### 3.1 Patient inclusion and cohort characteristics

A total of 445 patients were initially screened at the two centers, and 94 were excluded because of previous HCC treatment, non-HCC malignancy, ruptured HCC, concomitant interventional therapy during surgery, or insufficient MRI image quality. After these exclusions, 351 patients were eligible for postoperative follow-up assessment, and 31 were further excluded because follow-up information was unavailable. The final analysis cohort included 320 patients, consisting of 258 in the development cohort and 62 in the external validation cohort. Overall, 183 patients (57.2%) experienced early recurrence, including 153 of 258 patients (59.3%) in the development cohort and 30 of 62 patients (48.4%) in the external validation cohort. Among the 137 patients without early recurrence, the median follow-up duration was 35.6 months (IQR, 28.5-47.6), including 36.5 months (IQR, 28.6-48.3) in the development cohort and 32.3 months (IQR, 28.4-39.2) in the external validation cohort. No patient classified as without early recurrence had less than 24 months of follow-up in the final analysis dataset; the minimum follow-up duration among these patients was 24.1 months overall and 24.2 months in the external validation cohort.

### 3.2 Baseline differences between the development and external validation cohorts

Several clinical characteristics differed between the development and external validation cohorts. Chronic hepatitis B was more frequent in the development cohort than in the external validation cohort (76.0% vs 50.0%, P < 0.001). The development cohort had higher log(AFP) (P = 0.036), whereas the external validation cohort had higher NLR and log(NLR) (both P = 0.034). Postoperative adjuvant therapy also differed between cohorts and was more frequent in the external validation cohort than in the development cohort (83.9% vs 50.8%, P < 0.001). MRI features retained in the final model were broadly comparable between cohorts: tumor size >5 cm (45.7% vs 48.4%, P = 0.707), satellite nodule (17.1% vs 12.9%, P = 0.426), intrahepatic vessel invasion (15.1% vs 11.3%, P = 0.441), peritumoral hypointensity on portal venous phase images (21.7% vs 24.2%, P = 0.672), and incomplete capsule (66.3% vs 75.8%, P = 0.148). Baseline characteristics of the development and external validation cohorts are summarized in **Table 2**.

**Table 2:** Main baseline characteristics of the development and external validation cohorts.

Characteristic	All patients (n = 320)	Development cohort (n = 258)	External validation cohort (n = 62)	P value
Clinical and Laboratory Characteristics				
Chronic hepatitis B, yes	227 (70.9)	196 (76.0)	31 (50.0)	<.001
log(AFP)*	3.57 (1.73-6.84)	3.69 (1.79-7.06)	2.70 (1.56-5.65)	.036
AFP >400, yes	98 (30.6)	83 (32.2)	15 (24.2)	.221
log(PIVKA-II)*	5.58 (3.45-8.25)	5.61 (3.77-7.84)	4.19 (1.64-9.35)	.121
Prothrombin time*	13.5 (12.5-14.8)	13.3 (12.5-14.2)	16.9 (15.3-18.1)	<.001
NLR*	2.68 (1.82-3.82)	2.66 (1.77-3.71)	3.22 (2.19-5.10)	.034
log(NLR)*	1.30 (1.04-1.57)	1.30 (1.02-1.55)	1.44 (1.16-1.81)	.034
HBsAb positive, yes	59 (18.4)	36 (14.0)	23 (37.1)	<.001
HBeAg positive, yes	33 (10.3)	22 (8.5)	11 (17.7)	.032
AST/ALT ratio*	1.16 (0.84-1.52)	1.18 (0.90-1.56)	1.07 (0.65-1.39)	.010
ALBI grade 2 or 3, yes	106 (33.1)	89 (34.5)	17 (27.4)	.288
Postoperative adjuvant therapy, yes	183 (57.2)	131 (50.8)	52 (83.9)	<.001
Preoperative MRI Characteristics				
Tumor size >5 cm, yes	148 (46.2)	118 (45.7)	30 (48.4)	.707
Satellite nodule, yes	52 (16.2)	44 (17.1)	8 (12.9)	.426
Intrahepatic vessel invasion, yes	46 (14.4)	39 (15.1)	7 (11.3)	.441
Peritumoral hypointensity on portal venous phase images, yes	71 (22.2)	56 (21.7)	15 (24.2)	.672
Rim arterial phase hyperenhancement, yes	212 (66.2)	177 (68.6)	35 (56.5)	.069
Incomplete capsule, yes	218 (68.1)	171 (66.3)	47 (75.8)	.148
Nodule-in-nodule architecture, yes	73 (22.8)	65 (25.2)	8 (12.9)	.038
Necrosis or severe ischemia, yes	156 (48.8)	119 (46.1)	37 (59.7)	.055
Intratumoral artery, yes	102 (31.9)	70 (27.1)	32 (51.6)	<.001
Intratumoral fat, yes	196 (61.3)	170 (65.9)	26 (41.9)	<.001
Intratumoral iron deficiency or internal nodule, yes	268 (83.8)	222 (86.0)	46 (74.2)	.023
Blood-pool enhancement pattern, yes	24 (7.5)	23 (8.9)	1 (1.6)	.058
APHE proportion <50%, yes	107 (33.4)	86 (33.3)	21 (33.9)	.936
Ascites, yes	42 (13.1)	24 (9.3)	18 (29.0)	<.001
Imaging cirrhosis, yes	138 (43.1)	109 (42.2)	29 (46.8)	.518
Diffuse iron overload, yes	44 (13.8)	17 (6.6)	27 (43.5)	<.001
Diffuse hepatic steatosis, yes	98 (30.6)	94 (36.4)	4 (6.5)	<.001
Main portal vein width (cm)*	1.3 (1.1-1.4)	1.3 (1.1-1.4)	1.4 (1.3-1.5)	<.001
Portosystemic shunt/collaterals, yes	25 (7.8)	25 (9.7)	0 (0.0)	.007
Varices, yes	32 (10.0)	30 (11.7)	2 (3.2)	.047
Follow-up and Endpoint				
Disease-free survival (months)*	18.9 (9.7-32.5)	17.1 (8.8-32.4)	24.9 (13.1-32.4)	.086
Early recurrence (<24 months), yes	183 (57.2)	153 (59.3)	30 (48.4)	.119

This condensed main-text table retains core clinical variables, final model variables, clinically important MRI variables, and variables with relatively apparent between-cohort differences. The complete variable list is provided in **Supplementary Table S15**. Unless otherwise indicated, categorical variables are reported as numbers with percentages, and continuous variables are reported as medians with interquartile ranges. P values compare the development and external validation cohorts. Categorical variables were compared using the chi-square test or Fisher exact test, as appropriate, and continuous variables were compared using the Mann-Whitney U test.

* indicates continuous variables reported as medians with interquartile ranges.

### 3.3 Comparison of patients with and without early recurrence in the development cohort

In the development cohort, patients with early recurrence had higher log(PIVKA-II) than those without early recurrence (median, 6.67 vs 4.40; P < 0.001), and AFP >400 ng/mL was more frequent in patients with early recurrence (38.6% vs 22.9%, P = 0.008). Early recurrence was also more common among patients with tumor size >5 cm (60.1% vs 24.8%, P < 0.001), satellite nodule (24.8% vs 5.7%, P < 0.001), intrahepatic vessel invasion (21.6% vs 5.7%, P < 0.001), peritumoral hypointensity on portal venous phase images (33.3% vs 4.8%, P < 0.001), and incomplete capsule (76.5% vs 51.4%, P < 0.001). The main comparisons between patients with and without early recurrence in the development cohort are summarized in **Table 1**.

### 3.4 Interreader agreement

In the 60-patient reassessment subset, agreement for MRI features retained in the final model was moderate to high. Fleiss kappa values were 0.775 for tumor size >5 cm, 0.572 for satellite nodule, 0.652 for intrahepatic vessel invasion, 0.743 for peritumoral hypointensity on portal venous phase images, and 0.766 for incomplete capsule. Complete agreement among the three readers for these features ranged from 76.7% to 86.7%. These values reflect reproducibility in the reassessed subset rather than agreement estimates from the full cohort.

### 3.5 Final combined model

Backward AIC selection yielded a final combined Cox model with six predictors: log(PIVKA-II), tumor size >5 cm, satellite nodule, peritumoral hypointensity on portal venous phase images, intrahepatic vessel invasion, and incomplete capsule. In the final model, log(PIVKA-II) was associated with early recurrence (HR, 1.23; 95% CI: 1.14-1.31; P < 0.001). MRI predictors included tumor size >5 cm (HR, 1.42; 95% CI: 0.97-2.08; P = 0.067), satellite nodule (HR, 2.33; 95% CI: 1.55-3.49; P < 0.001), peritumoral hypointensity on portal venous phase images (HR, 1.83; 95% CI: 1.28-2.63; P = 0.001), intrahepatic vessel invasion (HR, 1.76; 95% CI: 1.14-2.72; P = 0.011), and incomplete capsule (HR, 1.42; 95% CI: 0.95-2.12; P = 0.085). The apparent C-index of the final model in the development cohort was 0.756, and the global proportional hazards assumption was not violated (global P = 0.187). Final model estimates are summarized in **Table 3**, and the corresponding nomogram is shown in **Figure 2**.

**Table 3:** Final combined Cox model for predicting early recurrence.

Variable	Domain	Beta	HR (95% CI)	P value
log(PIVKA-II)	Clinical	0.204	1.23 (1.14-1.31)	<.001
Tumor size >5 cm	MRI	0.354	1.42 (0.97-2.08)	.067
Satellite nodule	MRI	0.846	2.33 (1.55-3.49)	<.001
Peritumoral hypointensity on portal venous phase images	MRI	0.605	1.83 (1.28-2.63)	.001
Intrahepatic vessel invasion	MRI	0.565	1.76 (1.14-2.72)	.011
Incomplete capsule	MRI	0.352	1.42 (0.95-2.12)	.085

This table lists only the predictors retained in the final combined Cox model. The linear prediction formula is provided in the main text.

**Figure 2:**
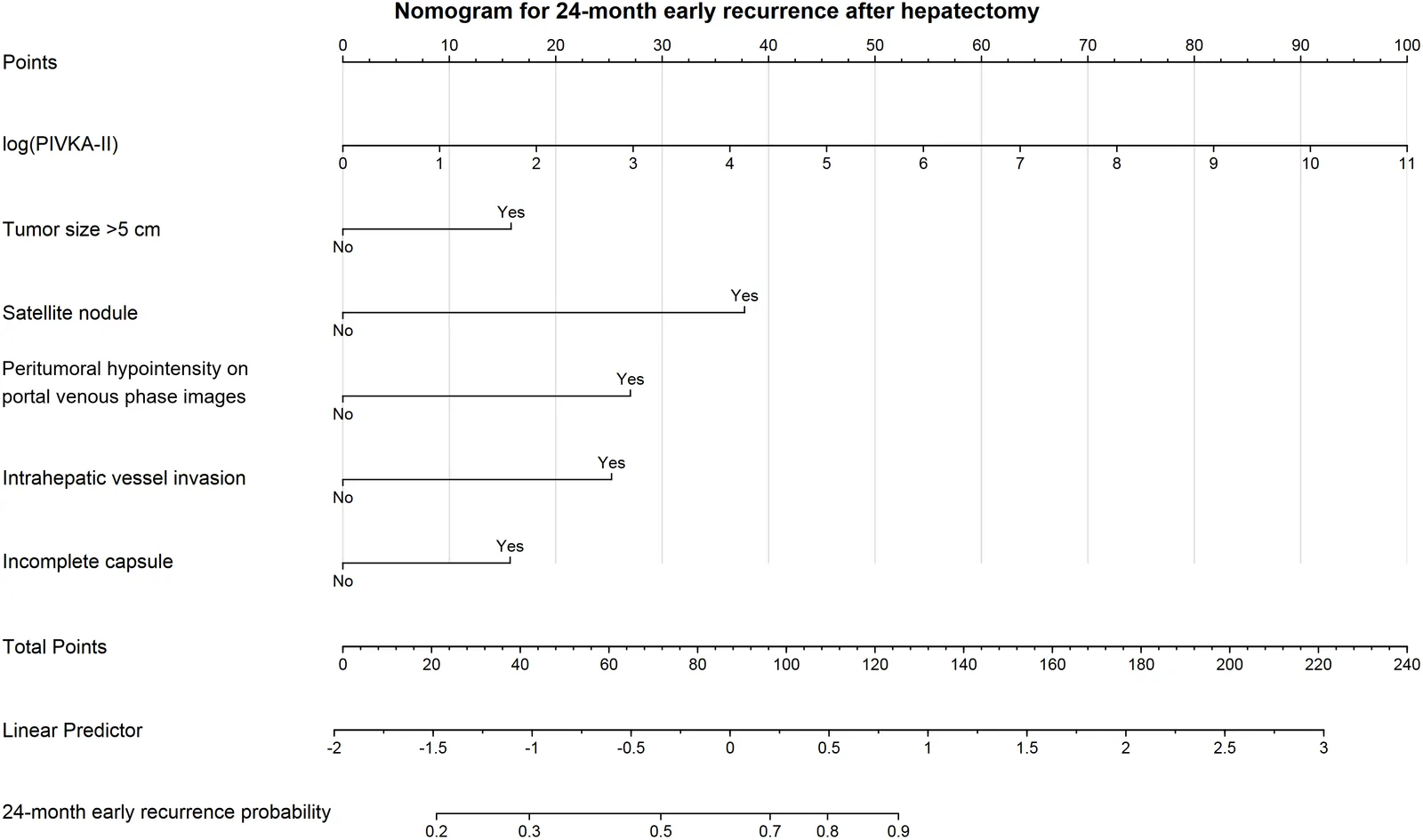
Nomogram of the final combined model. The nomogram was derived from the final combined Cox model developed in the development cohort and was designed to predict 24-month early recurrence after surgery. The model includes log(PIVKA-II), tumor size >5 cm, satellite nodule, peritumoral hypointensity on portal venous phase images, intrahepatic vessel invasion, and incomplete capsule. Each predictor corresponds to a point score, and the total point score is converted to the predicted probability of 24-month early recurrence. This nomogram is intended for preoperative risk estimation and risk communication and should not be used as a standalone basis for treatment decisions.

Using the regression coefficients of the final combined model and the 24-month baseline recurrence-free survival in the development cohort (S0(24) = 0.870202), a static web-based risk calculator was created and made available as both an online tool (https://clarrmy.github.io/hcc-early-recurrence-calculator/) and an offline package. The calculator accepts PIVKA-II and five MRI features as inputs, estimates the predicted probability of early recurrence within 24 months after surgery, and classifies patients as high or low risk according to the prespecified cutoff of 0.584. All calculations are performed locally in the browser, and no patient information is uploaded, stored, or transmitted. The tool is intended only for research-oriented preoperative risk estimation and risk communication and should not replace multidisciplinary clinical judgment or serve as a standalone basis for treatment decisions.

### 3.6 Internal and external validation

In 1000 bootstrap resamples for internal validation, the optimism-corrected 24-month AUC was 0.829 (0.814 to 0.843), the Brier score was 0.169 (0.159 to 0.175), the calibration slope was 0.956 (0.719 to 1.217), and the calibration intercept was -0.014 (-0.297 to 0.287). In the external validation cohort, the model had a C-index of 0.892 (0.841 to 0.937), a 24-month AUC of 0.978 (0.945 to 1.000), and a Brier score of 0.087 (0.060 to 0.120). The external calibration slope was 2.705 (1.882 to 46.376), and the calibration intercept was -1.288 (-12.850 to -0.067), indicating imperfect absolute risk calibration despite high discrimination. The negative intercept and the grouped calibration points below the ideal line in the lower predicted-risk range indicate systematic overestimation of absolute risk in this range, whereas a slope greater than 1 suggests that the predicted-risk range was compressed relative to the observed outcome gradient. Because each external grouped-calibration stratum contained only 15 to 16 patients, these points were interpreted descriptively rather than as stable subgroup-specific calibration estimates. From the lowest to highest predicted-risk strata, the mean predicted/observed 24-month risks were 0.220/0.000 (n = 16), 0.375/0.133 (n = 15), 0.753/0.800 (n = 15), and 0.945/1.000 (n = 16), respectively. Model performance metrics are summarized in **Table 4**, and discrimination and calibration are shown in **Figure 3** and **Figure 4**, respectively.

**Table 4:** Prediction performance of the final combined model in development, internal validation, and external validation settings.

Validation setting/cohort	N	Events	C-index (95% CI)	24-month AUC (95% CI)	24-month Brier score (95% CI)	Calibration slope (95% CI)	Calibration intercept (95% CI)
Development apparent performance	258	153	0.756 (0.715 to 0.796)	0.838 (0.789 to 0.882)	0.163 (0.141 to 0.185)	1.060 (0.755 to 1.533)	-0.016 (-0.309 to 0.276)
Bootstrap-corrected internal validation	258	153	0.749 (0.710 to 0.792)	0.829 (0.814 to 0.843)	0.169 (0.159 to 0.175)	0.956 (0.719 to 1.217)	-0.014 (-0.297 to 0.287)
External validation cohort	62	30	0.892 (0.841 to 0.937)	0.978 (0.945 to 1.000)	0.087 (0.060 to 0.120)	2.705 (1.882 to 46.376)	-1.288 (-12.850 to -0.067)

Internal validation used 1000 bootstrap resamples. External validation used the fixed development cohort model without refitting. Values in parentheses are 95% confidence intervals. A lower Brier score indicates smaller overall prediction error.

**Figure 3:**
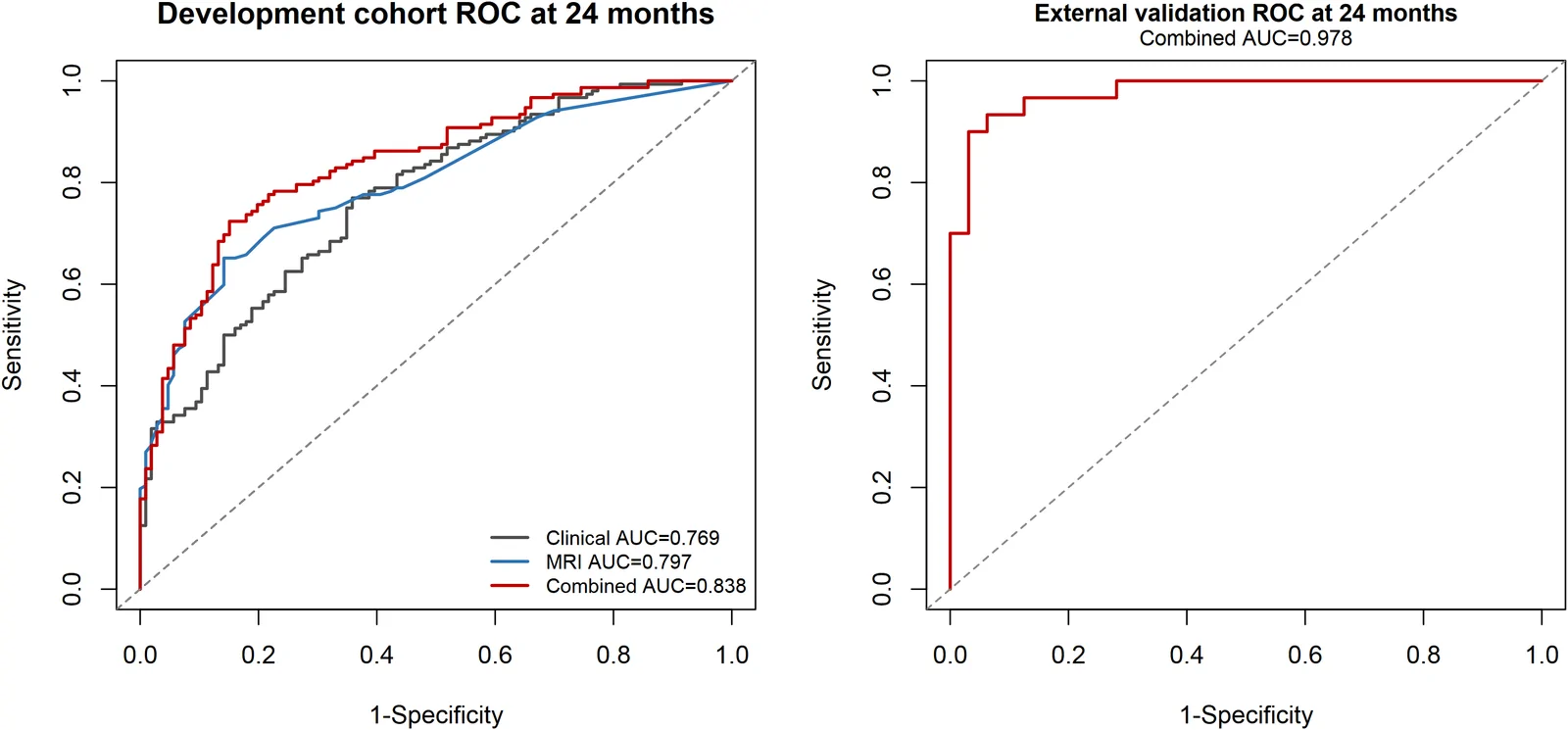
Discrimination curves. The figure shows 24-month time-dependent receiver operating characteristic curves comparing the clinical model, MRI model, and final combined model in the development cohort, together with the performance of the final combined model in the external validation cohort. The curves reflect the ability of the models to rank early recurrence risk but do not evaluate the accuracy of individual absolute risk prediction. In the external validation cohort, the combined model showed high discrimination, with a 24-month AUC of 0.978 (95% CI: 0.945-1.000), indicating strong risk ranking in this cohort. However, given the limited size of the external validation cohort and the observed overestimation of risk in the low predicted-risk range on calibration analysis, the external validation results should be interpreted together with the calibration curves and Brier score.

**Figure 4:**
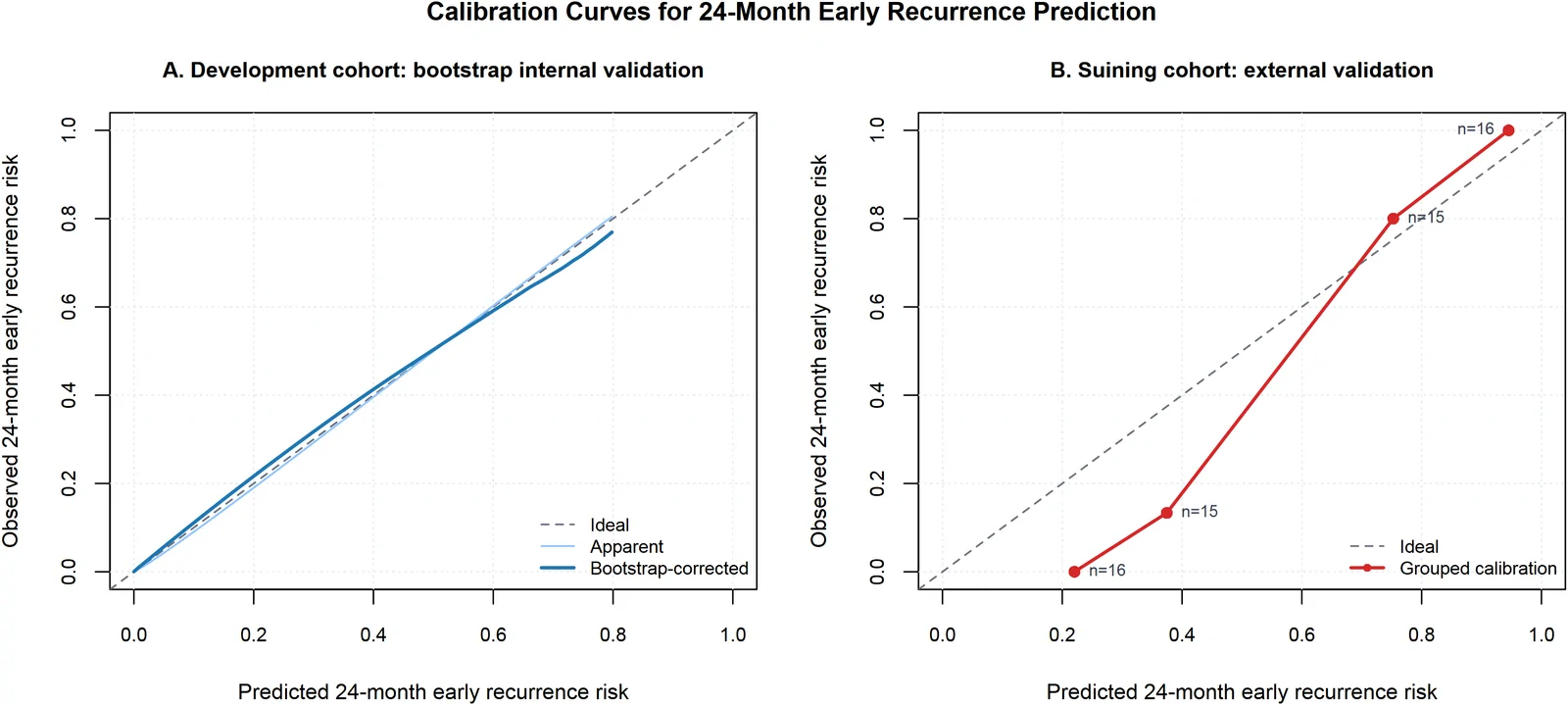
Twenty-four-month calibration curves. Calibration curves compare the predicted probability of 24-month early recurrence from the final combined model with observed risk in bootstrap internal validation of the development cohort and in the external validation cohort. Agreement between predicted and observed risk indicates better calibration. In the external validation cohort, grouped calibration points were based on 15 to 16 patients per stratum and showed risk overestimation in the lower predicted-risk range. These grouped points were used to describe calibration direction and should not be interpreted as precise stratum-specific risk estimates.

### 3.7 Comparison of the clinical, MRI, and combined models

In the development cohort, the combined model showed higher discrimination and lower prediction error than the clinical and MRI models alone. The 24-month AUCs of the clinical, MRI, and combined models were 0.769, 0.797, and 0.838, respectively, and the corresponding Brier scores were 0.191, 0.181, and 0.163. Paired bootstrap comparison of binary 24-month AUC showed that the combined model outperformed the clinical model (difference, 0.066; 95% CI: 0.023-0.108; P = 0.004) and the MRI model (difference, 0.045; 95% CI: 0.011-0.082; P = 0.008). In the external validation cohort, the combined model had the highest C-index and AUC, but its Brier score was slightly higher than that of the clinical model. Therefore, external validation performance should not be interpreted on the basis of discrimination alone. The clinical, MRI, and combined model comparison is summarized in **Table 5**.

**Table 5:** Comparison of the clinical, MRI, and combined models.

Cohort	Model	N	Events	C-index	24-month AUC	24-month Brier score	DCA useful threshold range
Development cohort	Clinical model	258	153	0.710	0.769	0.191	0.17-0.80
Development cohort	MRI model	258	153	0.716	0.797	0.181	0.28-0.80
Development cohort	Combined model	258	153	0.756	0.838	0.163	0.15-0.80
External validation cohort	Clinical model	62	30	0.886	0.973	0.079	0.16-0.80
External validation cohort	MRI model	62	30	0.703	0.764	0.219	0.28-0.72
External validation cohort	Combined model	62	30	0.892	0.978	0.087	0.18-0.80

In the development cohort, the combined model had the best discrimination and Brier score. In external validation, the combined model had the highest C-index and AUC, but the clinical model had a slightly lower Brier score.

### 3.8 Decision curve analysis and risk stratification

Decision curve analysis showed that the final combined model provided positive net benefit compared with treat-all and treat-none strategies across threshold probabilities of 0.15-0.80 in the development cohort and 0.18-0.80 in the external validation cohort. Using the Youden index in the development cohort, a predicted probability of 0.584 was selected as the exploratory cutoff for risk stratification. In the development cohort, early recurrence occurred in 110 of 126 high-risk patients (87.3%) and 43 of 132 low-risk patients (32.6%). In the external validation cohort, early recurrence occurred in 29 of 33 high-risk patients (87.9%) and one of 29 low-risk patients (3.4%). High-risk classification was associated with shorter DFS in the development cohort (HR, 5.448; 95% CI: 3.790-7.831; P < 0.001) and the external validation cohort (HR, 60.785; 95% CI: 8.172-452.142; P < 0.001). The very large external HR primarily reflected the single early recurrence event in the low-risk reference group and should be interpreted as evidence of directional risk separation rather than as a stable estimate of clinical effect magnitude. Decision curve analysis and risk stratification are shown in **Figure 5** and **Figure 6**, respectively, and risk-stratified outcomes are summarized in **Table 6**. A representative preoperative MRI case illustrating the model predictors and risk estimation is shown in **Figure 7**.

**Figure 5:**
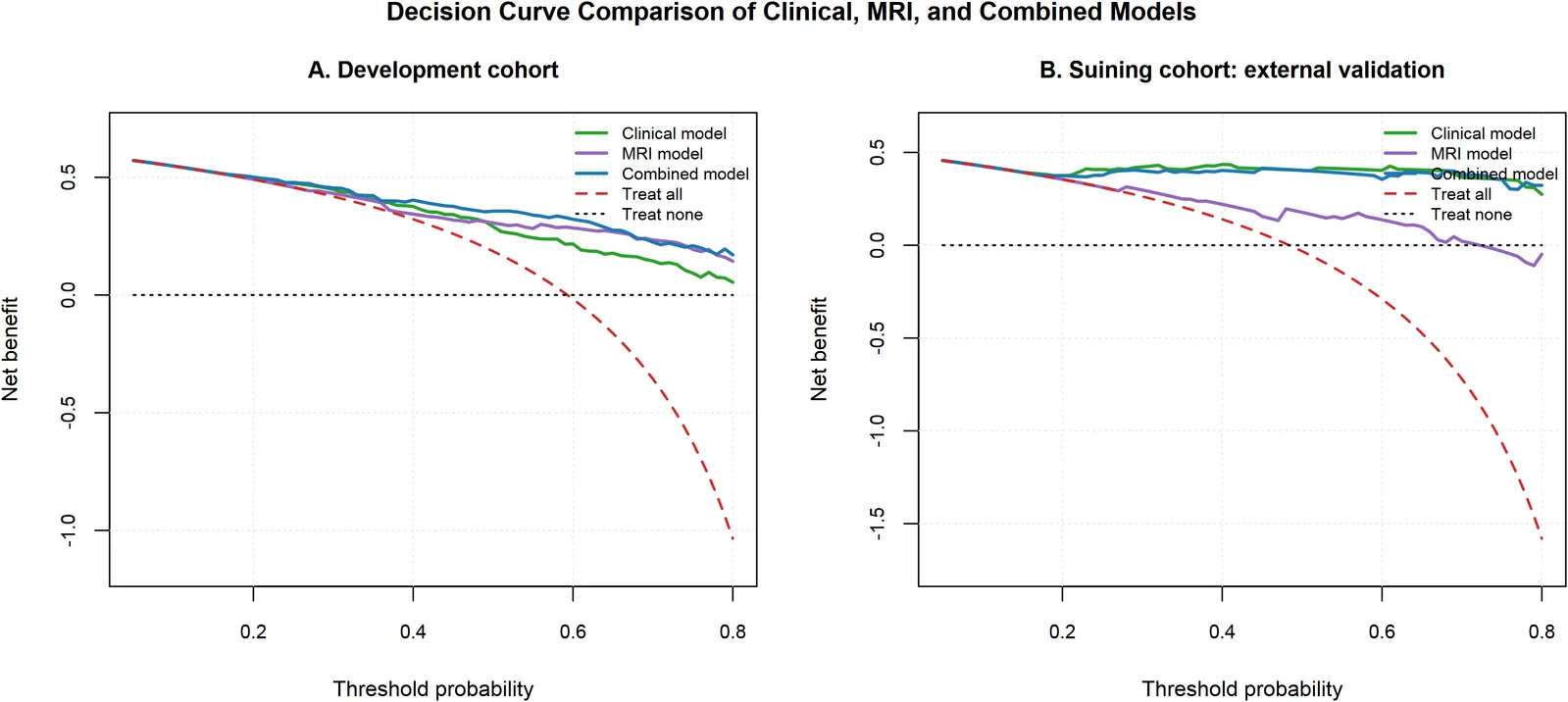
Decision curve comparison of the clinical, MRI, and combined models. Decision curve analysis compares the net benefit of the clinical model, MRI model, and final combined model across different threshold probabilities, with treat-all and treat-none strategies as references. The analysis was performed for 24-month early recurrence, with threshold probabilities ranging from 0.05 to 0.80. DCA evaluates potential clinical net benefit across risk thresholds but does not prove that model-guided treatment improves patient outcomes.

**Figure 6:**
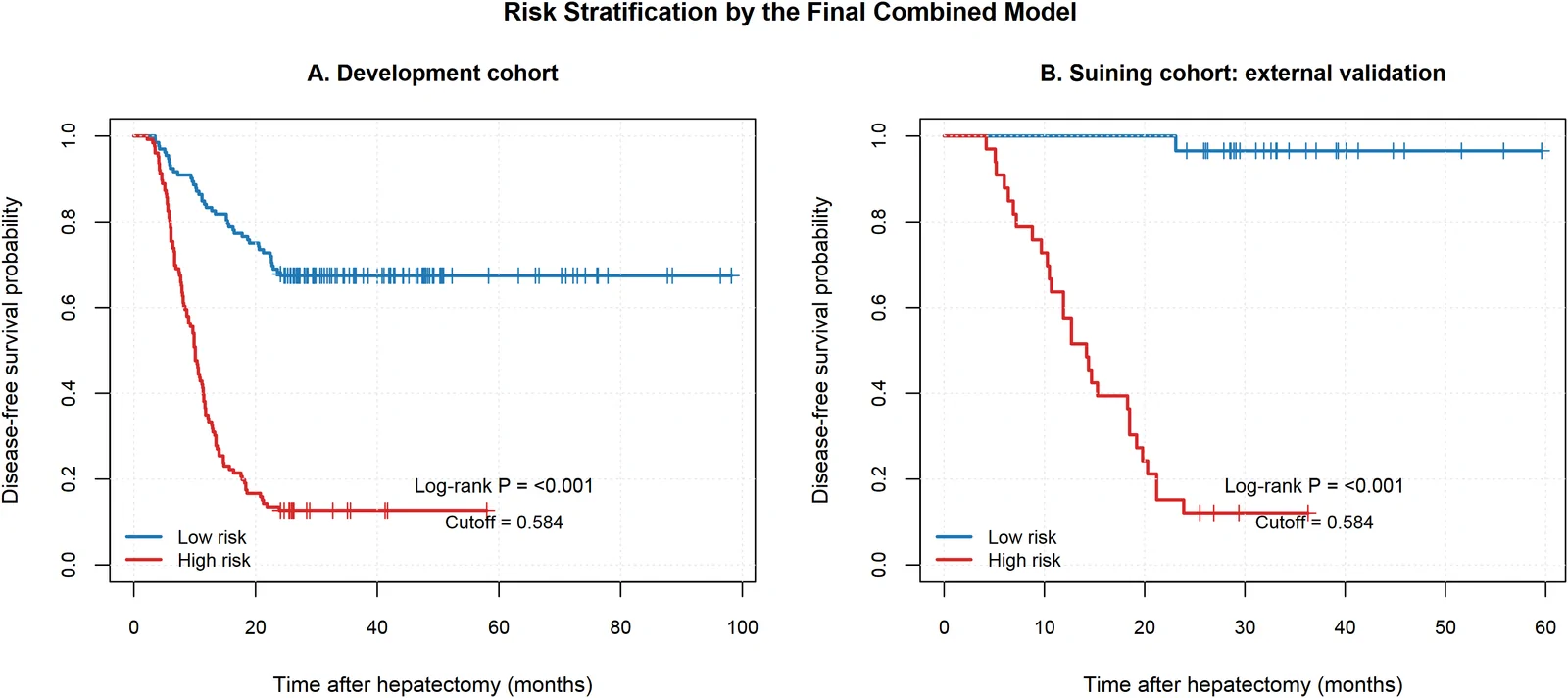
Risk stratification by the final combined model. Kaplan-Meier curves show differences in disease-free survival after risk stratification based on predicted probabilities from the final combined model. The cutoff of 0.584 was derived from the development cohort and then applied without recalibration to the external validation cohort. P values were calculated using the log-rank test. In the external validation cohort, the very large HR was driven by only one early recurrence event in the low-risk reference group and should be interpreted as directional evidence of group separation rather than as a precise effect-size estimate.

**Table 6:** Risk stratification cutoff and outcomes.

Cohort	Risk group	N	Early recurrence, n (%)	Median predicted risk	HR (95% CI)	Cox p value	Log-rank p value	Group comparison p value
Development cohort	Low risk	132	43 (32.6%)	0.350	Reference		<0.001	<0.001
Development cohort	High risk	126	110 (87.3%)	0.847	5.448 (3.790-7.831)	<0.001	<0.001	<0.001
External validation cohort	Low risk	29	1 (3.4%)	0.258	Reference		<0.001	<0.001
External validation cohort	High risk	33	29 (87.9%)	0.844	60.785 (8.172-452.142)	<0.001	<0.001	<0.001

High risk was defined as a predicted probability of 24-month early recurrence >=0.584, determined by the Youden index in the development cohort. Sensitivity was 0.719, specificity was 0.848, and the Youden index was 0.566 in the development cohort. The cutoff was exploratory and intended for risk communication and research stratification rather than standalone treatment decision-making. In the external validation cohort, the HR had a wide confidence interval because only one early recurrence event occurred in the low-risk group.

**Figure 7:**
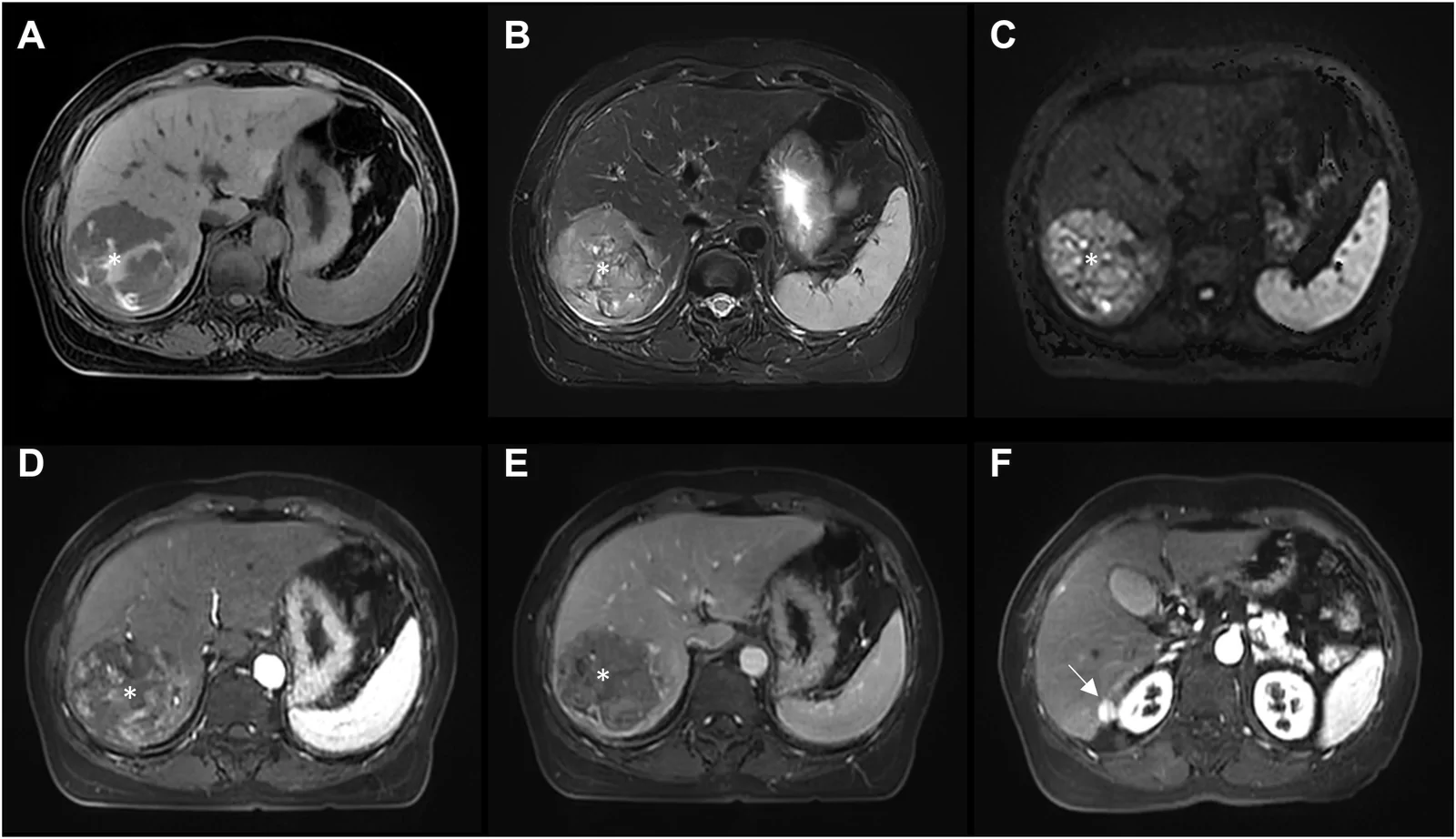
Representative preoperative MRI case and model prediction example. Axial extracellular contrast-enhanced preoperative MRI images **(A-F)** are shown for a 72-year-old woman with chronic hepatitis **(B)** A 7.4-cm mass was observed in segments S8/S7, and a 1.3-cm satellite nodule was observed in segment S6. Histopathologic analysis confirmed moderately differentiated HCC with microvascular invasion. **(A)** Precontrast T1-weighted imaging showed a 7.4-cm mildly hypointense to hypointense mass **(asterisk)** with an apparent internal hemorrhagic focus. **(B)** Precontrast T2-weighted imaging showed a mass with intermediate to high signal intensity (asterisk). **(C)** Diffusion-weighted imaging with b = 800 s/mm2 showed marked diffusion restriction (asterisk). **(D)** Arterial phase imaging showed heterogeneous marked enhancement within the mass (asterisk). **(E)** Portal venous phase imaging showed decreased enhancement of the mass, consistent with typical washout of HCC (asterisk). **(F)** Arterial phase imaging also showed a 1.3-cm satellite lesion in segment S6 (arrow). This case was from the study cohort and illustrates selected key MRI features in the final combined model and the calculation of 24-month early recurrence risk. The patient’s preoperative extracellular contrast-enhanced MRI showed tumor diameter >5 cm, satellite nodule, peritumoral hypointensity on portal venous phase images, and incomplete capsule, without intrahepatic vessel invasion. Combined with preoperative PIVKA-II, the model predicted a high risk of 24-month early recurrence, and recurrence was confirmed 8.2 weeks after surgery. This single case is illustrative only and is intended to explain model input variables and risk calculation. It does not represent overall model performance.

### 3.9 Adjuvant therapy sensitivity analysis

After postoperative adjuvant therapy was added to the final combined model in the development cohort, model performance and predictor estimates did not materially change. The C-index changed from 0.756 to 0.757, and the HRs of all six preoperative predictors changed by less than 1%. After adjustment, adjuvant therapy was not associated with DFS (HR, 1.060; 95% CI: 0.768-1.463; P = 0.723). The external validation cohort contained only 10 patients without adjuvant therapy, limiting untreated-only validation. Among external patients who received adjuvant therapy, the development-derived cutoff still separated early recurrence rates (22 of 26 high-risk patients vs one of 26 low-risk patients), although the HR remained imprecise (48.168; 95% CI: 6.409-361.991). These findings indicate that including adjuvant therapy did not materially affect the predictive structure of the preoperative model. However, because adjuvant therapy was not randomized and treatment selection may have been confounded by risk, this sensitivity analysis cannot exclude residual confounding and was not designed to evaluate the therapeutic effect of adjuvant therapy.

## 5 Discussion

This two-center retrospective cohort study developed and externally validated a preoperative MRI-clinical combined model for predicting early recurrence after hepatectomy for HCC. The final model consisted of log(PIVKA-II) and five MRI features: tumor size >5 cm, satellite nodule, peritumoral hypointensity on portal venous phase images, intrahepatic vessel invasion, and incomplete capsule. The model showed good discrimination in the development cohort, with an apparent C-index of 0.756 and a bootstrap-corrected 24-month AUC of 0.829. In the independent external validation cohort, the C-index was 0.892, the 24-month AUC was 0.978, and the Brier score was 0.087. Calibration analyses nevertheless indicated imperfect absolute risk calibration. The exploratory risk cutoff of 0.584, derived from the development cohort, separated patients with different recurrence risks in both cohorts, suggesting that the model may support preoperative risk stratification and risk communication, while absolute risk estimates require cautious interpretation before clinical use.

Compared with previous studies, the main value of this study lies not in proposing a new individual MRI sign but in integrating preoperatively available and routinely interpretable information into an independently validated risk tool. Previous HCC early recurrence prediction models often included both preoperative and postoperative factors. Postoperative models may gain explanatory value from pathologic information, but such variables are unavailable during preoperative risk communication and perioperative study design (5, 12). Previous MRI, radiomics, and deep learning studies also suggest that preoperative imaging can predict early recurrence, but some were based mainly on single-center internal splits or depended on texture analysis, deep learning features, or specific MRI feature combinations (9–11, 13). In contrast, this study emphasizes the complementarity between preoperative clinical laboratory information such as PIVKA-II and routine MRI features directly assessable by radiologists. The fixed model coefficients and development cohort-derived risk cutoff of 0.584 were directly applied to an independent external validation cohort. This design more closely reflects a real preoperative risk-communication setting and is aligned with principles of external validation and transportability assessment for prediction models (14, 15).

The predictors retained in the final model have plausible clinical and imaging interpretations. PIVKA-II is associated with aggressive HCC biology and postoperative early recurrence risk (7), and log(PIVKA-II) entered the final model in this study, suggesting that serum biomarkers may complement imaging features in recurrence risk assessment. Among MRI features, tumor size >5 cm represents greater tumor burden and has been included in previous HCC early recurrence risk factor studies and MRI prediction studies (4, 9, 10, 12). Satellite nodules have been associated with early recurrence after resection in previous MRI studies (9, 10), suggesting that they may reflect a tumor phenotype related to intrahepatic dissemination. Intrahepatic vessel invasion is biologically consistent with a tendency toward vascular invasion, and recent imaging-pathology studies have shown that MRI features can help evaluate microvascular invasion of HCC and prognostically relevant grading (28). Peritumoral hypointensity on portal venous phase images, as assessed in this study, may reflect peritumoral perfusion abnormality or microinvasion-related change. Although previous studies of preoperative MRI early recurrence models and peritumoral imaging abnormalities used contrast agents, sequences, and specific phases that were not identical to those in the present study, they support, in a broader sense, an association between peritumoral imaging abnormalities and aggressive tumor biology or early recurrence risk (10, 13, 29). Incomplete capsule may indicate less constrained tumor boundaries. Recent studies of capsule-related imaging findings and MRI-based early recurrence models also suggest that capsule or boundary-related features have imaging-biologic and prognostic relevance (12, 13, 30). Thus, the MRI variables included in the final model were not only statistically selected but also clinically plausible markers of aggressive HCC phenotypes and postoperative early recurrence risk.

From a model-evaluation perspective, this study reports multiple layers of performance assessment rather than judging the model by AUC alone. In the development cohort, the combined model had a 24-month AUC of 0.838 and a Brier score of 0.163, outperforming the clinical and MRI models alone. After bootstrap internal validation, the combined model maintained good discrimination. In external validation, the final model was applied to the Suining cohort without refitting, baseline-survival recalibration, or cutoff re-estimation, allowing a direct but stringent assessment of transportability to another center (15). The combined model had high C-index and AUC in the external validation cohort, but calibration curves showed risk overestimation in the lower predicted-risk range, and the clinical model had a slightly lower external Brier score than the combined model. This pattern indicates that discrimination and calibration addressed different questions: C-index and AUC assessed whether patients were ranked in the correct risk order, whereas calibration assessed whether predicted absolute risks matched observed risks (26). The negative external calibration intercept was consistent with systematic overestimation of absolute risk, while the calibration slope greater than 1 suggested that predicted probabilities were not spread as widely as the observed outcome gradient. Several factors may have contributed to this transportability limitation, including use of the development cohort baseline survival without external recalibration, the small external validation sample, between-center differences in liver disease background and case mix, and postoperative treatment imbalance. Chronic hepatitis B was less frequent in the external validation cohort than in the development cohort (50.0% vs 76.0%, P < 0.001), whereas imaging cirrhosis did not differ significantly (46.8% vs 42.2%, P = 0.518). Postoperative adjuvant therapy was more frequent in the external validation cohort (83.9% vs 50.8%, P < 0.001). Because therapy was given after surgery, it was not eligible for inclusion in the main preoperative model. Nevertheless, it may have modified observed recurrence after risk classification and contributed to external baseline-hazard miscalibration. If the external center used postoperative therapy more intensively, observed recurrence could be lower than expected in some externally validated strata, including the low-risk stratum. The sensitivity analyses suggested predictor stability after adjuvant adjustment, but they cannot separate treatment effect from treatment-selection bias. Similarly, the external risk-stratification HR was driven by sparse events in the low-risk reference arm. It supports the direction of separation but should not be read as a precise clinical effect size. The findings therefore indicate that the combined model has good risk-ranking ability, whereas its absolute risk estimates require recalibration and further evaluation in larger external cohorts before use for individualized decision-making.

From a potential clinical-use perspective, the model uses preoperatively available information to identify patients at increased risk of recurrence within 24 months after surgery. Risk stratification showed that the exploratory cutoff of 0.584 separated patients into groups with distinct early recurrence rates and DFS in both cohorts. In addition, DCA suggested a positive net benefit across a broad range of threshold probabilities. These findings support the model as a tool for research-oriented risk communication, individualized follow-up planning, and selection of patients for perioperative treatment studies. However, the cutoff was derived in the development cohort, and its use should remain exploratory. It should not be used alone to determine treatment. Similarly, adjuvant therapy sensitivity analysis showed that including adjuvant therapy did not materially alter preoperative predictor estimates. However, because treatment was selected after surgery and was imbalanced between centers, this sensitivity analysis cannot exclude residual confounding and was not designed to evaluate the therapeutic effect of adjuvant therapy.

This study has limitations. First, the retrospective design may have introduced selection bias and unmeasured confounding. Second, although independent external validation was performed, the external validation cohort was small. In particular, only one early recurrence event occurred in the external low-risk group, making the external risk-stratification HR an unstable estimate with a wide confidence interval rather than a precise measure of clinical effect magnitude. Third, the model was developed and validated in two Chinese centers, and differences in etiologic composition, treatment strategies, and follow-up patterns may limit generalizability to other regions or populations with different etiologies. Fourth, the model focused on preoperative imaging and laboratory information and did not include postoperative pathologic or treatment variables. These factors may provide additional prognostic information, but they are not available preoperatively. Tumor number was also not evaluated as an independent predictor. This choice should not be interpreted as discounting the prognostic value of multifocality (4–6). It was made to preserve a predefined imaging framework and avoid overlap with satellite nodule assessment. Future studies with larger cohorts should examine standardized tumor-count definitions alongside satellite nodule status. Fifth, although MRI acquisition parameters were consistent between the two centers and a small-scale interreader agreement analysis was performed, imaging features still depended on manual assessment. Although the final analysis did not classify follow-up shorter than 24 months without confirmed recurrence as absence of early recurrence, the retrospective design and center-specific follow-up patterns remain potential sources of follow-up-related bias. Future studies should validate model performance in larger prospective multicenter cohorts, further optimize absolute risk calibration when necessary, and evaluate whether model-assisted risk stratification can improve follow-up or perioperative management decisions (15).

## 6 Conclusion

In conclusion, this study developed a preoperative combined model consisting of log(PIVKA-II) and MRI features for predicting early recurrence after hepatectomy for HCC. The model showed good discrimination in both the development and external validation cohorts and separated patients into subgroups with different early recurrence risks. Given the imperfect external calibration and limited size of the external validation cohort, the model is best viewed at present as a reference tool for preoperative risk stratification and study design. Prospective multicenter validation is needed before routine clinical implementation.

## Data Availability

The raw data supporting the conclusions of this article will be made available by the authors, without undue reservation.
