# Supplementary material for: Preoperative MRI features and clinical factors for predicting early recurrence of hepatocellular carcinoma after hepatectomy
Source: Front Oncol. 2026 Sep 17;16:1939820. doi: 10.3389/fonc.2026.1939820 (PMC13627032; doi:10.3389/fonc.2026.1939820)
Supplement: Supplementary file 1 [file Table1.docx]

Supplementary Material

**Preoperative MRI Features and Clinical Factors for Predicting Early Recurrence of Hepatocellular Carcinoma after Hepatectomy**

This file contains supplementary tables, the TRIPOD checklist, additional model performance details, and information about the offline web calculator package.

# 1 Supplementary Tables

**Supplementary Table S1. Normality check for continuous variables.**

*Shapiro-Wilk tests were performed for all continuous variables used in Table 1.*

| **Variable** | **All P** | **Development P** | **External P** | **Decision** | **Presentation** | **Comparison** |
| --- | --- | --- | --- | --- | --- | --- |
| Disease-free survival (months) | <.001 | <.001 | .074 | Non-normal or not normal in at least one cohort | Median (IQR) | Mann-Whitney U |
| log(CEA) | <.001 | <.001 | <.001 | Non-normal or not normal in at least one cohort | Median (IQR) | Mann-Whitney U |
| log(CA19-9) | <.001 | <.001 | <.001 | Non-normal or not normal in at least one cohort | Median (IQR) | Mann-Whitney U |
| log(AFP) | <.001 | <.001 | <.001 | Non-normal or not normal in at least one cohort | Median (IQR) | Mann-Whitney U |
| log(PIVKA-II) | <.001 | <.001 | <.001 | Non-normal or not normal in at least one cohort | Median (IQR) | Mann-Whitney U |
| ALT | <.001 | <.001 | <.001 | Non-normal or not normal in at least one cohort | Median (IQR) | Mann-Whitney U |
| log(ALP) | <.001 | <.001 | .790 | Non-normal or not normal in at least one cohort | Median (IQR) | Mann-Whitney U |
| log(GGT) | <.001 | <.001 | .085 | Non-normal or not normal in at least one cohort | Median (IQR) | Mann-Whitney U |
| Prothrombin time | <.001 | <.001 | <.001 | Non-normal or not normal in at least one cohort | Median (IQR) | Mann-Whitney U |
| Platelets | <.001 | <.001 | .009 | Non-normal or not normal in at least one cohort | Median (IQR) | Mann-Whitney U |
| NLR | <.001 | <.001 | <.001 | Non-normal or not normal in at least one cohort | Median (IQR) | Mann-Whitney U |
| log(NLR) | <.001 | <.001 | <.001 | Non-normal or not normal in at least one cohort | Median (IQR) | Mann-Whitney U |
| Total bilirubin | <.001 | <.001 | .160 | Non-normal or not normal in at least one cohort | Median (IQR) | Mann-Whitney U |
| AST/ALT ratio | <.001 | <.001 | .003 | Non-normal or not normal in at least one cohort | Median (IQR) | Mann-Whitney U |
| Main portal vein width (cm) | <.001 | <.001 | .013 | Non-normal or not normal in at least one cohort | Median (IQR) | Mann-Whitney U |

Note.-P values are from Shapiro-Wilk tests. P < .05 indicates evidence against normality. Because most continuous variables were non-normal in at least one cohort, Table 1 reports continuous variables as medians with IQRs and compares groups using the Mann-Whitney U test.

**Supplementary Table S2. Interreader agreement for MRI features.**

*Three-reader rereading assessment in 60 randomly selected patients*

# 2 Methods

| **Item** | **Description** |
| --- | --- |
| Sample | Sixty randomly selected patients were independently reread by three radiology clinicians/readers. |
| Categorical MRI features | Interreader agreement was assessed using Fleiss' kappa for three raters. |
| Continuous measurement | Main portal vein width was assessed using a two-way random-effects single-measure absolute-agreement ICC, ICC(A,1). |
| Confidence intervals | Approximate 95% confidence intervals were estimated using patient-level bootstrap resampling with 1000 repetitions. |
| Interpretation | Agreement was interpreted as poor (<0), slight (0.00-0.20), fair (0.21-0.40), moderate (0.41-0.60), substantial (0.61-0.80), or almost perfect (0.81-1.00). |

# 3 Final Model MRI Features

| **Feature** | **N complete** | **Method** | **Estimate (95% CI)** | **All-reader exact agreement** | **Interpretation** |
| --- | --- | --- | --- | --- | --- |
| Tumor size >5 cm | 60 | Fleiss' kappa | 0.775 (0.639-0.889) | 83.3% | Substantial |
| Satellite nodule | 60 | Fleiss' kappa | 0.572 (0.320-0.747) | 76.7% | Moderate |
| Intrahepatic vessel invasion | 60 | Fleiss' kappa | 0.652 (0.467-0.811) | 78.3% | Substantial |
| Peritumoral hypointensity on portal venous phase images | 60 | Fleiss' kappa | 0.743 (0.532-0.896) | 86.7% | Substantial |
| Incomplete capsule | 60 | Fleiss' kappa | 0.766 (0.609-0.888) | 83.3% | Substantial |

# 4 All Reread MRI Features

| **Feature** | **Final model feature** | **N complete** | **Method** | **Estimate (95% CI)** | **All-reader exact agreement** | **Interpretation** |
| --- | --- | --- | --- | --- | --- | --- |
| Tumor size >5 cm | Yes | 60 | Fleiss' kappa | 0.775 (0.639-0.889) | 83.3% | Substantial |
| Satellite nodule | Yes | 60 | Fleiss' kappa | 0.572 (0.320-0.747) | 76.7% | Moderate |
| Infiltrative appearance | No | 60 | Fleiss' kappa | 0.796 (0.647-0.912) | 86.7% | Substantial |
| Intrahepatic vessel invasion | Yes | 60 | Fleiss' kappa | 0.652 (0.467-0.811) | 78.3% | Substantial |
| Peritumoral hypointensity on portal venous phase images | Yes | 60 | Fleiss' kappa | 0.743 (0.532-0.896) | 86.7% | Substantial |
| Rim APHE | No | 60 | Fleiss' kappa | 0.741 (0.550-0.883) | 83.3% | Substantial |
| Incomplete capsule | Yes | 60 | Fleiss' kappa | 0.766 (0.609-0.888) | 83.3% | Substantial |
| Nodule-in-nodule architecture | No | 60 | Fleiss' kappa | 0.737 (0.549-0.883) | 85.0% | Substantial |
| Multiseptated/multinodular architecture | No | 60 | Fleiss' kappa | 0.756 (0.621-0.888) | 81.7% | Substantial |
| Necrosis or severe ischemia | No | 60 | Fleiss' kappa | 0.796 (0.665-0.908) | 85.0% | Substantial |
| Intratumoral artery | No | 60 | Fleiss' kappa | 0.786 (0.583-0.915) | 88.3% | Substantial |
| Tumor number | No | 60 | Fleiss' kappa | 1.000 (1.000-1.000) | 100.0% | Almost perfect |
| Nonrim APHE | No | 60 | Fleiss' kappa | 0.798 (0.647-0.911) | 85.0% | Substantial |
| Capsule present | No | 60 | Fleiss' kappa | 0.704 (0.503-0.847) | 83.3% | Substantial |
| Capsule enhancement | No | 60 | Fleiss' kappa | 0.720 (0.529-0.863) | 83.3% | Substantial |
| Diffusion restriction | No | 60 | Fleiss' kappa | 0.225 (-0.078-0.613) | 86.7% | Fair |
| Mild-to-moderate T2 hyperintensity | No | 60 | Fleiss' kappa | 0.608 (0.285-0.814) | 85.0% | Substantial |
| Ring enhancement | No | 60 | Fleiss' kappa | 0.495 (0.286-0.669) | 70.0% | Moderate |
| Intratumoral hemorrhage | No | 60 | Fleiss' kappa | 0.749 (0.598-0.867) | 81.7% | Substantial |
| Intratumoral fat | No | 60 | Fleiss' kappa | 0.880 (0.763-0.975) | 91.7% | Almost perfect |
| Intratumoral fat deficiency/internal nodule | No | 60 | Fleiss' kappa | 0.550 (0.293-0.746) | 76.7% | Moderate |
| Intratumoral iron deficiency/internal nodule | No | 60 | Fleiss' kappa | 0.574 (0.346-0.762) | 78.3% | Moderate |
| Focal marked T2 hyperintensity | No | 60 | Fleiss' kappa | 0.843 (0.733-0.952) | 88.3% | Almost perfect |
| Intratumoral iron deposition > liver | No | 60 | Fleiss' kappa | 0.225 (-0.071-0.588) | 86.7% | Fair |
| Blood-pool enhancement pattern | No | 60 | Fleiss' kappa | 0.415 (0.117-0.651) | 75.0% | Moderate |
| Peripheral washout | No | 60 | Fleiss' kappa | 0.479 (0.219-0.681) | 75.0% | Moderate |
| Delayed central enhancement | No | 60 | Fleiss' kappa | 0.245 (-0.078-0.518) | 76.7% | Fair |
| Targetoid diffusion restriction | No | 60 | Fleiss' kappa | 0.638 (0.259-0.869) | 88.3% | Substantial |
| Peritumoral T2 hyperintensity | No | 60 | Fleiss' kappa | 0.615 (0.410-0.775) | 78.3% | Substantial |
| APHE >=50% | No | 60 | Fleiss' kappa | 0.868 (0.733-0.971) | 91.7% | Almost perfect |
| Ascites | No | 60 | Fleiss' kappa | 0.629 (0.379-0.814) | 83.3% | Substantial |
| Imaging cirrhosis | No | 60 | Fleiss' kappa | 0.729 (0.579-0.844) | 80.0% | Substantial |
| Diffuse iron overload | No | 60 | Fleiss' kappa | 0.200 (-0.072-0.503) | 81.7% | Fair |
| Diffuse hepatic steatosis | No | 60 | Fleiss' kappa | 0.811 (0.679-0.928) | 86.7% | Almost perfect |
| Main portal vein width | No | 60 | ICC(A,1), absolute agreement | 0.885 (0.821-0.922) | NA | Almost perfect |
| Splenomegaly | No | 60 | Fleiss' kappa | 0.759 (0.585-0.891) | 86.7% | Substantial |
| Portosystemic shunt/collaterals | No | 60 | Fleiss' kappa | 0.521 (0.153-0.746) | 81.7% | Moderate |
| Varices | No | 60 | Fleiss' kappa | 0.493 (0.231-0.720) | 78.3% | Moderate |

*Note. Kappa can be low or not estimable when a feature has very low prevalence or near-constant ratings, even when raw agreement is high. These results are therefore interpreted together with all-reader exact agreement and the clinical prevalence of each feature.*

**Supplementary Table S3. Univariable Cox regression in the development cohort.**

*Cox models used 24-month administrative truncation: time = min(disease-free survival, 24 months), event = early recurrence before 24 months.*

| **Characteristic** | **Unit/Comparison** | **N** | **Events** | **HR (95% CI)** | **P Value** | **P<0.10** | **Status** |
| --- | --- | --- | --- | --- | --- | --- | --- |
| **Clinical and Laboratory Characteristics** |  |  |  |  |  |  |  |
| Chronic hepatitis B | 1 vs 0 | 258 | 153 | 1.04 (0.71-1.52) | .834 | No | OK |
| log(CEA) | per 1-unit increase | 258 | 153 | 1.68 (1.46-1.94) | <.001 | Yes | OK |
| log(CA19-9) | per 1-unit increase | 258 | 153 | 1.37 (1.25-1.50) | <.001 | Yes | OK |
| log(AFP) | per 1-unit increase | 258 | 153 | 1.11 (1.06-1.16) | <.001 | Yes | OK |
| AFP >400 | 1 vs 0 | 258 | 153 | 1.94 (1.40-2.68) | <.001 | Yes | OK |
| log(PIVKA-II) | per 1-unit increase | 258 | 153 | 1.33 (1.25-1.42) | <.001 | Yes | OK |
| AST >40 | 1 vs 0 | 258 | 153 | 1.51 (1.10-2.08) | .011 | Yes | OK |
| ALT | per 1-unit increase | 258 | 153 | 1.00 (1.00-1.00) | .571 | No | OK |
| log(ALP) | per 1-unit increase | 258 | 153 | 1.30 (1.05-1.60) | .015 | Yes | OK |
| log(GGT) | per 1-unit increase | 258 | 153 | 1.45 (1.23-1.72) | <.001 | Yes | OK |
| Prothrombin time | per 1-s increase | 258 | 153 | 1.01 (0.90-1.13) | .929 | No | OK |
| Platelets | per 1-unit increase | 258 | 153 | 1.00 (1.00-1.00) | .002 | Yes | OK |
| NLR | per 1-unit increase | 258 | 153 | 1.01 (0.99-1.04) | .347 | No | OK |
| log(NLR) | per 1-unit increase | 258 | 153 | 1.12 (0.84-1.49) | .445 | No | OK |
| HBsAb positive | 1 vs 0 | 258 | 153 | 0.84 (0.53-1.35) | .480 | No | OK |
| HBeAg positive | 1 vs 0 | 258 | 153 | 0.97 (0.55-1.71) | .910 | No | OK |
| Total bilirubin | per 1-unit increase | 258 | 153 | 0.99 (0.98-1.01) | .608 | No | OK |
| AST/ALT ratio | per 1-unit increase | 258 | 153 | 1.34 (1.13-1.57) | <.001 | Yes | OK |
| ALBI grade 2 or 3 | 1 vs 0 | 258 | 153 | 1.38 (1.00-1.91) | .051 | Yes | OK |
| **Preoperative MRI Characteristics** |  |  |  |  |  |  |  |
| Tumor size >5 cm | 1 vs 0 | 258 | 153 | 2.94 (2.12-4.08) | <.001 | Yes | OK |
| Satellite nodule | 1 vs 0 | 258 | 153 | 2.98 (2.05-4.32) | <.001 | Yes | OK |
| Infiltrative appearance | 1 vs 0 | 258 | 153 | 2.50 (1.81-3.46) | <.001 | Yes | OK |
| Intrahepatic vessel invasion | 1 vs 0 | 258 | 153 | 3.23 (2.19-4.77) | <.001 | Yes | OK |
| Peritumoral hypointensity on portal venous phase images | 1 vs 0 | 258 | 153 | 2.85 (2.02-4.01) | <.001 | Yes | OK |
| Rim arterial phase hyperenhancement | 1 vs 0 | 258 | 153 | 0.91 (0.65-1.28) | .583 | No | OK |
| Incomplete capsule | 1 vs 0 | 258 | 153 | 2.12 (1.46-3.08) | <.001 | Yes | OK |
| Nodule-in-nodule architecture | 1 vs 0 | 258 | 153 | 2.59 (1.85-3.63) | <.001 | Yes | OK |
| Multiseptated or multinodular architecture | 1 vs 0 | 258 | 153 | 2.24 (1.62-3.09) | <.001 | Yes | OK |
| Necrosis or severe ischemia | 1 vs 0 | 258 | 153 | 1.84 (1.34-2.53) | <.001 | Yes | OK |
| Intratumoral artery | 1 vs 0 | 258 | 153 | 1.91 (1.37-2.68) | <.001 | Yes | OK |
| Nonrim arterial phase hyperenhancement | 1 vs 0 | 258 | 153 | 1.18 (0.85-1.63) | .324 | No | OK |
| Capsule present | 1 vs 0 | 258 | 153 | 0.65 (0.46-0.92) | .016 | Yes | OK |
| Capsule enhancement | 1 vs 0 | 258 | 153 | 0.68 (0.48-0.96) | .028 | Yes | OK |
| Diffusion restriction | 1 vs 0 | 258 | 153 | 1.39 (0.34-5.61) | .644 | No | OK |
| Mild-to-moderate T2 hyperintensity | 1 vs 0 | 258 | 153 | 0.80 (0.52-1.24) | .314 | No | OK |
| Ring enhancement | 1 vs 0 | 258 | 153 | 1.50 (1.07-2.12) | .020 | Yes | OK |
| Intratumoral hemorrhage | 1 vs 0 | 258 | 153 | 1.67 (1.22-2.30) | .002 | Yes | OK |
| Intratumoral fat | 1 vs 0 | 258 | 153 | 1.00 (0.71-1.40) | .992 | No | OK |
| Intratumoral fat deficiency or internal nodule | 1 vs 0 | 258 | 153 | 1.04 (0.65-1.66) | .874 | No | OK |
| Intratumoral iron deficiency or internal nodule | 1 vs 0 | 258 | 153 | 0.75 (0.49-1.15) | .191 | No | OK |
| Focal marked T2 hyperintensity | 1 vs 0 | 258 | 153 | 0.90 (0.66-1.24) | .530 | No | OK |
| Intratumoral iron deposition greater than liver | 1 vs 0 | 258 | 153 | 0.88 (0.41-1.88) | .738 | No | OK |
| Blood-pool enhancement pattern | 1 vs 0 | 258 | 153 | 0.76 (0.42-1.37) | .364 | No | OK |
| Peripheral washout | 1 vs 0 | 258 | 153 | 0.59 (0.37-0.93) | .024 | Yes | OK |
| Delayed central enhancement | 1 vs 0 | 258 | 153 | 1.50 (0.77-2.95) | .235 | No | OK |
| Targetoid diffusion restriction | 1 vs 0 | 258 | 153 | 1.09 (0.60-1.96) | .781 | No | OK |
| Peritumoral T2 hyperintensity | 1 vs 0 | 258 | 153 | 2.51 (1.81-3.49) | <.001 | Yes | OK |
| APHE proportion <50% | 1 vs 0 | 258 | 153 | 1.82 (1.31-2.51) | <.001 | Yes | OK |
| Ascites | 1 vs 0 | 258 | 153 | 1.02 (0.59-1.76) | .955 | No | OK |
| Imaging cirrhosis | 1 vs 0 | 258 | 153 | 1.00 (0.72-1.37) | .987 | No | OK |
| Diffuse iron overload | 1 vs 0 | 258 | 153 | 0.83 (0.42-1.62) | .579 | No | OK |
| Diffuse hepatic steatosis | 1 vs 0 | 258 | 153 | 0.91 (0.65-1.26) | .555 | No | OK |
| Main portal vein width (cm) | per 1-cm increase | 258 | 153 | 0.92 (0.48-1.77) | .807 | No | OK |
| Splenomegaly | 1 vs 0 | 258 | 153 | 1.00 (0.69-1.46) | 1.000 | No | OK |
| Portosystemic shunt/collaterals | 1 vs 0 | 258 | 153 | 1.05 (0.61-1.78) | .865 | No | OK |
| Varices | 1 vs 0 | 257 | 153 | 0.87 (0.53-1.45) | .603 | No | OK |

Note.-The development cohort included 258 patients and 153 early recurrence events. Postoperative adjuvant therapy was excluded from the main screening variables and reserved for sensitivity analysis. Variables with P<0.10 are marked as candidate variables for subsequent multivariable modeling, subject to clinical relevance and collinearity assessment. APHE = arterial phase hyperenhancement; HR = hazard ratio; NLR = neutrophil-to-lymphocyte ratio.

**Supplementary Table S4. Univariable logistic regression in the development cohort.**

*Outcome: early recurrence before 24 months. Each candidate variable was entered into a separate logistic regression model.*

| **Characteristic** | **Unit/Comparison** | **N** | **Events** | **OR (95% CI)** | **P Value** | **P<0.10** | **Status** |
| --- | --- | --- | --- | --- | --- | --- | --- |
| **Clinical and Laboratory Characteristics** |  |  |  |  |  |  |  |
| Chronic hepatitis B | 1 vs 0 | 258 | 153 | 1.17 (0.65-2.08) | .600 | No | OK |
| log(CEA) | per 1-unit increase | 258 | 153 | 2.31 (1.65-3.24) | <.001 | Yes | OK |
| log(CA19-9) | per 1-unit increase | 258 | 153 | 1.70 (1.38-2.10) | <.001 | Yes | OK |
| log(AFP) | per 1-unit increase | 258 | 153 | 1.13 (1.04-1.23) | .004 | Yes | OK |
| AFP >400 | 1 vs 0 | 258 | 153 | 2.12 (1.21-3.71) | .009 | Yes | OK |
| log(PIVKA-II) | per 1-unit increase | 258 | 153 | 1.54 (1.36-1.75) | <.001 | Yes | OK |
| AST >40 | 1 vs 0 | 258 | 153 | 1.60 (0.97-2.65) | .067 | Yes | OK |
| ALT | per 1-unit increase | 258 | 153 | 1.00 (1.00-1.01) | .743 | No | OK |
| log(ALP) | per 1-unit increase | 258 | 153 | 1.98 (1.14-3.44) | .016 | Yes | OK |
| log(GGT) | per 1-unit increase | 258 | 153 | 1.73 (1.29-2.33) | <.001 | Yes | OK |
| Prothrombin time | per 1-s increase | 258 | 153 | 0.96 (0.79-1.17) | .704 | No | OK |
| Platelets | per 1-unit increase | 258 | 153 | 1.00 (1.00-1.01) | .012 | Yes | OK |
| NLR | per 1-unit increase | 258 | 153 | 1.04 (0.98-1.10) | .229 | No | OK |
| log(NLR) | per 1-unit increase | 258 | 153 | 1.21 (0.74-1.98) | .436 | No | OK |
| HBsAb positive | 1 vs 0 | 258 | 153 | 0.84 (0.41-1.70) | .622 | No | OK |
| HBeAg positive | 1 vs 0 | 258 | 153 | 0.99 (0.41-2.41) | .983 | No | OK |
| Total bilirubin | per 1-unit increase | 258 | 153 | 1.00 (0.97-1.02) | .838 | No | OK |
| AST/ALT ratio | per 1-unit increase | 258 | 153 | 1.92 (1.22-3.00) | .004 | Yes | OK |
| ALBI grade 2 or 3 | 1 vs 0 | 258 | 153 | 1.82 (1.06-3.13) | .029 | Yes | OK |
| **Preoperative MRI Characteristics** |  |  |  |  |  |  |  |
| Tumor size >5 cm | 1 vs 0 | 258 | 153 | 4.58 (2.65-7.93) | <.001 | Yes | OK |
| Satellite nodule | 1 vs 0 | 258 | 153 | 5.45 (2.21-13.44) | <.001 | Yes | OK |
| Infiltrative appearance | 1 vs 0 | 258 | 153 | 3.97 (2.18-7.23) | <.001 | Yes | OK |
| Intrahepatic vessel invasion | 1 vs 0 | 258 | 153 | 4.54 (1.83-11.27) | .001 | Yes | OK |
| Peritumoral hypointensity on portal venous phase images | 1 vs 0 | 258 | 153 | 10.00 (3.83-26.09) | <.001 | Yes | OK |
| Rim arterial phase hyperenhancement | 1 vs 0 | 258 | 153 | 0.86 (0.50-1.48) | .592 | No | OK |
| Incomplete capsule | 1 vs 0 | 258 | 153 | 3.07 (1.80-5.24) | <.001 | Yes | OK |
| Nodule-in-nodule architecture | 1 vs 0 | 258 | 153 | 4.66 (2.30-9.45) | <.001 | Yes | OK |
| Multiseptated or multinodular architecture | 1 vs 0 | 258 | 153 | 3.45 (2.02-5.89) | <.001 | Yes | OK |
| Necrosis or severe ischemia | 1 vs 0 | 258 | 153 | 1.98 (1.19-3.30) | .008 | Yes | OK |
| Intratumoral artery | 1 vs 0 | 258 | 153 | 2.74 (1.48-5.08) | .001 | Yes | OK |
| Nonrim arterial phase hyperenhancement | 1 vs 0 | 258 | 153 | 1.18 (0.71-1.95) | .528 | No | OK |
| Capsule present | 1 vs 0 | 258 | 153 | 0.42 (0.22-0.79) | .007 | Yes | OK |
| Capsule enhancement | 1 vs 0 | 258 | 153 | 0.47 (0.25-0.86) | .015 | Yes | OK |
| Diffusion restriction | 1 vs 0 | 258 | 153 | 1.47 (0.20-10.57) | .704 | No | OK |
| Mild-to-moderate T2 hyperintensity | 1 vs 0 | 258 | 153 | 0.69 (0.33-1.46) | .334 | No | OK |
| Ring enhancement | 1 vs 0 | 258 | 153 | 1.77 (0.98-3.20) | .056 | Yes | OK |
| Intratumoral hemorrhage | 1 vs 0 | 258 | 153 | 1.86 (1.11-3.11) | .019 | Yes | OK |
| Intratumoral fat | 1 vs 0 | 258 | 153 | 1.01 (0.60-1.71) | .960 | No | OK |
| Intratumoral fat deficiency or internal nodule | 1 vs 0 | 258 | 153 | 0.90 (0.44-1.86) | .780 | No | OK |
| Intratumoral iron deficiency or internal nodule | 1 vs 0 | 258 | 153 | 0.60 (0.28-1.28) | .185 | No | OK |
| Focal marked T2 hyperintensity | 1 vs 0 | 258 | 153 | 0.80 (0.48-1.33) | .389 | No | OK |
| Intratumoral iron deposition greater than liver | 1 vs 0 | 258 | 153 | 0.96 (0.30-3.11) | .944 | No | OK |
| Blood-pool enhancement pattern | 1 vs 0 | 258 | 153 | 0.73 (0.31-1.72) | .467 | No | OK |
| Peripheral washout | 1 vs 0 | 258 | 153 | 0.52 (0.22-1.22) | .132 | No | OK |
| Delayed central enhancement | 1 vs 0 | 258 | 153 | 1.25 (0.41-3.84) | .697 | No | OK |
| Targetoid diffusion restriction | 1 vs 0 | 258 | 153 | 1.03 (0.41-2.62) | .947 | No | OK |
| Peritumoral T2 hyperintensity | 1 vs 0 | 258 | 153 | 4.31 (2.25-8.25) | <.001 | Yes | OK |
| APHE proportion <50% | 1 vs 0 | 258 | 153 | 2.12 (1.22-3.69) | .008 | Yes | OK |
| Ascites | 1 vs 0 | 258 | 153 | 0.96 (0.41-2.24) | .919 | No | OK |
| Imaging cirrhosis | 1 vs 0 | 258 | 153 | 1.02 (0.62-1.69) | .926 | No | OK |
| Diffuse iron overload | 1 vs 0 | 258 | 153 | 0.76 (0.28-2.03) | .582 | No | OK |
| Diffuse hepatic steatosis | 1 vs 0 | 258 | 153 | 1.02 (0.61-1.71) | .946 | No | OK |
| Main portal vein width (cm) | per 1-cm increase | 258 | 153 | 0.83 (0.29-2.41) | .730 | No | OK |
| Splenomegaly | 1 vs 0 | 258 | 153 | 1.06 (0.58-1.92) | .854 | No | OK |
| Portosystemic shunt/collaterals | 1 vs 0 | 258 | 153 | 1.03 (0.45-2.40) | .940 | No | OK |
| Varices | 1 vs 0 | 257 | 153 | 0.88 (0.41-1.89) | .734 | No | OK |

Note.-The development cohort included 258 patients and 153 early recurrence events. Postoperative adjuvant therapy and HBsAg positivity were excluded from the main screening variables. Odds ratios, Wald 95% CIs, and P values were calculated from univariate logistic regression models implemented directly in Python using iteratively reweighted least squares. Variables with P<0.10 are marked as candidates for subsequent modeling, subject to clinical relevance and collinearity assessment.

**Supplementary Table S5. Candidate-variable selection.**

*Development cohort: Sichuan North Medical Center; outcome: early recurrence <24 months*

Selection Rules

Study setting: The North Sichuan Medical College cohort was used as the development cohort and the Suining cohort as the external validation cohort; candidate-variable screening was performed only in the development cohort.

Outcome: Early recurrence was defined as recurrence within 24 months after surgery.

Statistical rule: Variables with P<0.10 in either univariable Cox or logistic regression entered the statistical candidate pool.

Clinical retention rule: AFP, PIVKA-II, ALBI grade, NLR, tumor size, APHE, satellite nodule, peritumoral hypointensity on portal venous phase images, necrosis, and related variables were retained or conditionally retained according to clinical and literature relevance.

Design exclusions: Tumor number was not analyzed as a candidate variable; postoperative adjuvant therapy was reserved for sensitivity analysis; HBsAg positivity had been removed from the dataset.

Next step: Redundancy, collinearity, and event-number constraints were assessed, and highly similar expressions such as AFP/log(AFP), NLR/log(NLR), APHE-related variables, and capsule-related variables were not entered simultaneously.

**Summary:** 28 variables were selected by statistical or combined statistical-clinical criteria; 4 variables were conditionally retained because of clinical/literature importance; 24 variables were not selected at this screening step.

Recommended Candidate Pool

| **label** | **source_variable** | **Domain** | **Cox P** | **Logistic P** | **Decision** | **Recommended handling** |
| --- | --- | --- | --- | --- | --- | --- |
| AFP >400 | AFP >400 ng/mL | Clinical laboratory | <.001 | .009 | Selected | Both AFP expressions were temporarily retained; one expression was selected later based on linearity, AIC/BIC, LASSO, or clinical interpretability. |
| ALBI grade 2 or 3 | ALBI grade 2 or 3 | Clinical laboratory | .051 | .029 | Selected | Entered the candidate pool; collinearity and event-number constraints were checked before multivariable modeling. |
| AST >40 | AST >40 IU/L | Clinical laboratory | .011 | .067 | Selected | Entered the candidate pool; collinearity and event-number constraints were checked before multivariable modeling. |
| AST/ALT ratio | AST/ALT ratio | Clinical laboratory | <.001 | .004 | Selected | Entered the candidate pool; collinearity and event-number constraints were checked before multivariable modeling. |
| Platelets | Platelet count (continuous) | Clinical laboratory | .002 | .012 | Selected | Because the platelet unit was small, rescaling per 10^9/L or per 50-unit increment was recommended if entered into a model. |
| log(AFP) | log_AFP | Clinical laboratory | <.001 | .004 | Selected | Both AFP expressions were temporarily retained; one expression was selected later based on linearity, AIC/BIC, LASSO, or clinical interpretability. |
| log(ALP) | log_ALP | Clinical laboratory | .015 | .016 | Selected | Entered the candidate pool; collinearity and event-number constraints were checked before multivariable modeling. |
| log(CA19-9) | log_CA199 | Clinical laboratory | <.001 | <.001 | Selected | Entered the candidate pool; collinearity and event-number constraints were checked before multivariable modeling. |
| log(CEA) | log_CEA | Clinical laboratory | <.001 | <.001 | Selected | Entered the candidate pool; collinearity and event-number constraints were checked before multivariable modeling. |
| log(GGT) | log_GGT | Clinical laboratory | <.001 | <.001 | Selected | Entered the candidate pool; collinearity and event-number constraints were checked before multivariable modeling. |
| log(PIVKA-II) | log_PIVKAII | Clinical laboratory | <.001 | <.001 | Selected | Entered the candidate pool; collinearity and event-number constraints were checked before multivariable modeling. |
| APHE proportion <50% | aphe_lt50 | MRI enhancement | <.001 | .008 | Selected | APHE-related variables should not be entered simultaneously; APHE <50% from the reference model was prioritized. |
| Capsule enhancement | capsule_enhancement | MRI enhancement | .028 | .015 | Selected | Collinearity/correlation with capsule-related variables was checked, and one expression was selected if necessary. |
| Peritumoral hypointensity on portal venous phase images | peritumoral_hypoenhancement_pvp | MRI enhancement | <.001 | <.001 | Selected | Entered the candidate pool; collinearity and event-number constraints were checked before multivariable modeling. |
| Ring enhancement | ring_enhancement | MRI enhancement | .020 | .056 | Selected | Entered the candidate pool; collinearity and event-number constraints were checked before multivariable modeling. |
| Intratumoral hemorrhage | intratumoral_hemorrhage | MRI internal architecture | .002 | .019 | Selected | Entered the candidate pool; collinearity and event-number constraints were checked before multivariable modeling. |
| Multiseptated or multinodular architecture | multiseptated_or_multinodular_architecture | MRI internal architecture | <.001 | <.001 | Selected | Entered the candidate pool; collinearity and event-number constraints were checked before multivariable modeling. |
| Necrosis or severe ischemia | necrosis_or_severe_ischemia | MRI internal architecture | <.001 | .008 | Selected | Entered the candidate pool; collinearity and event-number constraints were checked before multivariable modeling. |
| Nodule-in-nodule architecture | nodule_in_nodule | MRI internal architecture | <.001 | <.001 | Selected | Entered the candidate pool; collinearity and event-number constraints were checked before multivariable modeling. |
| Intrahepatic vessel invasion | intrahepatic_vessel_invasion | MRI invasion | <.001 | .001 | Selected | Entered the candidate pool; collinearity and event-number constraints were checked before multivariable modeling. |
| Capsule present | capsule_present | MRI morphology | .016 | .007 | Selected | Collinearity/correlation with capsule-related variables was checked, and one expression was selected if necessary. |
| Incomplete capsule | incomplete_capsule | MRI morphology | <.001 | <.001 | Selected | Collinearity/correlation with capsule-related variables was checked, and one expression was selected if necessary. |
| Infiltrative appearance | infiltrative_appearance | MRI morphology | <.001 | <.001 | Selected | Entered the candidate pool; collinearity and event-number constraints were checked before multivariable modeling. |
| Satellite nodule | satellite_nodule | MRI morphology | <.001 | <.001 | Selected | Entered the candidate pool; collinearity and event-number constraints were checked before multivariable modeling. |
| Tumor size >5 cm | tumor_size_gt5cm | MRI morphology | <.001 | <.001 | Selected | Entered the candidate pool; collinearity and event-number constraints were checked before multivariable modeling. |
| Peritumoral T2 hyperintensity | peritumoral_t2_hyperintensity | MRI peritumoral feature | <.001 | <.001 | Selected | Entered the candidate pool; collinearity and event-number constraints were checked before multivariable modeling. |
| Intratumoral artery | intratumoral_artery | Other candidate variable | <.001 | .001 | Selected | Entered the candidate pool; collinearity and event-number constraints were checked before multivariable modeling. |
| Peripheral washout | peripheral_washout | Other candidate variable | .024 | .132 | Selected | Entered the candidate pool; collinearity and event-number constraints were checked before multivariable modeling. |
| NLR | NLR | Inflammation marker | .347 | .229 | Conditionally retained | Clinically retained candidate; one of NLR and log(NLR) was selected later. |
| log(NLR) | log_NLR | Inflammation marker | .445 | .436 | Conditionally retained | Clinically retained candidate; one of NLR and log(NLR) was selected later. |
| Nonrim arterial phase hyperenhancement | nonrim_aphe | MRI enhancement | .324 | .528 | Conditionally retained | APHE-related variables should not be entered simultaneously; APHE <50% from the reference model was prioritized. |
| Rim arterial phase hyperenhancement | rim_aphe | MRI enhancement | .583 | .592 | Conditionally retained | APHE-related variables should not be entered simultaneously; APHE <50% from the reference model was prioritized. |

Detailed Selection Record

| **label** | **source_variable** | **Domain** | **Cox P** | **Logistic P** | **P<0.10 by either model** | **Clinical/literature important** | **Decision** | **Literature support** | **Rationale / next-step note** |
| --- | --- | --- | --- | --- | --- | --- | --- | --- | --- |
| AFP >400 | AFP >400 ng/mL | Clinical laboratory | <.001 | .009 | Yes | Yes | Selected | R2, R3 | AFP >400 ng/mL is a commonly used high-risk cutoff and was considered as an alternative expression to log(AFP). It met both univariable and clinical/literature criteria and entered the candidate pool. |
| ALBI grade 2 or 3 | ALBI grade 2 or 3 | Clinical laboratory | .051 | .029 | Yes | Yes | Selected | R4, R5, R6 | ALBI grade reflects hepatic functional reserve and has been associated with recurrence or survival after HCC resection. It met both univariable and clinical/literature criteria and entered the candidate pool. |
| AST >40 | AST >40 IU/L | Clinical laboratory | .011 | .067 | Yes | No | Selected | NA | Met the univariable Cox or logistic regression criterion of P<0.10 and entered the statistical candidate pool; collinearity and event-number constraints required further assessment. |
| AST/ALT ratio | AST/ALT ratio | Clinical laboratory | <.001 | .004 | Yes | No | Selected | NA | Met the univariable Cox or logistic regression criterion of P<0.10 and entered the statistical candidate pool; collinearity and event-number constraints required further assessment. |
| Platelets | Platelet count (continuous) | Clinical laboratory | .002 | .012 | Yes | No | Selected | NA | Met the univariable Cox or logistic regression criterion of P<0.10 and entered the statistical candidate pool; collinearity and event-number constraints required further assessment. |
| log(AFP) | log_AFP | Clinical laboratory | <.001 | .004 | Yes | Yes | Selected | R2, R3 | AFP is a commonly used serum marker for early recurrence after HCC surgery; together with PIVKA-II, it may reflect aggressive tumor biology. It met both univariable and clinical/literature criteria and entered the candidate pool. |
| log(ALP) | log_ALP | Clinical laboratory | .015 | .016 | Yes | No | Selected | NA | Met the univariable Cox or logistic regression criterion of P<0.10 and entered the statistical candidate pool; collinearity and event-number constraints required further assessment. |
| log(CA19-9) | log_CA199 | Clinical laboratory | <.001 | <.001 | Yes | No | Selected | NA | Met the univariable Cox or logistic regression criterion of P<0.10 and entered the statistical candidate pool; collinearity and event-number constraints required further assessment. |
| log(CEA) | log_CEA | Clinical laboratory | <.001 | <.001 | Yes | No | Selected | NA | Met the univariable Cox or logistic regression criterion of P<0.10 and entered the statistical candidate pool; collinearity and event-number constraints required further assessment. |
| log(GGT) | log_GGT | Clinical laboratory | <.001 | <.001 | Yes | No | Selected | NA | Met the univariable Cox or logistic regression criterion of P<0.10 and entered the statistical candidate pool; collinearity and event-number constraints required further assessment. |
| log(PIVKA-II) | log_PIVKAII | Clinical laboratory | <.001 | <.001 | Yes | Yes | Selected | R2, R3 | PIVKA-II has been associated with early recurrence, tumor aggressiveness, and prognosis, and was retained as a candidate. It met both univariable and clinical/literature criteria and entered the candidate pool. |
| APHE proportion <50% | aphe_lt50 | MRI enhancement | <.001 | .008 | Yes | Yes | Selected | R1, R9, R10, R11 | APHE extent <50% was derived from an MRI recurrence-prediction reference model and reflects enhancement heterogeneity. It met both univariable and clinical/literature criteria and entered the candidate pool. |
| Capsule enhancement | capsule_enhancement | MRI enhancement | .028 | .015 | Yes | Yes | Selected | R9, R10, R11 | Capsule enhancement is related to capsule integrity and required selection or dimension reduction with other capsule variables. It met both univariable and clinical/literature criteria and entered the candidate pool. |
| Peritumoral hypointensity on portal venous phase images | peritumoral_hypoenhancement_pvp | MRI enhancement | <.001 | <.001 | Yes | Yes | Selected | R9, R10, R11 | Peritumoral hypointensity on portal venous phase images may reflect peritumoral perfusion alteration and has been associated with MVI or early recurrence risk. It met both univariable and clinical/literature criteria and entered the candidate pool. |
| Ring enhancement | ring_enhancement | MRI enhancement | .020 | .056 | Yes | Yes | Selected | R9, R10, R11 | Ring enhancement may indicate an atypical or aggressive enhancement pattern and was close to or met the statistical screening criterion. It met both univariable and clinical/literature criteria and entered the candidate pool. |
| Intratumoral hemorrhage | intratumoral_hemorrhage | MRI internal architecture | .002 | .019 | Yes | No | Selected | NA | Met the univariable Cox or logistic regression criterion of P<0.10 and entered the statistical candidate pool; collinearity and event-number constraints required further assessment. |
| Multiseptated or multinodular architecture | multiseptated_or_multinodular_architecture | MRI internal architecture | <.001 | <.001 | Yes | Yes | Selected | R9, R10, R11, R12 | Multiseptated or multinodular architecture may reflect macroscopic structural heterogeneity and was positive in statistical screening. It met both univariable and clinical/literature criteria and entered the candidate pool. |
| Necrosis or severe ischemia | necrosis_or_severe_ischemia | MRI internal architecture | <.001 | .008 | Yes | Yes | Selected | R9, R10, R11 | Necrosis or severe ischemia suggests intratumoral heterogeneity and aggressiveness and was retained as a candidate. It met both univariable and clinical/literature criteria and entered the candidate pool. |
| Nodule-in-nodule architecture | nodule_in_nodule | MRI internal architecture | <.001 | <.001 | Yes | Yes | Selected | R9, R10, R11 | Nodule-in-nodule architecture suggests intratumoral evolution and heterogeneity and was positive in statistical screening. It met both univariable and clinical/literature criteria and entered the candidate pool. |
| Intrahepatic vessel invasion | intrahepatic_vessel_invasion | MRI invasion | <.001 | .001 | Yes | Yes | Selected | R9, R10, R11 | Intrahepatic vessel invasion is a direct imaging sign related to recurrence risk and was retained as a candidate. It met both univariable and clinical/literature criteria and entered the candidate pool. |
| Capsule present | capsule_present | MRI morphology | .016 | .007 | Yes | Yes | Selected | R9, R10, R11 | Capsule-related findings are commonly assessed morphologic MRI features in HCC and were positive in statistical screening. They met both univariable and clinical/literature criteria and entered the candidate pool. |
| Incomplete capsule | incomplete_capsule | MRI morphology | <.001 | <.001 | Yes | Yes | Selected | R9, R10, R11 | Incomplete capsule is related to invasive margins and postoperative recurrence risk, but required collinearity-based selection with visible capsule/capsule enhancement. It met both univariable and clinical/literature criteria and entered the candidate pool. |
| Infiltrative appearance | infiltrative_appearance | MRI morphology | <.001 | <.001 | Yes | Yes | Selected | R9, R10, R11 | Infiltrative appearance suggests ill-defined margins and aggressive growth, with both statistical and imaging relevance. It met both univariable and clinical/literature criteria and entered the candidate pool. |
| Satellite nodule | satellite_nodule | MRI morphology | <.001 | <.001 | Yes | Yes | Selected | R9, R11, R13 | Satellite nodule suggests a tendency toward intrahepatic spread and is an important imaging/pathologic correlate of early recurrence. It met both univariable and clinical/literature criteria and entered the candidate pool. |
| Tumor size >5 cm | tumor_size_gt5cm | MRI morphology | <.001 | <.001 | Yes | Yes | Selected | R1, R13 | Tumor size is a stable clinicoradiologic risk factor in HCC recurrence models and was prespecified as >5 cm in this study. It met both univariable and clinical/literature criteria and entered the candidate pool. |
| Peritumoral T2 hyperintensity | peritumoral_t2_hyperintensity | MRI peritumoral feature | <.001 | <.001 | Yes | Yes | Selected | R9, R10, R11 | Peritumoral T2 hyperintensity may reflect peritumoral edema or microenvironmental change and was positive in statistical screening. It met both univariable and clinical/literature criteria and entered the candidate pool. |
| Intratumoral artery | intratumoral_artery | Other candidate variable | <.001 | .001 | Yes | No | Selected | NA | Met the univariable Cox or logistic regression criterion of P<0.10 and entered the statistical candidate pool; collinearity and event-number constraints required further assessment. |
| Peripheral washout | peripheral_washout | Other candidate variable | .024 | .132 | Yes | No | Selected | NA | Met the univariable Cox or logistic regression criterion of P<0.10 and entered the statistical candidate pool; collinearity and event-number constraints required further assessment. |
| NLR | NLR | Inflammation marker | .347 | .229 | No | Yes | Conditionally retained | R7, R8 | NLR represents systemic inflammatory status, and inflammation-related markers are often considered in studies of recurrence or prognosis after HCC resection. Although P was not <0.10, literature support justified conditional retention; one of NLR and log(NLR) was selected for sensitivity or penalized screening. |
| log(NLR) | log_NLR | Inflammation marker | .445 | .436 | No | Yes | Conditionally retained | R7, R8 | Log transformation can be considered when NLR is skewed; one of raw NLR and log(NLR) was selected to avoid duplicate model entry. Although P was not <0.10, literature support justified conditional retention. |
| Nonrim arterial phase hyperenhancement | nonrim_aphe | MRI enhancement | .324 | .528 | No | Yes | Conditionally retained | R9, R10, R11 | APHE type is a basic liver MRI feature; if APHE <50% was already entered, duplicate APHE expressions were avoided. Because APHE <50% had already entered the pool, this variable was usually not entered simultaneously. |
| Rim arterial phase hyperenhancement | rim_aphe | MRI enhancement | .583 | .592 | No | Yes | Conditionally retained | R9, R10, R11 | Rim APHE may indicate a more aggressive imaging phenotype, but needed to be distinguished from APHE <50% and ring enhancement. Because APHE <50% had already entered the pool, this variable was usually not entered simultaneously. |
| Postoperative adjuvant therapy | postoperative_adjuvant_therapy | Sensitivity analysis | NA | NA | No | Contextual | Reserved for sensitivity analysis | NA | Postoperative adjuvant therapy occurred after surgery and was not used as a main screening variable for the preoperative prediction model; it was used later in sensitivity analysis. |
| Tumor number | tumor_number | Design decision | NA | NA | No | Yes | Excluded by design | R9, R11, R13 | Tumor number was not analyzed as a candidate variable as prespecified; both solitary and multiple tumors met eligibility criteria, and the largest lesion diameter was used for tumor size in multiple tumors. |
| HBsAg positive | HBsAg positivity | Data cleaning | NA | NA | No | Contextual | Removed before this step | NA | This variable had been removed from both development and external validation datasets; it was not suitable for stable modeling because of insufficient variation or nonestimability. |
| ALT | ALT (continuous) | Clinical laboratory | .571 | .743 | No | No | Not selected | NA | P was not <0.10 and the variable was not judged to require clinical/literature-based retention in this round. |
| Prothrombin time | Prothrombin time | Clinical laboratory | .929 | .704 | No | No | Not selected | NA | P was not <0.10 and the variable was not judged to require clinical/literature-based retention in this round. |
| Total bilirubin | Total bilirubin (continuous) | Clinical laboratory | .608 | .838 | No | No | Not selected | NA | P was not <0.10 and the variable was not judged to require clinical/literature-based retention in this round. |
| Chronic hepatitis B | Chronic hepatitis B | Etiology/clinical history | .834 | .600 | No | No | Not selected | NA | P was not <0.10 and the variable was not judged to require clinical/literature-based retention in this round. |
| Blood-pool enhancement pattern | blood_pool_enhancement_pattern | MRI enhancement | .364 | .467 | No | No | Not selected | NA | P was not <0.10 and the variable was not judged to require clinical/literature-based retention in this round. |
| Delayed central enhancement | delayed_central_enhancement | MRI enhancement | .235 | .697 | No | No | Not selected | NA | P was not <0.10 and the variable was not judged to require clinical/literature-based retention in this round. |
| Ascites | ascites | Other candidate variable | .955 | .919 | No | No | Not selected | NA | P was not <0.10 and the variable was not judged to require clinical/literature-based retention in this round. |
| Diffuse hepatic steatosis | diffuse_hepatic_steatosis | Other candidate variable | .555 | .946 | No | No | Not selected | NA | P was not <0.10 and the variable was not judged to require clinical/literature-based retention in this round. |
| Diffuse iron overload | diffuse_iron_overload | Other candidate variable | .579 | .582 | No | No | Not selected | NA | P was not <0.10 and the variable was not judged to require clinical/literature-based retention in this round. |
| Diffusion restriction | diffusion_restriction | Other candidate variable | .644 | .704 | No | No | Not selected | NA | P was not <0.10 and the variable was not judged to require clinical/literature-based retention in this round. |
| Focal marked T2 hyperintensity | focal_marked_t2_hyperintensity | Other candidate variable | .530 | .389 | No | No | Not selected | NA | P was not <0.10 and the variable was not judged to require clinical/literature-based retention in this round. |
| HBeAg positive | HBeAg positivity | Other candidate variable | .910 | .983 | No | No | Not selected | NA | P was not <0.10 and the variable was not judged to require clinical/literature-based retention in this round. |
| HBsAb positive | HBsAb positivity | Other candidate variable | .480 | .622 | No | No | Not selected | NA | P was not <0.10 and the variable was not judged to require clinical/literature-based retention in this round. |
| Imaging cirrhosis | imaging_cirrhosis | Other candidate variable | .987 | .926 | No | No | Not selected | NA | P was not <0.10 and the variable was not judged to require clinical/literature-based retention in this round. |
| Intratumoral fat | intratumoral_fat | Other candidate variable | .992 | .960 | No | No | Not selected | NA | P was not <0.10 and the variable was not judged to require clinical/literature-based retention in this round. |
| Intratumoral fat deficiency or internal nodule | intratumoral_fat_deficiency_or_internal_nodule | Other candidate variable | .874 | .780 | No | No | Not selected | NA | P was not <0.10 and the variable was not judged to require clinical/literature-based retention in this round. |
| Intratumoral iron deficiency or internal nodule | intratumoral_iron_deficiency_or_internal_nodule | Other candidate variable | .191 | .185 | No | No | Not selected | NA | P was not <0.10 and the variable was not judged to require clinical/literature-based retention in this round. |
| Intratumoral iron deposition greater than liver | intratumoral_iron_deposition_greater_than_liver | Other candidate variable | .738 | .944 | No | No | Not selected | NA | P was not <0.10 and the variable was not judged to require clinical/literature-based retention in this round. |
| Main portal vein width (cm) | main_portal_vein_width_cm | Other candidate variable | .807 | .730 | No | No | Not selected | NA | P was not <0.10 and the variable was not judged to require clinical/literature-based retention in this round. |
| Mild-to-moderate T2 hyperintensity | mild_moderate_t2_hyperintensity | Other candidate variable | .314 | .334 | No | No | Not selected | NA | P was not <0.10 and the variable was not judged to require clinical/literature-based retention in this round. |
| Portosystemic shunt/collaterals | portosystemic_shunt_or_collaterals | Other candidate variable | .865 | .940 | No | No | Not selected | NA | P was not <0.10 and the variable was not judged to require clinical/literature-based retention in this round. |
| Splenomegaly | splenomegaly | Other candidate variable | 1.000 | .854 | No | No | Not selected | NA | P was not <0.10 and the variable was not judged to require clinical/literature-based retention in this round. |
| Targetoid diffusion restriction | targetoid_diffusion_restriction | Other candidate variable | .781 | .947 | No | No | Not selected | NA | P was not <0.10 and the variable was not judged to require clinical/literature-based retention in this round. |
| Varices | varices | Other candidate variable | .603 | .734 | No | No | Not selected | NA | P was not <0.10 and the variable was not judged to require clinical/literature-based retention in this round. |

Literature Support Used for Clinical Retention

| **ID** | **Reference** | **URL** |
| --- | --- | --- |
| R1 | Jiang H et al. ASRAR model for recurrence after HCC resection. Radiology. 2023. | https://doi.org/10.1148/radiol.230527 |
| R2 | Cai Y et al. PIVKA-II and AFP secretion status and early recurrence after resection. Cancer Med. 2023. | https://doi.org/10.1002/cam4.6422 |
| R3 | Sun L et al. Gamma-GT, PIVKA-II and AFP after hepatectomy. Clin Exp Pharmacol Physiol. 2023. | https://doi.org/10.1111/1440-1681.13745 |
| R4 | Chan AWH et al. Pre- and post-operative models to predict early recurrence. J Hepatol. 2018. | https://doi.org/10.1016/j.jhep.2018.08.027 |
| R5 | Geng L et al. Prognostic role of preoperative ALBI grade: systematic review/meta-analysis. Eur J Gastroenterol Hepatol. 2020. | https://doi.org/10.1097/MEG.0000000000001618 |
| R6 | Ho SY et al. ALBI grade-based nomogram for tumor recurrence. Eur J Surg Oncol. 2019. | https://doi.org/10.1016/j.ejso.2018.10.541 |
| R7 | Qin L et al. Inflammation-based markers after liver resection for HCC. Biosci Trends. 2020. | https://doi.org/10.5582/bst.2020.03180 |
| R8 | Xu Q et al. Inflammation-based prognostic score after curative resection. J Immunol Res. 2018. | https://doi.org/10.1155/2018/4925498 |
| R9 | An C et al. Preoperative MR imaging to predict early recurrence after curative resection. Radiology. 2015. | https://doi.org/10.1148/radiol.15142394 |
| R10 | Ahn SJ et al. Gadoxetic acid-enhanced MRI features/texture and early recurrence. Abdom Radiol. 2019. | https://doi.org/10.1007/s00261-018-1768-9 |
| R11 | Wei Y et al. Preoperative MRI predicting early recurrence without MVI. Eur J Radiol. 2021. | https://doi.org/10.1016/j.ejrad.2021.109663 |
| R12 | Ziol M et al. Macrotrabecular-massive HCC subtype. Hepatology. 2018. | https://doi.org/10.1002/hep.29762 |
| R13 | Costentin C et al. Early recurrence scoring after HCC resection. Liver Int. 2023. | https://doi.org/10.1111/liv.15683 |

*Note. This table is a screening record rather than the final multivariable model. Variables retained here still require collinearity/redundancy checks, events-per-variable control, and final model selection in the next step.*

**Supplementary Table S6. Premodel variable pool.**

*Development cohort: 153 recurrence events; premodel pool limited to 15 one-parameter predictors*

Rules Used in Step 32

| **Item** | **Decision / rule** |
| --- | --- |
| Event-count basis | Use Cox regression event count: 153 recurrence events in the development cohort. |
| EPV rule | With 153 Cox events, a conservative EPV>=10 allows up to about 15 parameters; this premodel pool uses 15 one-parameter variables. |
| AFP decision | AFP is represented by AFP>400 ng/mL; log(AFP) is excluded from this premodel pool to avoid duplicate information. |
| NLR decision | Because NLR is typically skewed, log(NLR) is conditionally included; if it remains non-significant or contributes little, remove it before the main model. |
| Tumor number decision | Tumor number is excluded by design per user decision; tumor size uses >5 cm and, for multiple tumors, the maximum diameter. |
| Next step | Run redundancy/correlation and VIF checks, then reduce the model to a final 6-10 predictors before external validation. |

*Interpretation: this is not the final multivariable model. It is a deliberately restrained premodel pool. The next step should remove redundant variables through correlation/VIF checks and then shrink to the final Cox model.*

Recommended Premodel Pool

| **Order** | **Variable** | **Domain** | **Entry status** | **HR (95% CI)** | **Cox P** | **Parameter count** | **Reason** | **Next handling** |
| --- | --- | --- | --- | --- | --- | --- | --- | --- |
| 1 | AFP >400 ng/mL | Tumor biomarker | Direct premodel entry | 1.94 (1.40-2.68) | <.001 | 1 | AFP was represented as AFP >400 ng/mL rather than log(AFP), as prespecified; both univariable Cox and logistic analyses were statistically significant. | Entered the next step as the only AFP representation; log(AFP) was not entered simultaneously. |
| 2 | log(PIVKA-II) | Tumor biomarker | Direct premodel entry | 1.33 (1.25-1.42) | <.001 | 1 | PIVKA-II is an important serum marker related to HCC recurrence; univariable results were strongly positive. | Entered the collinearity check; if highly correlated with AFP or GGT, retention was based on clinical interpretability. |
| 3 | ALBI grade 2 or 3 | Liver function | Direct premodel entry | 1.38 (1.00-1.91) | .051 | 1 | Reflects hepatic functional reserve; Cox P was approximately 0.051, logistic P was <0.05, and the variable was supported by previous literature. | This binary variable used one parameter and was retained for multivariable screening. |
| 4 | log(NLR) | Inflammation marker | Conditional premodel entry | 1.12 (0.84-1.49) | .445 | 1 | Log(NLR) was preferred because NLR was skewed; although the univariable P value was >=0.10, inflammatory markers have literature support in recurrence research. | Initially entered the premodel pool; it would be excluded from the main model if nonsignificant or minimally contributory in multivariable or penalized screening. |
| 5 | Tumor size >5 cm | Tumor burden | Direct premodel entry | 2.94 (2.12-4.08) | <.001 | 1 | Tumor size was represented as >5 cm as prespecified; both univariable Cox and logistic analyses were strongly positive. | Retained as a core tumor-burden variable. |
| 6 | APHE proportion <50% | MRI enhancement | Direct premodel entry | 1.82 (1.31-2.51) | <.001 | 1 | Consistent with the reference model; univariable results were significant, and this variable was more suitable than nonrim/rim APHE as the main APHE expression in this study. | Used as the only prioritized APHE-related expression; nonrim APHE and rim APHE were not entered into the main premodel pool. |
| 7 | Satellite nodule | MRI invasion/spread | Direct premodel entry | 2.98 (2.05-4.32) | <.001 | 1 | Suggests intrahepatic spread or aggressiveness; univariable results were strongly positive. | Correlation with vessel invasion and peritumoral features was assessed. |
| 8 | Peritumoral hypointensity on portal venous phase images | MRI peritumoral feature | Direct premodel entry | 2.85 (2.02-4.01) | <.001 | 1 | Peritumoral hypointensity on portal venous phase images is an important MRI feature related to aggressiveness and recurrence; univariable results were strongly positive. | Redundancy with peritumoral T2 hyperintensity, satellite nodule, and vessel invasion was assessed. |
| 9 | Intrahepatic vessel invasion | MRI invasion/spread | Direct premodel entry | 3.23 (2.19-4.77) | <.001 | 1 | Imaging-visible vessel invasion is directly related to recurrence risk; univariable results were strongly positive. | If highly correlated with satellite nodule or peritumoral features, selection was based on AIC/C-index and clinical interpretability. |
| 10 | Incomplete capsule | MRI morphology | Direct premodel entry | 2.12 (1.46-3.08) | <.001 | 1 | Incomplete capsule is closer to an invasive margin than capsule presence/enhancement; univariable results were strongly positive. | Used as the prioritized capsule-related expression; other capsule variables were not entered simultaneously. |
| 11 | Necrosis or severe ischemia | MRI internal architecture | Direct premodel entry | 1.84 (1.34-2.53) | <.001 | 1 | Reflects intratumoral heterogeneity and aggressiveness; univariable results were significant. | Compared with internal-structure variables such as hemorrhage, multinodular architecture, and nodule-in-nodule architecture before final selection. |
| 12 | Infiltrative appearance | MRI morphology | Direct premodel entry | 2.50 (1.81-3.46) | <.001 | 1 | Infiltrative appearance suggests ill-defined margins and aggressive growth; univariable results were strongly positive. | Correlation with morphologic invasion variables such as incomplete capsule and satellite nodule was assessed. |
| 13 | Peritumoral T2 hyperintensity | MRI peritumoral feature | Direct premodel entry | 2.51 (1.81-3.49) | <.001 | 1 | Peritumoral T2 hyperintensity suggests peritumoral microenvironmental change; univariable results were strongly positive. | If highly correlated with peritumoral hypointensity on portal venous phase images, one variable was selected later. |
| 14 | Multiseptated or multinodular architecture | MRI internal architecture | Direct premodel entry | 2.24 (1.62-3.09) | <.001 | 1 | Reflects structural tumor heterogeneity; univariable results were strongly positive. | Compared with internal-structure variables such as nodule-in-nodule architecture, necrosis/ischemia, and hemorrhage before final selection. |
| 15 | log(GGT) | Clinical laboratory | Reserve candidate | 1.45 (1.23-1.72) | <.001 | 1 | Univariable results were significant and may reflect tumor-related cholestasis or hepatobiliary injury, but clinical priority was lower than AFP, PIVKA-II, and ALBI. | Retained as the 15th candidate variable; it could be removed preferentially if collinearity or model complexity was excessive. |

Variables Deliberately Not Entered at This Step

| **Variable** | **source_variable** | **Cox P** | **Logistic P** | **Reason for not entering premodel pool** |
| --- | --- | --- | --- | --- |
| log(AFP) | log_AFP | <.001 | .004 | Duplicated the prespecified AFP >400 ng/mL expression and was not entered into the premodel pool in this round. |
| NLR | NLR | .347 | .229 | If NLR was markedly skewed, log(NLR) was preferred and raw NLR was not entered simultaneously. |
| Nonrim APHE | nonrim_aphe | .324 | .528 | APHE <50% was prioritized among APHE-related variables; this variable was not entered at this stage. |
| Rim APHE | rim_aphe | .583 | .592 | APHE <50% was prioritized among APHE-related variables; this variable was not entered at this stage. |
| Capsule present | capsule_present | .016 | .007 | Incomplete capsule was prioritized among capsule-related variables to avoid duplication. |
| Capsule enhancement | capsule_enhancement | .028 | .015 | Incomplete capsule was prioritized among capsule-related variables to avoid duplication. |
| Tumor number | tumor_number |  |  | Not analyzed as a candidate variable as prespecified; for multiple tumors, the largest lesion diameter was used for tumor size. |
| Postoperative adjuvant therapy | postoperative_adjuvant_therapy |  |  | Not used as a preoperative main-model variable; used only for subsequent sensitivity analysis. |

**Recommended upper bound: because this premodel pool already reaches the EPV≈10 boundary, do not add further variables before redundancy checks unless there is a strong prespecified clinical reason.**

**Supplementary Table S7. Collinearity and VIF check.**

*Development cohort only; thresholds: VIF>5 or |correlation coefficient|>0.7*

# 5 Methods Applied

Pairwise correlations were calculated according to variable type: Spearman correlation for continuous-continuous pairs, point-biserial correlation for continuous-binary pairs, and Phi coefficient for binary-binary pairs. VIF was calculated from the full 15-variable design matrix using complete cases. Signals requiring review were predefined as VIF>5 or |r|>0.7.

# 6 VIF Results

| **Variable** | **source_variable** | **Domain** | **VIF** | **VIF decision** | **Missing handling** |
| --- | --- | --- | --- | --- | --- |
| Tumor size >5 cm | tumor_size_gt5cm | Tumor burden | 1.83 | Acceptable | VIF calculated on complete cases, n=258 |
| Necrosis or severe ischemia | necrosis_or_severe_ischemia | MRI internal architecture | 1.63 | Acceptable | VIF calculated on complete cases, n=258 |
| log(PIVKA-II) | log_PIVKAII | Tumor biomarker | 1.48 | Acceptable | VIF calculated on complete cases, n=258 |
| Infiltrative appearance | infiltrative_appearance | MRI morphology | 1.44 | Acceptable | VIF calculated on complete cases, n=258 |
| Multiseptated or multinodular architecture | multiseptated_or_multinodular_architecture | MRI internal architecture | 1.41 | Acceptable | VIF calculated on complete cases, n=258 |
| Peritumoral T2 hyperintensity | peritumoral_t2_hyperintensity | MRI peritumoral feature | 1.40 | Acceptable | VIF calculated on complete cases, n=258 |
| Intrahepatic vessel invasion | intrahepatic_vessel_invasion | MRI invasion/spread | 1.40 | Acceptable | VIF calculated on complete cases, n=258 |
| log(GGT) | log_GGT | Clinical laboratory | 1.37 | Acceptable | VIF calculated on complete cases, n=258 |
| Peritumoral hypointensity on portal venous phase images | peritumoral_hypoenhancement_pvp | MRI peritumoral feature | 1.29 | Acceptable | VIF calculated on complete cases, n=258 |
| Incomplete capsule | incomplete_capsule | MRI morphology | 1.25 | Acceptable | VIF calculated on complete cases, n=258 |
| AFP >400 ng/mL | AFP >400 ng/mL | Tumor biomarker | 1.24 | Acceptable | VIF calculated on complete cases, n=258 |
| Satellite nodule | satellite_nodule | MRI invasion/spread | 1.23 | Acceptable | VIF calculated on complete cases, n=258 |
| APHE proportion <50% | aphe_lt50 | MRI enhancement | 1.18 | Acceptable | VIF calculated on complete cases, n=258 |
| ALBI grade 2 or 3 | ALBI grade 2 or 3 | Liver function | 1.13 | Acceptable | VIF calculated on complete cases, n=258 |
| log(NLR) | log_NLR | Inflammation marker | 1.09 | Acceptable | VIF calculated on complete cases, n=258 |

# 7 Pairs With |Correlation Coefficient| >0.7

| **Variable 1** | **Variable 2** | **Method** | **n** | **Correlation coefficient** | **Absolute correlation** | **Recommendation** |
| --- | --- | --- | --- | --- | --- | --- |
| None | None | NA | 258 | NA | NA | No pairwise correlation exceeded the prespecified threshold. |

# 8 Recommended Actions

| **Variable** | **source_variable** | **VIF** | **Collinearity signal** | **Recommended action** | **Priority rule** |
| --- | --- | --- | --- | --- | --- |
| Tumor size >5 cm | tumor_size_gt5cm | 1.83 | No high collinearity signal by VIF or pairwise correlation. | Retain for next step | If removal is needed, prefer retaining variables with stronger clinical interpretability, more stable univariate P values, and fewer missing values. |
| Necrosis or severe ischemia | necrosis_or_severe_ischemia | 1.63 | No high collinearity signal by VIF or pairwise correlation. | Retain for next step | If removal is needed, prefer retaining variables with stronger clinical interpretability, more stable univariate P values, and fewer missing values. |
| log(PIVKA-II) | log_PIVKAII | 1.48 | No high collinearity signal by VIF or pairwise correlation. | Retain for next step | If removal is needed, prefer retaining variables with stronger clinical interpretability, more stable univariate P values, and fewer missing values. |
| Infiltrative appearance | infiltrative_appearance | 1.44 | No high collinearity signal by VIF or pairwise correlation. | Retain for next step | If removal is needed, prefer retaining variables with stronger clinical interpretability, more stable univariate P values, and fewer missing values. |
| Multiseptated or multinodular architecture | multiseptated_or_multinodular_architecture | 1.41 | No high collinearity signal by VIF or pairwise correlation. | Retain for next step | If removal is needed, prefer retaining variables with stronger clinical interpretability, more stable univariate P values, and fewer missing values. |
| Peritumoral T2 hyperintensity | peritumoral_t2_hyperintensity | 1.40 | No high collinearity signal by VIF or pairwise correlation. | Retain for next step | If removal is needed, prefer retaining variables with stronger clinical interpretability, more stable univariate P values, and fewer missing values. |
| Intrahepatic vessel invasion | intrahepatic_vessel_invasion | 1.40 | No high collinearity signal by VIF or pairwise correlation. | Retain for next step | If removal is needed, prefer retaining variables with stronger clinical interpretability, more stable univariate P values, and fewer missing values. |
| log(GGT) | log_GGT | 1.37 | No high collinearity signal by VIF or pairwise correlation. | Retain for next step | If removal is needed, prefer retaining variables with stronger clinical interpretability, more stable univariate P values, and fewer missing values. |
| Peritumoral hypointensity on portal venous phase images | peritumoral_hypoenhancement_pvp | 1.29 | No high collinearity signal by VIF or pairwise correlation. | Retain for next step | If removal is needed, prefer retaining variables with stronger clinical interpretability, more stable univariate P values, and fewer missing values. |
| Incomplete capsule | incomplete_capsule | 1.25 | No high collinearity signal by VIF or pairwise correlation. | Retain for next step | If removal is needed, prefer retaining variables with stronger clinical interpretability, more stable univariate P values, and fewer missing values. |
| AFP >400 ng/mL | AFP >400 ng/mL | 1.24 | No high collinearity signal by VIF or pairwise correlation. | Retain for next step | If removal is needed, prefer retaining variables with stronger clinical interpretability, more stable univariate P values, and fewer missing values. |
| Satellite nodule | satellite_nodule | 1.23 | No high collinearity signal by VIF or pairwise correlation. | Retain for next step | If removal is needed, prefer retaining variables with stronger clinical interpretability, more stable univariate P values, and fewer missing values. |
| APHE proportion <50% | aphe_lt50 | 1.18 | No high collinearity signal by VIF or pairwise correlation. | Retain for next step | If removal is needed, prefer retaining variables with stronger clinical interpretability, more stable univariate P values, and fewer missing values. |
| ALBI grade 2 or 3 | ALBI grade 2 or 3 | 1.13 | No high collinearity signal by VIF or pairwise correlation. | Retain for next step | If removal is needed, prefer retaining variables with stronger clinical interpretability, more stable univariate P values, and fewer missing values. |
| log(NLR) | log_NLR | 1.09 | No high collinearity signal by VIF or pairwise correlation. | Retain for next step | If removal is needed, prefer retaining variables with stronger clinical interpretability, more stable univariate P values, and fewer missing values. |

# 9 Variable Type and Missingness

| **Variable** | **source_variable** | **Type used for correlation** | **Non-missing n** | **Missing n** | **Unique values** |
| --- | --- | --- | --- | --- | --- |
| AFP >400 ng/mL | AFP >400 ng/mL | Binary | 258 | 0 | 2 |
| log(PIVKA-II) | log_PIVKAII | Continuous | 258 | 0 | 258 |
| ALBI grade 2 or 3 | ALBI grade 2 or 3 | Binary | 258 | 0 | 2 |
| log(NLR) | log_NLR | Continuous | 258 | 0 | 255 |
| Tumor size >5 cm | tumor_size_gt5cm | Binary | 258 | 0 | 2 |
| APHE proportion <50% | aphe_lt50 | Binary | 258 | 0 | 2 |
| Satellite nodule | satellite_nodule | Binary | 258 | 0 | 2 |
| Peritumoral hypointensity on portal venous phase images | peritumoral_hypoenhancement_pvp | Binary | 258 | 0 | 2 |
| Intrahepatic vessel invasion | intrahepatic_vessel_invasion | Binary | 258 | 0 | 2 |
| Incomplete capsule | incomplete_capsule | Binary | 258 | 0 | 2 |
| Necrosis or severe ischemia | necrosis_or_severe_ischemia | Binary | 258 | 0 | 2 |
| Infiltrative appearance | infiltrative_appearance | Binary | 258 | 0 | 2 |
| Peritumoral T2 hyperintensity | peritumoral_t2_hyperintensity | Binary | 258 | 0 | 2 |
| Multiseptated or multinodular architecture | multiseptated_or_multinodular_architecture | Binary | 258 | 0 | 2 |
| log(GGT) | log_GGT | Continuous | 258 | 0 | 139 |

# 10 All Pairwise Correlations

| **Variable 1** | **Variable 2** | **Method** | **n** | **Correlation coefficient** | **Absolute correlation** | **\|r\| >0.7** |
| --- | --- | --- | --- | --- | --- | --- |
| Tumor size >5 cm | Necrosis or severe ischemia | Phi coefficient | 258 | 0.540 | 0.540 | No |
| Tumor size >5 cm | Multiseptated or multinodular architecture | Phi coefficient | 258 | 0.437 | 0.437 | No |
| log(PIVKA-II) | Tumor size >5 cm | Point-biserial correlation | 258 | 0.430 | 0.430 | No |
| Infiltrative appearance | Peritumoral T2 hyperintensity | Phi coefficient | 258 | 0.392 | 0.392 | No |
| Peritumoral hypointensity on portal venous phase images | Peritumoral T2 hyperintensity | Phi coefficient | 258 | 0.388 | 0.388 | No |
| Necrosis or severe ischemia | Multiseptated or multinodular architecture | Phi coefficient | 258 | 0.383 | 0.383 | No |
| log(PIVKA-II) | Multiseptated or multinodular architecture | Point-biserial correlation | 258 | 0.363 | 0.363 | No |
| Tumor size >5 cm | log(GGT) | Point-biserial correlation | 258 | 0.358 | 0.358 | No |
| log(PIVKA-II) | Necrosis or severe ischemia | Point-biserial correlation | 258 | 0.356 | 0.356 | No |
| Intrahepatic vessel invasion | Infiltrative appearance | Phi coefficient | 258 | 0.340 | 0.340 | No |
| Tumor size >5 cm | Intrahepatic vessel invasion | Phi coefficient | 258 | 0.329 | 0.329 | No |
| Satellite nodule | Intrahepatic vessel invasion | Phi coefficient | 258 | 0.327 | 0.327 | No |
| log(PIVKA-II) | log(GGT) | Spearman correlation | 258 | 0.321 | 0.321 | No |
| Intrahepatic vessel invasion | log(GGT) | Point-biserial correlation | 258 | 0.317 | 0.317 | No |
| Intrahepatic vessel invasion | Necrosis or severe ischemia | Phi coefficient | 258 | 0.304 | 0.304 | No |
| AFP >400 ng/mL | log(PIVKA-II) | Point-biserial correlation | 258 | 0.303 | 0.303 | No |
| Incomplete capsule | Infiltrative appearance | Phi coefficient | 258 | 0.301 | 0.301 | No |
| Incomplete capsule | Necrosis or severe ischemia | Phi coefficient | 258 | 0.298 | 0.298 | No |
| log(PIVKA-II) | Intrahepatic vessel invasion | Point-biserial correlation | 258 | 0.292 | 0.292 | No |
| AFP >400 ng/mL | Intrahepatic vessel invasion | Phi coefficient | 258 | 0.288 | 0.288 | No |
| Satellite nodule | Infiltrative appearance | Phi coefficient | 258 | 0.287 | 0.287 | No |
| APHE proportion <50% | Necrosis or severe ischemia | Phi coefficient | 258 | 0.286 | 0.286 | No |
| AFP >400 ng/mL | Tumor size >5 cm | Phi coefficient | 258 | 0.284 | 0.284 | No |
| Peritumoral hypointensity on portal venous phase images | Incomplete capsule | Phi coefficient | 258 | 0.276 | 0.276 | No |
| ALBI grade 2 or 3 | log(GGT) | Point-biserial correlation | 258 | 0.266 | 0.266 | No |
| Satellite nodule | Necrosis or severe ischemia | Phi coefficient | 258 | 0.263 | 0.263 | No |
| Infiltrative appearance | Multiseptated or multinodular architecture | Phi coefficient | 258 | 0.262 | 0.262 | No |
| Peritumoral T2 hyperintensity | Multiseptated or multinodular architecture | Phi coefficient | 258 | 0.262 | 0.262 | No |
| Necrosis or severe ischemia | Infiltrative appearance | Phi coefficient | 258 | 0.261 | 0.261 | No |
| log(PIVKA-II) | Satellite nodule | Point-biserial correlation | 258 | 0.260 | 0.260 | No |
| Tumor size >5 cm | Incomplete capsule | Phi coefficient | 258 | 0.260 | 0.260 | No |
| Tumor size >5 cm | Peritumoral hypointensity on portal venous phase images | Phi coefficient | 258 | 0.253 | 0.253 | No |
| Tumor size >5 cm | Peritumoral T2 hyperintensity | Phi coefficient | 258 | 0.252 | 0.252 | No |
| Tumor size >5 cm | Infiltrative appearance | Phi coefficient | 258 | 0.250 | 0.250 | No |
| Intrahepatic vessel invasion | Multiseptated or multinodular architecture | Phi coefficient | 258 | 0.249 | 0.249 | No |
| Multiseptated or multinodular architecture | log(GGT) | Point-biserial correlation | 258 | 0.248 | 0.248 | No |
| AFP >400 ng/mL | Multiseptated or multinodular architecture | Phi coefficient | 258 | 0.245 | 0.245 | No |
| Tumor size >5 cm | APHE proportion <50% | Phi coefficient | 258 | 0.242 | 0.242 | No |
| Infiltrative appearance | log(GGT) | Point-biserial correlation | 258 | 0.237 | 0.237 | No |
| AFP >400 ng/mL | APHE proportion <50% | Phi coefficient | 258 | 0.235 | 0.235 | No |
| log(PIVKA-II) | APHE proportion <50% | Point-biserial correlation | 258 | 0.234 | 0.234 | No |
| Necrosis or severe ischemia | Peritumoral T2 hyperintensity | Phi coefficient | 258 | 0.230 | 0.230 | No |
| Tumor size >5 cm | Satellite nodule | Phi coefficient | 258 | 0.225 | 0.225 | No |
| log(PIVKA-II) | Peritumoral T2 hyperintensity | Point-biserial correlation | 258 | 0.224 | 0.224 | No |
| Peritumoral hypointensity on portal venous phase images | Infiltrative appearance | Phi coefficient | 258 | 0.221 | 0.221 | No |
| Necrosis or severe ischemia | log(GGT) | Point-biserial correlation | 258 | 0.217 | 0.217 | No |
| log(PIVKA-II) | Infiltrative appearance | Point-biserial correlation | 258 | 0.216 | 0.216 | No |
| AFP >400 ng/mL | Necrosis or severe ischemia | Phi coefficient | 258 | 0.212 | 0.212 | No |
| Satellite nodule | Multiseptated or multinodular architecture | Phi coefficient | 258 | 0.212 | 0.212 | No |
| Peritumoral hypointensity on portal venous phase images | Multiseptated or multinodular architecture | Phi coefficient | 258 | 0.205 | 0.205 | No |
| Incomplete capsule | Multiseptated or multinodular architecture | Phi coefficient | 258 | 0.200 | 0.200 | No |
| Satellite nodule | Peritumoral T2 hyperintensity | Phi coefficient | 258 | 0.186 | 0.186 | No |
| APHE proportion <50% | Satellite nodule | Phi coefficient | 258 | 0.182 | 0.182 | No |
| APHE proportion <50% | Peritumoral T2 hyperintensity | Phi coefficient | 258 | 0.181 | 0.181 | No |
| ALBI grade 2 or 3 | log(NLR) | Point-biserial correlation | 258 | 0.181 | 0.181 | No |
| AFP >400 ng/mL | Satellite nodule | Phi coefficient | 258 | 0.173 | 0.173 | No |
| Satellite nodule | log(GGT) | Point-biserial correlation | 258 | 0.173 | 0.173 | No |
| APHE proportion <50% | Multiseptated or multinodular architecture | Phi coefficient | 258 | 0.171 | 0.171 | No |
| Incomplete capsule | Peritumoral T2 hyperintensity | Phi coefficient | 258 | 0.168 | 0.168 | No |
| Intrahepatic vessel invasion | Incomplete capsule | Phi coefficient | 258 | 0.164 | 0.164 | No |
| AFP >400 ng/mL | Peritumoral T2 hyperintensity | Phi coefficient | 258 | 0.162 | 0.162 | No |
| Intrahepatic vessel invasion | Peritumoral T2 hyperintensity | Phi coefficient | 258 | 0.159 | 0.159 | No |
| Peritumoral hypointensity on portal venous phase images | Necrosis or severe ischemia | Phi coefficient | 258 | 0.154 | 0.154 | No |
| log(PIVKA-II) | ALBI grade 2 or 3 | Point-biserial correlation | 258 | 0.149 | 0.149 | No |
| log(NLR) | Multiseptated or multinodular architecture | Point-biserial correlation | 258 | 0.144 | 0.144 | No |
| log(NLR) | Tumor size >5 cm | Point-biserial correlation | 258 | 0.143 | 0.143 | No |
| AFP >400 ng/mL | Peritumoral hypointensity on portal venous phase images | Phi coefficient | 258 | 0.141 | 0.141 | No |
| AFP >400 ng/mL | Infiltrative appearance | Phi coefficient | 258 | 0.141 | 0.141 | No |
| log(PIVKA-II) | Peritumoral hypointensity on portal venous phase images | Point-biserial correlation | 258 | 0.140 | 0.140 | No |
| APHE proportion <50% | Intrahepatic vessel invasion | Phi coefficient | 258 | 0.138 | 0.138 | No |
| Peritumoral T2 hyperintensity | log(GGT) | Point-biserial correlation | 258 | 0.135 | 0.135 | No |
| log(PIVKA-II) | Incomplete capsule | Point-biserial correlation | 258 | 0.130 | 0.130 | No |
| log(NLR) | APHE proportion <50% | Point-biserial correlation | 258 | 0.127 | 0.127 | No |
| APHE proportion <50% | Peritumoral hypointensity on portal venous phase images | Phi coefficient | 258 | 0.126 | 0.126 | No |
| Peritumoral hypointensity on portal venous phase images | Intrahepatic vessel invasion | Phi coefficient | 258 | 0.119 | 0.119 | No |
| APHE proportion <50% | log(GGT) | Point-biserial correlation | 258 | 0.109 | 0.109 | No |
| Satellite nodule | Incomplete capsule | Phi coefficient | 258 | 0.105 | 0.105 | No |
| APHE proportion <50% | Infiltrative appearance | Phi coefficient | 258 | 0.104 | 0.104 | No |
| APHE proportion <50% | Incomplete capsule | Phi coefficient | 258 | 0.104 | 0.104 | No |
| AFP >400 ng/mL | log(GGT) | Point-biserial correlation | 258 | 0.096 | 0.096 | No |
| ALBI grade 2 or 3 | Peritumoral T2 hyperintensity | Phi coefficient | 258 | 0.092 | 0.092 | No |
| ALBI grade 2 or 3 | Tumor size >5 cm | Phi coefficient | 258 | 0.087 | 0.087 | No |
| ALBI grade 2 or 3 | Multiseptated or multinodular architecture | Phi coefficient | 258 | 0.082 | 0.082 | No |
| ALBI grade 2 or 3 | Intrahepatic vessel invasion | Phi coefficient | 258 | 0.081 | 0.081 | No |
| log(NLR) | Incomplete capsule | Point-biserial correlation | 258 | 0.074 | 0.074 | No |
| log(NLR) | Peritumoral T2 hyperintensity | Point-biserial correlation | 258 | 0.068 | 0.068 | No |
| log(NLR) | Intrahepatic vessel invasion | Point-biserial correlation | 258 | 0.068 | 0.068 | No |
| Incomplete capsule | log(GGT) | Point-biserial correlation | 258 | 0.065 | 0.065 | No |
| Satellite nodule | Peritumoral hypointensity on portal venous phase images | Phi coefficient | 258 | 0.061 | 0.061 | No |
| log(NLR) | Necrosis or severe ischemia | Point-biserial correlation | 258 | 0.061 | 0.061 | No |
| ALBI grade 2 or 3 | Infiltrative appearance | Phi coefficient | 258 | 0.052 | 0.052 | No |
| log(NLR) | Peritumoral hypointensity on portal venous phase images | Point-biserial correlation | 258 | 0.050 | 0.050 | No |
| AFP >400 ng/mL | log(NLR) | Point-biserial correlation | 258 | 0.049 | 0.049 | No |
| Peritumoral hypointensity on portal venous phase images | log(GGT) | Point-biserial correlation | 258 | 0.045 | 0.045 | No |
| ALBI grade 2 or 3 | Satellite nodule | Phi coefficient | 258 | 0.039 | 0.039 | No |
| log(NLR) | log(GGT) | Spearman correlation | 258 | 0.035 | 0.035 | No |
| AFP >400 ng/mL | Incomplete capsule | Phi coefficient | 258 | 0.035 | 0.035 | No |
| ALBI grade 2 or 3 | Incomplete capsule | Phi coefficient | 258 | 0.035 | 0.035 | No |
| ALBI grade 2 or 3 | Necrosis or severe ischemia | Phi coefficient | 258 | 0.032 | 0.032 | No |
| log(NLR) | Satellite nodule | Point-biserial correlation | 258 | 0.025 | 0.025 | No |
| ALBI grade 2 or 3 | APHE proportion <50% | Phi coefficient | 258 | 0.023 | 0.023 | No |
| log(NLR) | Infiltrative appearance | Point-biserial correlation | 258 | 0.018 | 0.018 | No |
| ALBI grade 2 or 3 | Peritumoral hypointensity on portal venous phase images | Phi coefficient | 258 | -0.006 | 0.006 | No |
| AFP >400 ng/mL | ALBI grade 2 or 3 | Phi coefficient | 258 | 0.006 | 0.006 | No |
| log(PIVKA-II) | log(NLR) | Spearman correlation | 258 | 0.001 | 0.001 | No |

*Note. This report identifies redundancy risk only. Final removal should also consider clinical interpretability, univariate Cox stability, missingness, and external validation performance.*

**Supplementary Table S8. Clinical and MRI Cox model details.**

Clinical Cox model

| Item | | | | | | | | | Description | | | | | | | | |
| --- | --- | --- | --- | --- | --- | --- | --- | --- | --- | --- | --- | --- | --- | --- | --- | --- | --- |
| Data | | | | | | | | | Development cohort only; n=258, early recurrence events=153. | | | | | | | | |
| Outcome | | | | | | | | | time = min(disease-free survival, 24 months); event = early recurrence <24 months. | | | | | | | | |
| Initial model | | | | | | | | | AFP>400 + log(PIVKA-II) + ALBI grade 2/3 + log(NLR) + log(GGT). | | | | | | | | |
| Simplification rule | | | | | | | | | log(NLR) was prespecified as conditional; because it was not significant in the initial multivariable model and the simplified model had lower AIC, it was removed from the recommended final clinical model. | | | | | | | | |
| Recommended final clinical model | | | | | | | | | AFP>400 + log(PIVKA-II) + ALBI grade 2/3 + log(GGT). | | | | | | | | |
| Model | | Variable | | | Unit / comparison | | | Beta | | HR (95% CI) | | | P value | | | Decision | |
| Initial clinical model | | AFP >400 ng/mL | | | 1 vs 0 | | | 0.380 | | 1.46 (1.04-2.05) | | | .027 | | | Retain in final model | |
| Initial clinical model | | log(PIVKA-II) | | | per 1-unit increase | | | 0.250 | | 1.28 (1.20-1.38) | | | <.001 | | | Retain in final model | |
| Initial clinical model | | ALBI grade 2 or 3 | | | 1 vs 0 | | | 0.018 | | 1.02 (0.71-1.45) | | | .921 | | | Retain in final model | |
| Initial clinical model | | log(NLR) | | | per 1-unit increase | | | 0.085 | | 1.09 (0.82-1.44) | | | .554 | | | Remove from recommended final model | |
| Initial clinical model | | log(GGT) | | | per 1-unit increase | | | 0.177 | | 1.19 (0.99-1.44) | | | .064 | | | Retain in final model | |
| Recommended final clinical model | | AFP >400 ng/mL | | | 1 vs 0 | | | 0.371 | | 1.45 (1.04-2.03) | | | .031 | | | Included in recommended final model | |
| Recommended final clinical model | | log(PIVKA-II) | | | per 1-unit increase | | | 0.250 | | 1.28 (1.20-1.38) | | | <.001 | | | Included in recommended final model | |
| Recommended final clinical model | | ALBI grade 2 or 3 | | | 1 vs 0 | | | 0.045 | | 1.05 (0.74-1.47) | | | .797 | | | Included in recommended final model | |
| Recommended final clinical model | | log(GGT) | | | per 1-unit increase | | | 0.178 | | 1.20 (0.99-1.44) | | | .060 | | | Included in recommended final model | |
| Model | N | | | Events | | Parameters | | | AIC | | | C-index | | C-index SE | | | Log-likelihood |
| Initial clinical model | 258 | | | 153 | | 5 | | | 1508.32 | | | 0.711 | | 0.021 | | | -749.16 |
| Recommended final clinical model | 258 | | | 153 | | 4 | | | 1506.66 | | | 0.710 | | 0.020 | | | -749.33 |
| Model | | | Variable | | | | Chi-square | | | | P value | | | | PH interpretation | | |
| Initial clinical model | | | AFP_gt400 | | | | 7.789 | | | | .005 | | | | Possible PH violation | | |
| Initial clinical model | | | log_PIVKAII | | | | 0.373 | | | | .541 | | | | No evidence of PH violation | | |
| Initial clinical model | | | ALBI_2or3 | | | | 4.178 | | | | .041 | | | | Possible PH violation | | |
| Initial clinical model | | | log_NLR | | | | 0.043 | | | | .837 | | | | No evidence of PH violation | | |
| Initial clinical model | | | log_GGT | | | | 0.348 | | | | .555 | | | | No evidence of PH violation | | |
| Initial clinical model | | | GLOBAL | | | | 12.813 | | | | .025 | | | | Possible PH violation | | |
| Recommended final clinical model | | | AFP_gt400 | | | | 7.746 | | | | .005 | | | | Possible PH violation | | |
| Recommended final clinical model | | | log_PIVKAII | | | | 0.366 | | | | .545 | | | | No evidence of PH violation | | |
| Recommended final clinical model | | | ALBI_2or3 | | | | 4.174 | | | | .041 | | | | Possible PH violation | | |
| Recommended final clinical model | | | log_GGT | | | | 0.332 | | | | .565 | | | | No evidence of PH violation | | |
| Recommended final clinical model | | | GLOBAL | | | | 12.469 | | | | .014 | | | | Possible PH violation | | |

MRI Cox model

| Item | | | | | | | | | Description | | | | | | | | |
| --- | --- | --- | --- | --- | --- | --- | --- | --- | --- | --- | --- | --- | --- | --- | --- | --- | --- |
| Data | | | | | | | | | Development cohort only; n=258, early recurrence events=153. | | | | | | | | |
| Outcome | | | | | | | | | time = min(disease-free survival, 24 months); event = early recurrence <24 months. | | | | | | | | |
| Initial MRI model | | | | | | | | | All 10 MRI variables retained after Step 34 were entered into an initial multivariable Cox model. | | | | | | | | |
| Selection method | | | | | | | | | Backward AIC selection was applied to suggest a parsimonious MRI model while preserving the Cox framework. | | | | | | | | |
| Collinearity prerequisite | | | | | | | | | Step 33 showed no VIF>5 and no pairwise \|r\|>0.7 among the 15 premodel variables; therefore all MRI variables were eligible for the initial MRI model. | | | | | | | | |
| Model | | Variable | | | MRI domain | | | Beta | | HR (95% CI) | | | P value | | | Decision | |
| Initial full MRI model | | Tumor size >5 cm | | | Tumor burden | | | 0.666 | | 1.95 (1.31-2.88) | | | <.001 | | | Retained in recommended MRI model | |
| Initial full MRI model | | APHE proportion <50% | | | Enhancement pattern | | | 0.206 | | 1.23 (0.87-1.74) | | | .243 | | | Removed by backward AIC | |
| Initial full MRI model | | Satellite nodule | | | Invasion/spread | | | 0.790 | | 2.20 (1.47-3.30) | | | <.001 | | | Retained in recommended MRI model | |
| Initial full MRI model | | Peritumoral hypointensity on portal venous phase images | | | Peritumoral feature | | | 0.579 | | 1.78 (1.22-2.61) | | | .003 | | | Retained in recommended MRI model | |
| Initial full MRI model | | Intrahepatic vessel invasion | | | Invasion/spread | | | 0.509 | | 1.66 (1.06-2.62) | | | .028 | | | Retained in recommended MRI model | |
| Initial full MRI model | | Incomplete capsule | | | Tumor margin/capsule | | | 0.314 | | 1.37 (0.90-2.08) | | | .143 | | | Retained in recommended MRI model | |
| Initial full MRI model | | Necrosis or severe ischemia | | | Internal architecture | | | -0.253 | | 0.78 (0.52-1.15) | | | .210 | | | Removed by backward AIC | |
| Initial full MRI model | | Infiltrative appearance | | | Tumor morphology | | | 0.204 | | 1.23 (0.82-1.84) | | | .322 | | | Removed by backward AIC | |
| Initial full MRI model | | Peritumoral T2 hyperintensity | | | Peritumoral feature | | | 0.232 | | 1.26 (0.85-1.87) | | | .249 | | | Retained in recommended MRI model | |
| Initial full MRI model | | Multiseptated or multinodular architecture | | | Internal architecture | | | 0.357 | | 1.43 (1.00-2.05) | | | .053 | | | Retained in recommended MRI model | |
| Recommended MRI model by backward AIC | | Tumor size >5 cm | | | Tumor burden | | | 0.589 | | 1.80 (1.25-2.60) | | | .002 | | | Retained in recommended MRI model | |
| Recommended MRI model by backward AIC | | Satellite nodule | | | Invasion/spread | | | 0.824 | | 2.28 (1.54-3.37) | | | <.001 | | | Retained in recommended MRI model | |
| Recommended MRI model by backward AIC | | Peritumoral hypointensity on portal venous phase images | | | Peritumoral feature | | | 0.609 | | 1.84 (1.26-2.68) | | | .002 | | | Retained in recommended MRI model | |
| Recommended MRI model by backward AIC | | Intrahepatic vessel invasion | | | Invasion/spread | | | 0.525 | | 1.69 (1.10-2.59) | | | .016 | | | Retained in recommended MRI model | |
| Recommended MRI model by backward AIC | | Incomplete capsule | | | Tumor margin/capsule | | | 0.312 | | 1.37 (0.92-2.03) | | | .123 | | | Retained in recommended MRI model | |
| Recommended MRI model by backward AIC | | Peritumoral T2 hyperintensity | | | Peritumoral feature | | | 0.309 | | 1.36 (0.94-1.98) | | | .107 | | | Retained in recommended MRI model | |
| Recommended MRI model by backward AIC | | Multiseptated or multinodular architecture | | | Internal architecture | | | 0.362 | | 1.44 (1.01-2.04) | | | .044 | | | Retained in recommended MRI model | |
| Model | N | | | Events | | Parameters | | | AIC | | | C-index | | C-index SE | | | Log-likelihood |
| Initial full MRI model | 258 | | | 153 | | 10 | | | 1498.45 | | | 0.725 | | 0.020 | | | -739.23 |
| Recommended MRI model by backward AIC | 258 | | | 153 | | 7 | | | 1495.88 | | | 0.716 | | 0.021 | | | -740.94 |
| item | | | | | | | | | value | | | | | | | | |
| Initial formula | | | | | | | | | Surv(time_24, event_24) ~ tumor_size_gt5cm + aphe_lt50 + satellite_nodule + peritumoral_hypo_pvp + intrahepatic_vessel_invasion + incomplete_capsule + necrosis_or_severe_ischemia + infiltrative_appearance + peritumoral_t2_hyperintensity + multiseptated_multinodular | | | | | | | | |
| Recommended formula | | | | | | | | | Surv(time_24, event_24) ~ tumor_size_gt5cm + satellite_nodule + peritumoral_hypo_pvp + intrahepatic_vessel_invasion + incomplete_capsule + peritumoral_t2_hyperintensity + multiseptated_multinodular | | | | | | | | |
| Variables dropped by backward AIC | | | | | | | | | aphe_lt50, necrosis_or_severe_ischemia, infiltrative_appearance | | | | | | | | |
| Variables retained by backward AIC | | | | | | | | | tumor_size_gt5cm, satellite_nodule, peritumoral_hypo_pvp, intrahepatic_vessel_invasion, incomplete_capsule, peritumoral_t2_hyperintensity, multiseptated_multinodular | | | | | | | | |
| Model | | | Variable | | | | Chi-square | | | | P value | | | | PH interpretation | | |
| Initial full MRI model | | | tumor_size_gt5cm | | | | 0.317 | | | | .574 | | | | No evidence of PH violation | | |
| Initial full MRI model | | | aphe_lt50 | | | | 2.780 | | | | .095 | | | | No evidence of PH violation | | |
| Initial full MRI model | | | satellite_nodule | | | | 0.130 | | | | .719 | | | | No evidence of PH violation | | |
| Initial full MRI model | | | peritumoral_hypo_pvp | | | | 8.917 | | | | .003 | | | | Possible PH violation | | |
| Initial full MRI model | | | intrahepatic_vessel_invasion | | | | 3.358 | | | | .067 | | | | No evidence of PH violation | | |
| Initial full MRI model | | | incomplete_capsule | | | | 1.749 | | | | .186 | | | | No evidence of PH violation | | |
| Initial full MRI model | | | necrosis_or_severe_ischemia | | | | 5.258 | | | | .022 | | | | Possible PH violation | | |
| Initial full MRI model | | | infiltrative_appearance | | | | 0.051 | | | | .820 | | | | No evidence of PH violation | | |
| Initial full MRI model | | | peritumoral_t2_hyperintensity | | | | 2.770 | | | | .096 | | | | No evidence of PH violation | | |
| Initial full MRI model | | | multiseptated_multinodular | | | | 4.184 | | | | .041 | | | | Possible PH violation | | |
| Initial full MRI model | | | GLOBAL | | | | 31.687 | | | | <.001 | | | | Possible PH violation | | |
| Recommended MRI model by backward AIC | | | tumor_size_gt5cm | | | | 0.277 | | | | .599 | | | | No evidence of PH violation | | |
| Recommended MRI model by backward AIC | | | satellite_nodule | | | | 0.061 | | | | .806 | | | | No evidence of PH violation | | |
| Recommended MRI model by backward AIC | | | peritumoral_hypo_pvp | | | | 8.614 | | | | .003 | | | | Possible PH violation | | |
| Recommended MRI model by backward AIC | | | intrahepatic_vessel_invasion | | | | 3.773 | | | | .052 | | | | No evidence of PH violation | | |
| Recommended MRI model by backward AIC | | | incomplete_capsule | | | | 1.889 | | | | .169 | | | | No evidence of PH violation | | |
| Recommended MRI model by backward AIC | | | peritumoral_t2_hyperintensity | | | | 2.948 | | | | .086 | | | | No evidence of PH violation | | |
| Recommended MRI model by backward AIC | | | multiseptated_multinodular | | | | 4.074 | | | | .044 | | | | Possible PH violation | | |
| Recommended MRI model by backward AIC | | | GLOBAL | | | | 19.860 | | | | .006 | | | | Possible PH violation | | |

Note. This supplementary table combines the separately generated clinical and MRI Cox model detail tables without changing the underlying estimates.

**Supplementary Table S9. Adjuvant therapy sensitivity analysis.**

*Robustness of the final combined model and risk stratification*

# 11 Methods

| **Item** | **Description** |
| --- | --- |
| Model adjustment | The final combined Cox model was refitted in the development cohort with postoperative adjuvant therapy added as an additional covariate. |
| Predictor stability | Hazard ratios of the six final predictors before and after adjuvant adjustment were compared; HR change <15% was considered materially stable. |
| Risk stratification | The original final model risk score and the fixed cutoff of 0.584 were used; the cutoff was not re-estimated. |
| Subgroup/exclusion checks | Risk stratification was examined within adjuvant therapy strata and after excluding patients who received adjuvant therapy. |

# 12 Adjuvant Therapy Distribution

| **Cohort** | **Adjuvant therapy** | **N** |
| --- | --- | --- |
| Development cohort | No | 127 |
| Development cohort | Yes | 131 |
| External validation cohort | No | 10 |
| External validation cohort | Yes | 52 |

# 13 Model Adjustment for Adjuvant Therapy

| **Model** | **N** | **Events** | **C-index** | **AIC** | **Adjuvant therapy HR (95% CI)** | **Adjuvant therapy P** |
| --- | --- | --- | --- | --- | --- | --- |
| Final combined model | 258 | 153 | 0.756 | 1464.8 |  |  |
| Final combined model + adjuvant therapy | 258 | 153 | 0.757 | 1466.7 | 1.060 (0.768-1.463) | 0.723 |

# 14 Predictor Stability After Adjuvant Adjustment

| **Variable** | **Original HR** | **Adjusted HR** | **HR percent change** | **Stable (<15%)** |
| --- | --- | --- | --- | --- |
| incomplete_capsule | 1.422 | 1.416 | 0.4% | Yes |
| intrahepatic_vessel_invasion | 1.759 | 1.751 | 0.5% | Yes |
| log_PIVKAII | 1.226 | 1.226 | 0.0% | Yes |
| peritumoral_hypo_pvp | 1.832 | 1.830 | 0.1% | Yes |
| satellite_nodule | 2.330 | 2.313 | 0.7% | Yes |
| tumor_size_gt5cm | 1.425 | 1.426 | 0.1% | Yes |

# 15 Risk Stratification Sensitivity Checks

| **Analysis** | **Cohort** | **Risk group** | **N** | **Early recurrence, n (%)** | **HR (95% CI)** | **Cox P** | **Log-rank P** |
| --- | --- | --- | --- | --- | --- | --- | --- |
| No adjuvant therapy subgroup | Development cohort | Low risk | 71 | 19 (26.8%) | Reference |  | <0.001 |
| No adjuvant therapy subgroup | Development cohort | High risk | 56 | 50 (89.3%) | 7.179 (4.157-12.399) | <0.001 | <0.001 |
| Adjuvant therapy subgroup | Development cohort | Low risk | 62 | 25 (40.3%) | Reference |  | <0.001 |
| Adjuvant therapy subgroup | Development cohort | High risk | 69 | 59 (85.5%) | 4.128 (2.555-6.670) | <0.001 | <0.001 |
| Excluding patients with adjuvant therapy | Development cohort | Low risk | 71 | 19 (26.8%) | Reference |  | <0.001 |
| Excluding patients with adjuvant therapy | Development cohort | High risk | 56 | 50 (89.3%) | 7.179 (4.157-12.399) | <0.001 | <0.001 |
| No adjuvant therapy subgroup | External validation cohort | Low risk | 3 | 0 (0.0%) | Reference |  | 0.009 |
| No adjuvant therapy subgroup | External validation cohort | High risk | 7 | 7 (100.0%) | Not estimable | NA | 0.009 |
| Adjuvant therapy subgroup | External validation cohort | Low risk | 26 | 1 (3.8%) | Reference |  | <0.001 |
| Adjuvant therapy subgroup | External validation cohort | High risk | 26 | 22 (84.6%) | 48.168 (6.409-361.991) | <0.001 | <0.001 |
| Excluding patients with adjuvant therapy | External validation cohort | Low risk | 3 | 0 (0.0%) | Reference |  | 0.009 |
| Excluding patients with adjuvant therapy | External validation cohort | High risk | 7 | 7 (100.0%) | Not estimable | NA | 0.009 |

*Note. Postoperative adjuvant therapy was used only for sensitivity analysis because the prediction model is intended for preoperative risk estimation. Subgroup estimates, especially in the external no-adjuvant subgroup, should be interpreted cautiously because of limited sample size.*

**Supplementary Table S10. MRI acquisition parameters.**

The same liver MRI acquisition protocol was used in the development cohort and the external validation cohort. All examinations were performed with a Siemens MAGNETOM Skyra 3.0T MRI scanner. Patients fasted for 4-6 hours, were positioned supine and head first, and were trained for regular breathing and end-expiratory breath-holding when required. Dynamic contrast-enhanced MRI was performed after antecubital venous injection of the extracellular gadolinium-based contrast agent gadopentetate dimeglumine (Gd-DTPA; Magnevist, Bayer) at a dose of 0.1 mmol/kg and an injection rate of 2-3 mL/s, followed by a 20-mL saline flush. Arterial and portal venous phase images were acquired approximately 20-35 s and 60-70 s after the start of contrast injection, respectively.

| **Parameter** | **T1WI** | **FS-T2WI** | **DWI** | **Arterial phase** | **Portal venous phase** |
| --- | --- | --- | --- | --- | --- |
| TR/TE (ms) | 3.89/1.23 | 1720/87 | 4300/64 | 3.89/1.23 | 3.31/1.30 |
| Slice thickness/gap (mm) | 3/0.60 | 5/0.50 | 5/1.00 | 3/0.60 | 3/0.60 |
| FOV (mm) | 400 x 325 | 380 x 380 | 380 x 304 | 400 x 325 | 380 x 308 |
| Matrix | 176 x 288 | 320 x 320 | 128 x 128 | 176 x 288 | 195 x 320 |
| Flip angle (degrees) | 10 | 99 | 90 | 10 | 9 |
| b values (s/mm2) | Not applicable | Not applicable | 0, 50, 800 | Not applicable | Not applicable |

Note.- AP = arterial phase; DWI = diffusion-weighted imaging; EPI = echo-planar imaging; FOV = field of view; FS-T2WI = fat-suppressed T2-weighted imaging; PVP = portal venous phase; T1WI = T1-weighted imaging; TE = echo time; TR = repetition time. T1WI was supplemented according to the three-dimensional T1-weighted gradient-echo/VIBE sequence. DWI was supplemented according to the single-shot EPI sequence with b values of 0, 50, and 800 s/mm2.

**Supplementary Table S11. MRI feature definitions.**

This table defines all MRI-related variables in the final analysis dataset. Binary variables were coded as 1=present/positive and 0=absent/negative; main portal vein width was recorded as a continuous variable. For multifocal tumors, tumor size was based on the largest lesion diameter.

| **Variable** | **Feature** | **Imaging domain** | **Coding rule** | **Operational definition** | **Primary sequence/phase** | **Study role** | **Reference basis** |
| --- | --- | --- | --- | --- | --- | --- | --- |
| tumor_size_gt5cm | Tumor size >5 cm | Morphology / tumor burden | Binary; >5 cm=1, <=5 cm=0. For multifocal tumors, the largest lesion diameter was used. | Maximum visible tumor diameter measured on axial or multiplanar MR images. This variable reflects tumor burden and was retained in the final combined model. | T2WI and dynamic contrast-enhanced images | Final model feature | S11-1, S11-3 |
| satellite_nodule | Satellite nodule | Morphology / intrahepatic spread | Binary; present=1, absent=0. | A small tumor-suspicious nodule separated from the main tumor in the adjacent liver parenchyma or same/nearby segment, not representing a vessel, bile duct, cyst, or benign focus. | T2WI, DWI, and dynamic contrast-enhanced images | Final model feature | S11-3, S11-6 |
| infiltrative_appearance | Infiltrative appearance / ill-defined margin | Morphology / margin | Binary; present=1, absent=0. | Ill-defined, irregular, or infiltrative tumor-liver interface for which the lesion margin cannot be clearly separated from adjacent liver parenchyma; motion artifact or focal inflammatory change was not counted. | T2WI, DWI, and contrast-enhanced phases | Candidate MRI feature | S11-1, S11-2, S11-6 |
| intrahepatic_vessel_invasion | Intrahepatic vessel invasion | Vascular invasion | Binary; present=1, absent=0. | Tumor invasion of intrahepatic portal venous, hepatic venous, or branch vessels, shown by intravascular soft-tissue material, vessel expansion, enhancing intravascular tissue, or continuity with the tumor. Simple compression, narrowing, or bland thrombus was not counted. | Dynamic contrast-enhanced images, with T2WI/DWI when needed | Final model feature | S11-1, S11-5 |
| peritumoral_hypoenhancement_pvp | Peritumoral hypointensity on portal venous phase images | Peritumoral change | Binary; present=1, absent=0. | Band-like, wedge-shaped, or patchy hypointensity in liver parenchyma adjacent to the tumor on portal venous phase images, after excluding artifact, biliary dilatation, vascular perfusion difference, or background cirrhotic heterogeneity. | Portal venous phase | Final model feature | S11-3, S11-4 |
| rim_aphe | Rim arterial phase hyperenhancement | Enhancement pattern / LR-M-like | Binary; present=1, absent=0. | Peripheral ring-like or rim-dominant arterial phase hyperenhancement with relatively lower central enhancement, distinct from nonrim APHE. | Arterial phase | Candidate MRI feature | S11-1, S11-2 |
| incomplete_capsule | Incomplete capsule | Capsule / tumor boundary | Binary; incomplete=1, complete/not incomplete=0. | Capsule-like structure or capsule-like enhancement with focal defect, interruption, discontinuity, or local absence around the tumor. If no capsule was visible and interruption could not be assessed, coding followed the study rule. | Portal venous/delayed phase, with T2WI | Final model feature | S11-1, S11-2 |
| nodule_in_nodule | Nodule-in-nodule architecture | Internal architecture | Binary; present=1, absent=0. | A focal intratumoral nodule with signal intensity or enhancement pattern different from the surrounding tumor, suggesting a nodule-in-nodule or internal subnodule architecture. Hemorrhage, necrosis, or simple septation was not counted as a true internal nodule. | T1WI/T2WI, DWI, and dynamic contrast-enhanced images | Candidate MRI feature | S11-1, S11-2 |
| multiseptated_or_multinodular_architecture | Multiseptated or multinodular architecture | Internal architecture | Binary; present=1, absent=0. | Multiseptated, multilocular, mosaic, or multinodular internal architecture. Simple necrotic cavity or a small number of fibrous septa alone was not counted. | T2WI and dynamic contrast-enhanced images | Candidate MRI feature | S11-1, S11-2 |
| necrosis_or_severe_ischemia | Necrosis or severe ischemia | Internal content | Binary; present=1, absent=0. | Nonenhancing or markedly hypoenhancing intratumoral area compatible with liquefactive necrosis, coagulative necrosis, or severe ischemic change on contrast-enhanced images, interpreted with T2WI/DWI and differentiated from hemorrhage, fat, cystic change, or artifact. | Dynamic contrast-enhanced images, with T2WI/DWI | Candidate MRI feature | S11-1, S11-2 |
| intratumoral_artery | Intratumoral artery | Vascular sign | Binary; present=1, absent=0. | Linear, branching, or tubular arterial enhancing structure within the tumor, synchronous with arterial vascular enhancement. Exophytic peritumoral vessels and artifact were not counted. | Arterial phase | Candidate MRI feature | S11-1, S11-2 |
| nonrim_aphe | Nonrim arterial phase hyperenhancement | Major enhancement feature | Binary; present=1, absent=0. | Whole or partial arterial phase hyperenhancement of the solid tumor component relative to liver parenchyma, not predominantly rim-like. | Arterial phase | Candidate MRI feature | S11-1 |
| capsule_present | Visible capsule | Capsule / tumor boundary | Binary; present=1, absent=0. | A peripheral capsule-like rim, pseudocapsule, or circumscribing boundary visible around the tumor. This records capsule visibility and is distinct from capsule enhancement or capsule completeness. | T2WI and contrast-enhanced images | Candidate MRI feature | S11-1, S11-2 |
| capsule_enhancement | Capsule enhancement | Major enhancement feature | Binary; present=1, absent=0. | Smooth complete or near-complete peripheral rim enhancement on portal venous or delayed phase images, compatible with enhancing capsule and distinct from rim APHE or targetoid enhancement. | Portal venous/delayed phase | Candidate MRI feature | S11-1 |
| diffusion_restriction | Diffusion restriction | Ancillary feature | Binary; present=1, absent=0. | Hyperintensity on high-b-value DWI with corresponding relative hypointensity on the ADC map. T2 shine-through alone was not counted. | DWI and ADC map | Candidate MRI feature | S11-1, S11-2 |
| mild_moderate_t2_hyperintensity | Mild-to-moderate T2 hyperintensity | Ancillary feature | Binary; present=1, absent=0. | Tumor T2 signal intensity higher than background liver but lower than fluid or overt cystic component, distinct from marked T2 hyperintensity. | T2WI | Candidate MRI feature | S11-1, S11-2 |
| ring_enhancement | Ring enhancement | Enhancement pattern / targetoid | Binary; present=1, absent=0. | Peripheral ring-like enhancement after contrast administration with relatively lower central enhancement; interpreted according to enhancement phase and differentiated from capsule enhancement and rim APHE. | Dynamic contrast-enhanced images | Candidate MRI feature | S11-1, S11-2 |
| intratumoral_hemorrhage | Intratumoral hemorrhage | Internal content | Binary; present=1, absent=0. | Intratumoral signal compatible with blood products, such as T1 hyperintensity or T2/T2* hypointensity, after excluding fat, proteinaceous necrosis, and artifact. | T1WI, T2WI, and T2*/in-opposed phase when available | Candidate MRI feature | S11-1, S11-2 |
| intratumoral_fat | Intratumoral fat | Ancillary feature | Binary; present=1, absent=0. | Intratumoral signal loss on opposed-phase relative to in-phase images or signal drop on fat-suppressed images, suggesting fat within the lesion. | In-phase/opposed-phase T1WI and fat-suppressed images | Candidate MRI feature | S11-1, S11-2 |
| intratumoral_fat_deficiency_or_internal_nodule | Fat sparing/deficiency in tumor or internal nodule | Internal composition | Binary; present=1, absent=0. | Relative absence of fat within the tumor or internal nodule when background liver or another tumor component shows fat, suggesting internal heterogeneity or nodular transformation. | In-phase/opposed-phase T1WI and fat-suppressed images | Candidate MRI feature | S11-1, S11-2 |
| intratumoral_iron_deficiency_or_internal_nodule | Iron sparing/deficiency in tumor or internal nodule | Internal composition | Binary; present=1, absent=0. | Relative lack of iron-related signal loss within the tumor or internal nodule when background liver or other tumor tissue shows iron deposition. | T2WI, T2*, or in-phase/opposed-phase images | Candidate MRI feature | S11-1, S11-2 |
| focal_marked_t2_hyperintensity | Focal marked T2 hyperintensity | Ancillary / LR-M-like feature | Binary; present=1, absent=0. | Focal tumor area with T2 signal approaching fluid or clearly higher than the usual solid tumor component, suggesting necrosis, fluid, mucinous, or other special component. | T2WI | Candidate MRI feature | S11-1, S11-2 |
| intratumoral_iron_deposition_greater_than_liver | Intratumoral iron greater than liver | Internal composition | Binary; present=1, absent=0. | Intratumoral signal lower than background liver on T2/T2* or related sequences, suggesting greater iron deposition than surrounding liver parenchyma. | T2WI, T2*, or in-phase/opposed-phase images | Candidate MRI feature | S11-1, S11-2 |
| blood_pool_enhancement_pattern | Blood-pool enhancement pattern | Enhancement pattern | Binary; present=1, absent=0. | Dynamic enhancement pattern similar to blood pool across arterial, portal venous, and delayed phases, requiring full multiphasic assessment. | Dynamic contrast-enhanced images | Candidate MRI feature | S11-1, S11-2 |
| peripheral_washout | Peripheral washout | Targetoid feature | Binary; present=1, absent=0. | Peripheral tumor region becomes hypoenhancing relative to liver parenchyma on portal venous or delayed phase images, distinct from nonperipheral washout and capsule enhancement. | Portal venous/delayed phase | Candidate MRI feature | S11-1, S11-2 |
| delayed_central_enhancement | Delayed central enhancement | Targetoid feature | Binary; present=1, absent=0. | Central tumor region shows progressive or delayed enhancement compared with earlier phases, suggesting delayed central filling or fibrotic component. | Delayed phase | Candidate MRI feature | S11-1, S11-2 |
| targetoid_diffusion_restriction | Targetoid diffusion restriction | Targetoid feature | Binary; present=1, absent=0. | Targetoid pattern on DWI/ADC, usually with more pronounced peripheral restriction and different central signal, distinct from diffuse homogeneous diffusion restriction. | DWI and ADC map | Candidate MRI feature | S11-1, S11-2 |
| peritumoral_t2_hyperintensity | Peritumoral T2 hyperintensity | Peritumoral change | Binary; present=1, absent=0. | Band-like or patchy T2 hyperintensity in liver parenchyma adjacent to the tumor, after excluding vessels, ducts, and artifact. | T2WI | Candidate MRI feature | S11-1, S11-2 |
| aphe_ge50 | APHE extent >=50% | Enhancement extent | Binary; >=50%=1, <50%=0. | Arterial phase hyperenhancement involves approximately 50% or more of the viable solid tumor component. Necrotic, hemorrhagic, or nonenhancing areas were excluded from the estimate. | Arterial phase | Candidate MRI feature | S11-1, S11-3 |
| aphe_lt50 | APHE extent <50% | Enhancement extent | Binary; <50%=1, >=50%=0. | Arterial phase hyperenhancement involves less than approximately 50% of the viable solid tumor component. This variable is complementary to APHE extent >=50% when both are recorded. | Arterial phase | Candidate MRI feature | S11-1, S11-3 |
| ascites | Ascites | Background liver / portal hypertension | Binary; present=1, absent=0. | Free intraperitoneal fluid around the liver, spleen, bowel loops, or pelvis; minimal physiologic pelvic fluid was interpreted with clinical context. | T2WI and cross-sectional abdominal images | Candidate background variable | S11-1 |
| imaging_cirrhosis | Imaging cirrhosis | Background liver | Binary; present=1, absent=0. | Morphologic features of cirrhosis, including nodular liver surface, lobar redistribution, caudate or left lobe hypertrophy, widened fissures, and/or associated signs of portal hypertension. | T1WI/T2WI and contrast-enhanced images | Candidate background variable | S11-1 |
| diffuse_iron_overload | Diffuse hepatic iron overload | Background liver | Binary; present=1, absent=0. | Diffuse low signal of liver parenchyma on iron-sensitive sequences, consistent with hepatic iron deposition, interpreted with spleen, marrow, and clinical context when available. | T2WI, T2*, or in-phase/opposed-phase images | Candidate background variable | S11-1, S11-2 |
| diffuse_hepatic_steatosis | Diffuse hepatic steatosis | Background liver | Binary; present=1, absent=0. | Diffuse signal drop of liver parenchyma on opposed-phase relative to in-phase images, suggesting hepatic fat; iron-related signal change was excluded where possible. | In-phase/opposed-phase T1WI | Candidate background variable | S11-1, S11-2 |
| main_portal_vein_width_cm | Main portal vein width | Portal hypertension | Continuous variable; cm. | Maximum inner diameter of the main portal vein measured perpendicular to the vessel long axis, avoiding the bifurcation and oblique sections. | Portal venous phase | Candidate background variable | S11-1 |
| splenomegaly | Splenomegaly | Portal hypertension | Binary; present=1, absent=0. | Imaging impression of enlarged spleen based on size/volume and portal hypertension context; if a local threshold was used, the local threshold took precedence. | T2WI or contrast-enhanced axial/coronal images | Candidate background variable | S11-1 |
| portosystemic_shunt_or_collaterals | Portosystemic shunt or collateral vessels | Portal hypertension | Binary; present=1, absent=0. | Abnormal collateral vessels or shunting between portal venous and systemic venous systems, such as left gastric varices, splenorenal shunt, abdominal wall collaterals, or related pathways. | Portal venous phase | Candidate background variable | S11-1 |
| varices | Varices | Portal hypertension | Binary; present=1, absent=0. | Tortuous dilated venous structures at the esophagogastric junction, perigastric region, splenic hilum, abdominal wall, or other sites compatible with portal hypertensive varices. | Portal venous phase | Candidate background variable | S11-1 |

**References**

S11-1. Chernyak V, Fowler KJ, Kamaya A, Kielar AZ, Elsayes KM, Bashir MR, et al. Liver Imaging Reporting and Data System (LI-RADS) Version 2018: Imaging of Hepatocellular Carcinoma in At-Risk Patients. Radiology. 2018;289(3):816-830. doi:10.1148/radiol.2018181494.

S11-2. Chernyak V, Santillan CS, Papadatos D, Sirlin CB. LI-RADS ancillary features on CT and MRI. Abdom Radiol (NY). 2018;43(1):82-100. doi:10.1007/s00261-017-1220-6.

S11-3. An C, Kim DW, Park YN, Chung YE, Rhee H, Kim MJ. Single Hepatocellular Carcinoma: Preoperative MR Imaging to Predict Early Recurrence after Curative Resection. Radiology. 2015;276(2):433-443. doi:10.1148/radiol.15142394.

S11-4. Kim KA, Kim MJ, Jeon HM, Kim KS, Choi JS, Ahn SH, et al. Prediction of microvascular invasion of hepatocellular carcinoma: usefulness of peritumoral hypointensity seen on gadoxetate disodium-enhanced hepatobiliary phase images. J Magn Reson Imaging. 2012;35(3):629-634. doi:10.1002/jmri.22876.

S11-5. Griffin N, Addley H, Sala E, Shaw AS, Grant LA, Punwani S, et al. Vascular invasion in hepatocellular carcinoma: is there a correlation with MRI? Br J Radiol. 2012;85(1014):736-744. doi:10.1259/bjr/94924398.

S11-6. Ariizumi S, Kitagawa K, Kotera Y, Takahashi Y, Katagiri S, Kuwatsuru R, et al. A non-smooth tumor margin in the hepatobiliary phase of gadoxetic acid disodium-enhanced magnetic resonance imaging predicts microscopic portal vein invasion, intrahepatic metastasis, and early recurrence after hepatectomy in patients with hepatocellular carcinoma. J Hepatobiliary Pancreat Sci. 2011;18(4):575-585. doi:10.1007/s00534-010-0369-y.

**Supplementary Table S12. Terminology glossary.**

| **Term** | **Abbreviation** | **Definition** | **Usage note** |
| --- | --- | --- | --- |
| Development cohort |  | Patients from the Affiliated Hospital of North Sichuan Medical College used for variable screening, model development, internal validation, and cutoff determination. | Use consistently instead of primary center in the submission manuscript. |
| External validation cohort |  | Independent patients from Suining Central Hospital used to evaluate model transportability without refitting the model. | Use consistently instead of external test set or test cohort unless journal style requires otherwise. |
| Early recurrence |  | Recurrence confirmed by imaging or pathology within 24 months after curative hepatectomy. | This is the primary outcome definition. |
| Late recurrence |  | Recurrence confirmed more than 24 months after curative hepatectomy. | Used only when describing recurrence timing. |
| Recurrence |  | New intrahepatic HCC lesion and/or extrahepatic metastasis detected after curative hepatectomy during follow-up. | AFP elevation alone is not sufficient for diagnosis. |
| Disease-free survival | DFS | Time from curative hepatectomy to first confirmed recurrence; patients without recurrence were censored according to prespecified rules. | Define at first use. |
| 24-month predicted probability of early recurrence |  | Model-estimated probability of recurrence within 24 months after surgery. | Use instead of vague phrases such as 24-month risk. |
| Harrell C-index | C-index | A discrimination measure for censored survival outcomes. | Reported for Cox-model discrimination. |
| 24-month time-dependent AUC | AUC | Time-specific discrimination at 24 months for censored survival data. | Use 24-month AUC only after definition. |
| Brier score |  | A measure of prediction error for probabilistic predictions; lower values indicate smaller prediction error. | Reported at 24 months. |
| Final combined model |  | The final Cox model combining preoperative clinical/laboratory and MRI features. | Use consistently for the main model. |
| Internal validation |  | Bootstrap validation performed within the development cohort. | Do not describe as random train-test splitting. |
| External validation |  | Application of the fixed development-cohort model to the independent external validation cohort. | No model refitting in the external cohort. |
| Exploratory risk cutoff |  | Development-cohort cutoff of 0.584 derived from Youden index for risk stratification. | For risk communication and research stratification only; not a stand-alone treatment threshold. |
| High-risk group |  | Patients with predicted probability >=0.584 according to the final combined model. | Derived using the exploratory development-cohort cutoff. |
| Low-risk group |  | Patients with predicted probability <0.584 according to the final combined model. | Derived using the exploratory development-cohort cutoff. |
| Decision curve analysis | DCA | A method for estimating clinical net benefit across threshold probabilities. | Used to compare model utility across risk thresholds. |
| Postoperative adjuvant therapy |  | Postoperative treatment analyzed only in sensitivity analysis, not included in the preoperative main model. | Do not interpret sensitivity analysis as causal evidence of treatment efficacy. |

Note.- This English-only glossary is intended for submission-package consistency. A Chinese reading copy is retained outside the submission package.

**Supplementary Table S13. TRIPOD checklist for the prediction model study.**

Checklist based on the TRIPOD 2015 reporting statement for multivariable prediction model development and validation. Locations are given by manuscript section because journal page numbers are not finalized.

| **Section** | **Item** | **Topic** | **TRIPOD reporting recommendation** | **Location in manuscript/supplement** | **Reporting status** | **Notes for this study** |
| --- | --- | --- | --- | --- | --- | --- |
| Title and abstract | 1 | Title | Identify the study as developing and/or validating a multivariable prediction model, the target population, and the outcome to be predicted. | Title page | Reported | The Chinese title identifies MRI-clinical factors and early recurrence after hepatectomy for HCC. |
| Title and abstract | 2 | Abstract | Provide a summary of objectives, study design, setting, participants, sample size, predictors, outcome, statistical analysis, results, and conclusions. | Structured abstract | Reported | Abstract includes two-center retrospective design, cohorts, final predictors, performance, and cautious conclusion. |
| Introduction | 3a | Background and context | Explain the medical context, intended use, and whether the model is diagnostic or prognostic. | Introduction, paragraphs 1-3 | Reported | The model is prognostic and intended for preoperative early-recurrence risk estimation. |
| Introduction | 3b | Objectives | Specify the objectives, including whether the study concerns model development, validation, or updating. | Introduction, final paragraph | Reported | The study develops and externally validates a preoperative MRI-clinical model. |
| Methods | 4a | Source of data | Describe the source of data and whether it was collected prospectively or retrospectively. | Methods: Study design and Study patients | Reported | Two-center retrospective cohort; development cohort and external validation cohort are specified. |
| Methods | 4b | Study dates | Specify key study dates, including start of accrual, end of accrual, and, if applicable, end of follow-up. | Methods: Study patients; Follow-up and endpoint | Reported | Accrual from January 2018 to December 2025; last follow-up April 1, 2026. |
| Methods | 5a | Eligibility criteria | Specify key study inclusion and exclusion criteria. | Methods: Study patients; Figure 1 | Reported | Inclusion/exclusion criteria and patient-selection flow are described. |
| Methods | 5b | Treatments received | Describe treatments received, if relevant. | Methods: Study patients; Predictors and data processing; Sensitivity analysis | Reported | Curative hepatectomy is required; prior HCC treatment is excluded; postoperative adjuvant therapy is handled in sensitivity analysis. |
| Methods | 6a | Outcome definition | Clearly define the outcome predicted by the model, including how and when it was assessed. | Methods: Follow-up and endpoint | Reported | Early recurrence is recurrence within 24 months after curative hepatectomy; imaging/pathologic confirmation is described. |
| Methods | 6b | Outcome blinding | Report actions to blind outcome assessment to candidate predictors, if applicable. | Methods: Follow-up and endpoint | Reported | Follow-up imaging was assessed independently by abdominal radiologists; recurrence diagnosis did not rely on AFP alone. |
| Methods | 7a | Predictor definition | Clearly define all candidate predictors, including how and when they were measured. | Methods: MRI acquisition and image evaluation; Predictors and data processing; Supplementary Table S11 | Reported | Clinical/laboratory timing, MRI feature coding, tumor size handling, and feature definitions are described. |
| Methods | 7b | Predictor blinding | Report actions to blind predictor assessment to outcome and other information, if applicable. | Methods: MRI acquisition and image evaluation | Reported | MRI readers were blinded to recurrence outcome, pathology, and laboratory data. |
| Methods | 8 | Sample size | Explain how the study size was arrived at. | Methods: Study patients; Statistical analysis | Partially reported | The cohort reflects eligible consecutive retrospective cases and event-number constraints were considered; no formal a priori sample-size calculation was performed. |
| Methods | 9 | Missing data | Describe how missing data were handled. | Methods: Predictors and data processing | Reported | After data re-collection, final modeling variables had no missing values. |
| Methods | 10a | Model development methods | Describe statistical methods used to develop the model, including predictor handling. | Methods: Statistical analysis | Reported | Cox regression, transformations, binary coding, and model-building strategy are described. |
| Methods | 10b | Model assumptions | Describe methods for checking model assumptions. | Methods: Statistical analysis | Reported | Schoenfeld residuals were used for proportional hazards assumption; collinearity and distribution checks were also described. |
| Methods | 10c | Predictor selection | Describe methods for selecting predictors during multivariable modeling. | Methods: Statistical analysis | Reported | Univariable screening, clinical relevance, collinearity assessment, and AIC-based backward selection are described. |
| Methods | 10d | Internal validation | Describe methods for internal validation. | Methods: Statistical analysis | Reported | Bootstrap internal validation was performed, with B=200 testing and B=1000 formal validation. |
| Methods | 10e | External validation | For validation, describe how predictions were calculated. | Methods: Statistical analysis | Reported | Fixed coefficients from the development cohort were applied directly to the external validation cohort without refitting. |
| Methods | 10f | Model updating | If model updating was performed, describe methods. | Methods: Statistical analysis | Not applicable | No model updating or recalibration was performed in the external validation cohort. |
| Methods | 11 | Risk groups | Provide details on how risk groups were created, if done. | Methods: Statistical analysis; Results: Risk stratification | Reported | The exploratory cutoff of 0.584 was derived by Youden index in the development cohort and applied to the external validation cohort. |
| Methods | 12 | Analysis software | State the statistical software used. | Methods: Statistical analysis | Reported | R 4.5.2 and relevant package versions are reported. |
| Results | 13a | Participant flow | Describe the flow of participants through the study, including exclusions. | Results: Patient inclusion and cohort characteristics; Figure 1 | Reported | Screened, excluded, follow-up eligible, follow-up missing, and final analyzed patients are reported. |
| Results | 13b | Participant characteristics | Describe participant characteristics. | Results: Baseline characteristics; Table 1 | Reported | Baseline characteristics are reported for all patients, the development cohort, and the external validation cohort. |
| Results | 13c | Validation comparison | For validation, show comparison with development data. | Results: Baseline differences; Table 1 | Reported | Development and external validation cohorts are compared. |
| Results | 14a | Numbers analyzed | Report numbers of participants and outcome events in each analysis. | Results: Patient inclusion and cohort characteristics | Reported | Final sample size and early recurrence counts are reported for both cohorts. |
| Results | 14b | Unadjusted associations | Report unadjusted associations between candidate predictors and outcome, if relevant. | Results: Early versus non-early recurrence; Supplementary Tables S3-S4 | Reported | Group comparisons and univariable Cox/logistic analyses are available. |
| Results | 15a | Model specification | Present the full prediction model to allow individual predictions. | Results: Final combined model; Figure 2; Web calculator | Reported | Final predictors, coefficients/HRs, baseline survival, nomogram, and web calculator are provided. |
| Results | 15b | Model use | Explain how to use the prediction model. | Results: Nomogram and web calculator; Figure 2 | Reported | Nomogram and web calculator instructions are described. |
| Results | 16 | Model performance | Report model performance measures with confidence intervals when available. | Results: Internal/external validation; Tables 4-5; Figures 3-4 | Reported | C-index, 24-month AUC, Brier score, calibration, and model comparisons are reported. |
| Results | 17 | Model updating results | If updating was performed, report updated model performance. | Results | Not applicable | No model updating was performed. |
| Discussion | 18 | Limitations | Discuss limitations of the study. | Discussion, limitations paragraph | Reported | Retrospective design, external sample size, external calibration, population scope, pathology variables, and MRI reader dependence are discussed. |
| Discussion | 19a | Interpretation | Provide an overall interpretation of results, considering objectives, limitations, and previous studies. | Discussion, paragraphs 1-4 | Reported | Main findings, predictor meaning, prior-model comparison, external validation, calibration, DCA, and sensitivity analysis are interpreted. |
| Discussion | 19b | Implications | Discuss potential clinical use and implications. | Discussion; Conclusion | Reported | The model is positioned as a preoperative risk-stratification and research communication tool, not a stand-alone treatment decision tool. |
| Other information | 20 | Supplementary information | Provide information about availability of supplementary resources, if applicable. | Main text; Supplementary materials package | Reported | Supplementary tables/figures, MRI feature definitions, terminology glossary, TRIPOD checklist, and web calculator package are provided. |
| Other information | 21 | Funding | Report funding source and role of funders. | Declarations | Reported | Funding sources are listed. Funders’ role is not specifically stated; add if required by target journal. |

Note.- TRIPOD = Transparent Reporting of a multivariable prediction model for Individual Prognosis Or Diagnosis; HCC = hepatocellular carcinoma; MRI = magnetic resonance imaging; AUC = area under the receiver operating characteristic curve; DCA = decision curve analysis.

**Supplementary Table S14. Model performance metrics with 95% confidence intervals.**

| **Validation setting / cohort** | **N** | **Events** | **C-index (95% CI)** | **24-month AUC (95% CI)** | **24-month Brier score (95% CI)** | **Calibration slope (95% CI)** | **Calibration intercept (95% CI)** |
| --- | --- | --- | --- | --- | --- | --- | --- |
| Development apparent | 258 | 153 | 0.756 (0.715 to 0.796) | 0.838 (0.789 to 0.882) | 0.163 (0.141 to 0.185) | 1.060 (0.755 to 1.533) | -0.016 (-0.309 to 0.276) |
| Bootstrap-corrected internal validation | 258 | 153 | 0.749 (0.710 to 0.792) | 0.829 (0.814 to 0.843) | 0.169 (0.159 to 0.175) | 0.956 (0.719 to 1.217) | -0.014 (-0.297 to 0.287) |
| External validation | 62 | 30 | 0.892 (0.841 to 0.937) | 0.978 (0.945 to 1.000) | 0.087 (0.060 to 0.120) | 2.705 (1.882 to 46.376) | -1.288 (-12.850 to -0.067) |

Note.- CI = confidence interval; AUC = area under the receiver operating characteristic curve. Internal-validation intervals reused the existing B=1000 bootstrap output. External-validation intervals were estimated by patient-level bootstrap resampling with B=2000 using fixed development-cohort model predictions; the model was not refitted.

**Supplementary Table S15. Full baseline characteristics of the development and external validation cohorts.**

*Comparison of the model development cohort and external validation cohort.*

| **Characteristic** | **All Patients (n = 320)** | **Development Cohort (n = 258)** | **External Validation Cohort (n = 62)** | **P Value** | **Test** |
| --- | --- | --- | --- | --- | --- |
| **Clinical and Laboratory Characteristics** |  |  |  |  |  |
| Chronic hepatitis B, yes | 227 (70.9) | 196 (76.0) | 31 (50.0) | <.001 | Chi-square |
| log(CEA)* | 1.14 (0.80-1.69) | 1.14 (0.77-1.69) | 1.20 (0.89-1.69) | .208 | Mann-Whitney U |
| log(CA19-9)* | 2.92 (2.39-3.61) | 2.90 (2.35-3.52) | 3.14 (2.67-3.80) | .122 | Mann-Whitney U |
| log(AFP)* | 3.57 (1.73-6.84) | 3.69 (1.79-7.06) | 2.70 (1.56-5.65) | .036 | Mann-Whitney U |
| AFP >400, yes | 98 (30.6) | 83 (32.2) | 15 (24.2) | .221 | Chi-square |
| log(PIVKA-II)* | 5.58 (3.45-8.25) | 5.61 (3.77-7.84) | 4.19 (1.64-9.35) | .121 | Mann-Whitney U |
| AST >40, yes | 151 (47.2) | 121 (46.9) | 30 (48.4) | .833 | Chi-square |
| ALT* | 34 (22-56) | 34 (22-55) | 35 (24-78) | .145 | Mann-Whitney U |
| log(ALP)* | 4.61 (4.34-4.91) | 4.61 (4.35-4.91) | 4.62 (4.32-4.90) | .656 | Mann-Whitney U |
| log(GGT)* | 4.08 (3.50-4.82) | 4.06 (3.50-4.80) | 4.12 (3.47-4.96) | .745 | Mann-Whitney U |
| Prothrombin time* | 13.5 (12.5-14.8) | 13.3 (12.5-14.2) | 16.9 (15.3-18.1) | <.001 | Mann-Whitney U |
| Platelets* | 150 (107-200) | 151 (105-198) | 148 (109-209) | .461 | Mann-Whitney U |
| NLR* | 2.68 (1.82-3.82) | 2.66 (1.77-3.71) | 3.22 (2.19-5.10) | .034 | Mann-Whitney U |
| log(NLR)* | 1.30 (1.04-1.57) | 1.30 (1.02-1.55) | 1.44 (1.16-1.81) | .034 | Mann-Whitney U |
| HBsAb positive, yes | 59 (18.4) | 36 (14.0) | 23 (37.1) | <.001 | Chi-square |
| HBeAg positive, yes | 33 (10.3) | 22 (8.5) | 11 (17.7) | .032 | Chi-square |
| Total bilirubin* | 15.9 (12.0-20.7) | 16.0 (12.2-20.5) | 15.1 (11.3-23.0) | .941 | Mann-Whitney U |
| AST/ALT ratio* | 1.16 (0.84-1.52) | 1.18 (0.90-1.56) | 1.07 (0.65-1.39) | .010 | Mann-Whitney U |
| ALBI grade 2 or 3, yes | 106 (33.1) | 89 (34.5) | 17 (27.4) | .288 | Chi-square |
| Postoperative adjuvant therapy, yes | 183 (57.2) | 131 (50.8) | 52 (83.9) | <.001 | Chi-square |
| **Preoperative MRI Characteristics** |  |  |  |  |  |
| Tumor size >5 cm, yes | 148 (46.2) | 118 (45.7) | 30 (48.4) | .707 | Chi-square |
| Satellite nodule, yes | 52 (16.2) | 44 (17.1) | 8 (12.9) | .426 | Chi-square |
| Infiltrative appearance, yes | 105 (32.8) | 87 (33.7) | 18 (29.0) | .480 | Chi-square |
| Intrahepatic vessel invasion, yes | 46 (14.4) | 39 (15.1) | 7 (11.3) | .441 | Chi-square |
| Peritumoral hypointensity on portal venous phase images, yes | 71 (22.2) | 56 (21.7) | 15 (24.2) | .672 | Chi-square |
| Rim arterial phase hyperenhancement, yes | 212 (66.2) | 177 (68.6) | 35 (56.5) | .069 | Chi-square |
| Incomplete capsule, yes | 218 (68.1) | 171 (66.3) | 47 (75.8) | .148 | Chi-square |
| Nodule-in-nodule architecture, yes | 73 (22.8) | 65 (25.2) | 8 (12.9) | .038 | Chi-square |
| Multiseptated or multinodular architecture, yes | 151 (47.2) | 116 (45.0) | 35 (56.5) | .104 | Chi-square |
| Necrosis or severe ischemia, yes | 156 (48.8) | 119 (46.1) | 37 (59.7) | .055 | Chi-square |
| Intratumoral artery, yes | 102 (31.9) | 70 (27.1) | 32 (51.6) | <.001 | Chi-square |
| Nonrim arterial phase hyperenhancement, yes | 186 (58.1) | 151 (58.5) | 35 (56.5) | .766 | Chi-square |
| Capsule present, yes | 237 (74.1) | 196 (76.0) | 41 (66.1) | .112 | Chi-square |
| Capsule enhancement, yes | 234 (73.1) | 193 (74.8) | 41 (66.1) | .166 | Chi-square |
| Diffusion restriction, yes | 316 (98.8) | 254 (98.4) | 62 (100.0) | 1.000 | Fisher exact |
| Mild-to-moderate T2 hyperintensity, yes | 280 (87.5) | 222 (86.0) | 58 (93.5) | .109 | Chi-square |
| Ring enhancement, yes | 85 (26.6) | 68 (26.4) | 17 (27.4) | .865 | Chi-square |
| Intratumoral hemorrhage, yes | 128 (40.0) | 106 (41.1) | 22 (35.5) | .419 | Chi-square |
| Intratumoral fat, yes | 196 (61.3) | 170 (65.9) | 26 (41.9) | <.001 | Chi-square |
| Intratumoral fat deficiency or internal nodule, yes | 47 (14.7) | 35 (13.6) | 12 (19.4) | .248 | Chi-square |
| Intratumoral iron deficiency or internal nodule, yes | 268 (83.8) | 222 (86.0) | 46 (74.2) | .023 | Chi-square |
| Focal marked T2 hyperintensity, yes | 188 (58.8) | 149 (57.8) | 39 (62.9) | .459 | Chi-square |
| Intratumoral iron deposition greater than liver, yes | 16 (5.0) | 12 (4.7) | 4 (6.5) | .524 | Fisher exact |
| Blood-pool enhancement pattern, yes | 24 (7.5) | 23 (8.9) | 1 (1.6) | .058 | Fisher exact |
| Peripheral washout, yes | 284 (88.8) | 229 (88.8) | 55 (88.7) | .991 | Chi-square |
| Delayed central enhancement, yes | 16 (5.0) | 14 (5.4) | 2 (3.2) | .746 | Fisher exact |
| Targetoid diffusion restriction, yes | 26 (8.1) | 20 (7.8) | 6 (9.7) | .618 | Chi-square |
| Peritumoral T2 hyperintensity, yes | 92 (28.7) | 75 (29.1) | 17 (27.4) | .797 | Chi-square |
| APHE proportion <50%, yes | 107 (33.4) | 86 (33.3) | 21 (33.9) | .936 | Chi-square |
| Ascites, yes | 42 (13.1) | 24 (9.3) | 18 (29.0) | <.001 | Chi-square |
| Imaging cirrhosis, yes | 138 (43.1) | 109 (42.2) | 29 (46.8) | .518 | Chi-square |
| Diffuse iron overload, yes | 44 (13.8) | 17 (6.6) | 27 (43.5) | <.001 | Chi-square |
| Diffuse hepatic steatosis, yes | 98 (30.6) | 94 (36.4) | 4 (6.5) | <.001 | Chi-square |
| Main portal vein width (cm)* | 1.3 (1.1-1.4) | 1.3 (1.1-1.4) | 1.4 (1.3-1.5) | <.001 | Mann-Whitney U |
| Splenomegaly, yes | 68 (21.2) | 58 (22.5) | 10 (16.1) | .272 | Chi-square |
| Portosystemic shunt/collaterals, yes | 25 (7.8) | 25 (9.7) | 0 (0.0) | .007 | Fisher exact |
| Varices, yes | 32 (10.0) | 30 (11.7) | 2 (3.2) | .047 | Chi-square |
| **Follow-up and Endpoint** |  |  |  |  |  |
| Disease-free survival (months)* | 18.9 (9.7-32.5) | 17.1 (8.8-32.4) | 24.9 (13.1-32.4) | .086 | Mann-Whitney U |
| Early recurrence (<24 months), yes | 183 (57.2) | 153 (59.3) | 30 (48.4) | .119 | Chi-square |

Note.-Unless otherwise indicated, categorical variables are presented as n (%) for the positive/present category. P values compare the development cohort with the external validation cohort; the All Patients column is descriptive only. Categorical variables were compared with the chi-square test or Fisher exact test, as appropriate. Percentages are calculated using non-missing denominators.

* Continuous variables are presented as medians with IQRs in parentheses and were compared with the Mann-Whitney U test. APHE = arterial phase hyperenhancement; DFS = disease-free survival; NLR = neutrophil-to-lymphocyte ratio.

**Supplementary Table S16. Full comparison of patients with and without early recurrence in the development cohort.**

*Development cohort only; postoperative adjuvant therapy was reserved for sensitivity analysis and excluded from this main screening table.*

| **Characteristic** | **Non-early Recurrence (n = 105)** | **Early Recurrence (n = 153)** | **P Value** | **Test** |
| --- | --- | --- | --- | --- |
| **Clinical and Laboratory Characteristics** |  |  |  |  |
| Chronic hepatitis B, yes | 78 (74.3) | 118 (77.1) | .600 | Chi-square |
| log(CEA)* | 1.02 (0.68-1.39) | 1.26 (0.85-2.92) | <.001 | Mann-Whitney U |
| log(CA19-9)* | 2.79 (2.33-3.08) | 3.09 (2.40-5.92) | <.001 | Mann-Whitney U |
| log(AFP)* | 2.97 (1.70-5.65) | 4.82 (1.82-7.91) | .011 | Mann-Whitney U |
| AFP >400, yes | 24 (22.9) | 59 (38.6) | .008 | Chi-square |
| log(PIVKA-II)* | 4.40 (2.06-5.83) | 6.67 (5.04-9.20) | <.001 | Mann-Whitney U |
| AST >40, yes | 42 (40.0) | 79 (51.6) | .066 | Chi-square |
| ALT* | 33 (24-55) | 34 (21-55) | .633 | Mann-Whitney U |
| log(ALP)* | 4.52 (4.32-4.80) | 4.70 (4.36-4.93) | .039 | Mann-Whitney U |
| log(GGT)* | 3.74 (3.37-4.47) | 4.25 (3.66-4.96) | <.001 | Mann-Whitney U |
| Prothrombin time* | 13.3 (12.4-14.0) | 13.4 (12.5-14.2) | .670 | Mann-Whitney U |
| Platelets* | 139 (93-187) | 156 (114-203) | .024 | Mann-Whitney U |
| NLR* | 2.62 (1.74-3.78) | 2.70 (1.89-3.47) | .790 | Mann-Whitney U |
| log(NLR)* | 1.29 (1.01-1.57) | 1.31 (1.06-1.50) | .790 | Mann-Whitney U |
| HBsAb positive, yes | 16 (15.2) | 20 (13.1) | .622 | Chi-square |
| HBeAg positive, yes | 9 (8.6) | 13 (8.5) | .983 | Chi-square |
| Total bilirubin* | 17.0 (13.1-20.7) | 15.0 (11.6-20.4) | .075 | Mann-Whitney U |
| AST/ALT ratio* | 1.05 (0.79-1.33) | 1.27 (0.93-1.66) | .001 | Mann-Whitney U |
| ALBI grade 2 or 3, yes | 28 (26.7) | 61 (39.9) | .028 | Chi-square |
| **Preoperative MRI Characteristics** |  |  |  |  |
| Tumor size >5 cm, yes | 26 (24.8) | 92 (60.1) | <.001 | Chi-square |
| Satellite nodule, yes | 6 (5.7) | 38 (24.8) | <.001 | Chi-square |
| Infiltrative appearance, yes | 18 (17.1) | 69 (45.1) | <.001 | Chi-square |
| Intrahepatic vessel invasion, yes | 6 (5.7) | 33 (21.6) | <.001 | Chi-square |
| Peritumoral hypointensity on portal venous phase images, yes | 5 (4.8) | 51 (33.3) | <.001 | Chi-square |
| Rim arterial phase hyperenhancement, yes | 74 (70.5) | 103 (67.3) | .592 | Chi-square |
| Incomplete capsule, yes | 54 (51.4) | 117 (76.5) | <.001 | Chi-square |
| Nodule-in-nodule architecture, yes | 11 (10.5) | 54 (35.3) | <.001 | Chi-square |
| Multiseptated or multinodular architecture, yes | 29 (27.6) | 87 (56.9) | <.001 | Chi-square |
| Necrosis or severe ischemia, yes | 38 (36.2) | 81 (52.9) | .008 | Chi-square |
| Intratumoral artery, yes | 17 (16.2) | 53 (34.6) | .001 | Chi-square |
| Nonrim arterial phase hyperenhancement, yes | 59 (56.2) | 92 (60.1) | .528 | Chi-square |
| Capsule present, yes | 89 (84.8) | 107 (69.9) | .006 | Chi-square |
| Capsule enhancement, yes | 87 (82.9) | 106 (69.3) | .014 | Chi-square |
| Diffusion restriction, yes | 103 (98.1) | 151 (98.7) | 1.000 | Fisher exact |
| Mild-to-moderate T2 hyperintensity, yes | 93 (88.6) | 129 (84.3) | .332 | Chi-square |
| Ring enhancement, yes | 21 (20.0) | 47 (30.7) | .055 | Chi-square |
| Intratumoral hemorrhage, yes | 34 (32.4) | 72 (47.1) | .019 | Chi-square |
| Intratumoral fat, yes | 69 (65.7) | 101 (66.0) | .960 | Chi-square |
| Intratumoral fat deficiency or internal nodule, yes | 15 (14.3) | 20 (13.1) | .780 | Chi-square |
| Intratumoral iron deficiency or internal nodule, yes | 94 (89.5) | 128 (83.7) | .182 | Chi-square |
| Focal marked T2 hyperintensity, yes | 64 (61.0) | 85 (55.6) | .389 | Chi-square |
| Intratumoral iron deposition greater than liver, yes | 5 (4.8) | 7 (4.6) | 1.000 | Fisher exact |
| Blood-pool enhancement pattern, yes | 11 (10.5) | 12 (7.8) | .466 | Chi-square |
| Peripheral washout, yes | 97 (92.4) | 132 (86.3) | .127 | Chi-square |
| Delayed central enhancement, yes | 5 (4.8) | 9 (5.9) | .696 | Chi-square |
| Targetoid diffusion restriction, yes | 8 (7.6) | 12 (7.8) | .947 | Chi-square |
| Peritumoral T2 hyperintensity, yes | 14 (13.3) | 61 (39.9) | <.001 | Chi-square |
| APHE proportion <50%, yes | 25 (23.8) | 61 (39.9) | .007 | Chi-square |
| Ascites, yes | 10 (9.5) | 14 (9.2) | .919 | Chi-square |
| Imaging cirrhosis, yes | 44 (41.9) | 65 (42.5) | .926 | Chi-square |
| Diffuse iron overload, yes | 8 (7.6) | 9 (5.9) | .581 | Chi-square |
| Diffuse hepatic steatosis, yes | 38 (36.2) | 56 (36.6) | .946 | Chi-square |
| Main portal vein width (cm)* | 1.2 (1.1-1.4) | 1.3 (1.1-1.4) | .919 | Mann-Whitney U |
| Splenomegaly, yes | 23 (21.9) | 35 (22.9) | .854 | Chi-square |
| Portosystemic shunt/collaterals, yes | 10 (9.5) | 15 (9.8) | .940 | Chi-square |
| Varices, yes | 13 (12.5) | 17 (11.1) | .734 | Chi-square |

Note.-Categorical variables are presented as n (%) for the positive/present category. Continuous variables are presented as medians with IQRs in parentheses. P values compare non-early recurrence and early recurrence groups in the development cohort. Categorical variables were compared with the chi-square test or Fisher exact test, as appropriate; continuous variables were compared with the Mann-Whitney U test. Postoperative adjuvant therapy was not included in this main screening table.

APHE = arterial phase hyperenhancement; NLR = neutrophil-to-lymphocyte ratio.

# 16 Supplementary Web Calculator

The file Web_Calculator_Early_Recurrence.zip is provided as a separate supplementary package. It contains an offline static web calculator for estimating 24-month early recurrence probability and assigning the corresponding risk group based on the final combined model. The calculator package is not embedded in this Word file and should be uploaded separately if required by the submission system.
